# Context-dependent Activities of Mitrephorone Link Lipid Redirection, Anti-inflammatory Action, and Ferroptosis Control to Hepatocyte Protection

**DOI:** 10.7150/thno.127100

**Published:** 2026-03-17

**Authors:** Lorenz Waltl, Lukas A. Wein, Leonhard Bereuter, Fengting Su, Henriett Barta, Loc Le Xuan, David Holubek, Zahra Mahmoudi Eshkaftaki, Katharina Puskac, Anita Siller, Peter Schlenke, Harald Schennach, Eva-Maria Pferschy-Wenzig, Hans Schött, Silvia Racedo, Solveigh C. Koeberle, Thomas Magauer, Andreas Koeberle

**Affiliations:** 1 Michael Popp Institute and Center for Molecular Biosciences (CMBI), University of Innsbruck, Mitterweg 24, 6020 Innsbruck, Austria.; 2 Institute of Human Genetics, Medical University of Innsbruck, Peter-Mayr-Straße 1, 6020 Innsbruck, Austria.; 3 Department of Organic Chemistry and Center for Molecular Biosciences (CMBI) University of Innsbruck, Innrain 80-82, 6020 Innsbruck, Austria.; 4 Institute of Pharmaceutical Sciences and Excellence Field BioHealth, NAWI Graz, University of Graz, Beethovenstraße 8, 8010 Graz, Austria.; 5 Central Institute for Blood Transfusion and Immunology, Tirol Kliniken GmbH, Anichstraße 35, 6020 Innsbruck, Austria.; 6 Clinical Department of Blood Group Serology and Transfusion Medicine, Medical University of Graz, Auenbruggerplatz 48, 8036 Graz, Austria.

## Abstract

**Rationale:**

Liver diseases are driven by aberrant metabolism, involve necrotic cell death, particularly ferroptosis, and progress with low-grade inflammation. Rationally designed small molecules that simultaneously target these processes are lacking. Here, we investigated mitrephorone B, an ent-trachylobane diterpenoid from the Bornean shrub *Mitrephora glabra*, accessible by total synthesis, as a potential hepatoprotective agent *in vitro*.

**Methods:**

We tested mitrephorone B and four derivatives in human HepaRG hepatocytes, primary peripheral blood mononuclear cells (PBMCs), polarized monocyte-derived macrophages, and lipid-driven *in vitro* disease models. Quantitative lipidomics, cell viability and membrane integrity assays, overexpression studies, immunodetection, molecular probes, expression analysis, and cell-free activity assays were used to assess effects on programmed cell death, lipid mediator biosynthesis, cytokine expression, and alternations of the cellular lipidome.

**Results:**

Mitrephorone B reduced pro-inflammatory cytokine expression in PBMCs independently of nuclear factor-κB signaling, inhibited phospholipid peroxidation, and suppressed ferroptosis in hepatocytes, associated with altered triglyceride fatty acid composition in PBMCs. It lowered cholesteryl ester levels in PBMCs and cocultures with hepatocytes and suppressed pro-inflammatory leukotriene production by antagonizing 5-lipoxygenase-activating protein. Over time, mitrephorone B limited the capacity of PBMCs to generate pro-inflammatory lipid mediators while modestly promoting the formation of epoxyeicosatrienoic acids, known to counteract inflammation and cell death. In an immunocompetent *in vitro* model of lipid-induced hepatotoxicity, it improved metabolic activity and reduced triglyceride content. Structurally, the 9-oxo group was essential for effective 5-lipoxygenase-activating protein antagonism, while the 10-oxo group contributed to cytokine suppression. Anti-ferroptotic activity was largely preserved across derivatives, whereas small structural modifications fine-tuned lipidome effects.

**Conclusions:**

Mitrephorone B displays a unique activity profile, redirecting neutral lipid accumulation, suppressing ferroptosis, and inhibiting inflammation. These properties highlight its potential as a candidate lead structure for treating necroinflammatory liver diseases associated with aberrant lipid metabolism, including metabolic dysfunction-associated steatotic liver disease, steatohepatitis, cirrhosis, and hepatocellular carcinoma.

## Introduction

The liver is a central hub of glucose and lipid metabolism and plays a key role in systemic immune activation 1-3. Hepatocytes coordinate triglyceride (TG) and cholesteryl ester (CE) storage and release with extrahepatic tissues by balancing lipogenesis, lipid droplet formation, and lipid uptake against lipid catabolism and excretion 2, 4, 5. Dysregulation of these processes, reflected by hypertriglyceridemia and hypercholesterolemia, contributes to liver diseases such as metabolic dysfunction-associated steatotic liver disease (MASLD; formerly non-alcoholic fatty liver disease) and metabolic dysfunction-associated steatohepatitis (MASH), and in advanced stages to fibrosis, cirrhosis, and hepatocellular carcinoma 6. Lipid-lowering drugs are the standard therapy for dyslipidemia and effectively reduce plasma TG and cholesterol levels 7, 8, but they have limited efficacy in treating fatty liver disease, underscoring its multifactorial nature 9. The most effective current therapy for MASLD is resmetirom, a recently FDA-approved thyroid hormone receptor β agonist 10. Additional drug classes under clinical evaluation include anti-diabetic drugs, peroxisome proliferator-activated receptor agonists, farnesoid X receptor agonists, thyroid hormone receptor agonists, fibroblast growth factor analogs, as well as anti-fibrotic and anti-inflammatory agents 9-12. Evidence for dietary supplements remains limited. Vitamin E, a lipophilic antioxidant, has shown some benefit 13, possibly by protecting against ferroptosis 14, 15 and dampening inflammation following hepatic ω-oxidation 16-19.

Resident macrophages (Kupffer cells) and monocyte-derived macrophages are abundantly present in the liver, where they play key roles in regeneration and immunological tolerance 20, 21. In the context of liver injury or metabolic and degenerative disease, these macrophages become activated and, under chronic inflammation, progressively dysfunctional 21, 22. Their shift from homeostatic, tissue-remodeling functions to a pro-inflammatory state characterized by cytokine release and lipid mediator production exacerbates liver injury and disease progression 21, 22. In addition, macrophages and other resident immune cells reprogram hepatocyte metabolism through regulatory circuits that are dysregulated in metabolic disease, thereby further driving pathology 23. Consequently, aberrant macrophage function has been linked to metabolic rewiring, and macrophage-targeted strategies are being explored for the treatment of acute and chronic liver disease 21.

Macrophage-derived lipid mediators, together with cytokines, orchestrate the biphasic inflammatory response, including resolution of inflammation and tissue regeneration 24. Their biosynthesis is initiated by phospholipase A_2_ (PLA_2_) isoenzymes, which release polyunsaturated fatty acids (PUFAs) from membrane phospholipids 25, 26. These PUFAs are subsequently converted by oxygenases, mainly cyclooxygenases (PTGS, COX), lipoxygenases (ALOX, LOX), and cytochrome P450 monooxygenases (CYP), often in cooperation with hydrolases, isomerases, and ligases, into structurally and functionally diverse classes of lipid mediators 27-31. The isoenzyme 5-lipoxygenase (ALOX5, 5-LOX) requires the membrane-associated 5-lipoxygenase-activating protein (ALOX5AP, FLAP), which channels newly released arachidonic acid (AA/20:4) to the translocated dioxygenase 32.

By binding to membrane or nuclear receptors, lipid mediators exert pro-inflammatory, anti-inflammatory, pro-resolving, or immunoregulatory functions, depending on spatial and temporal dynamics and the (patho)physiological context 26, 33, 34. Beyond inflammation, they also regulate metabolism 35-40, promote survival and proliferation, or facilitate cell death 41-43, among many other functions. For instance, ALOX5-derived leukotrienes (LTs) recruit and activate immune cells, enhance vascular permeability, and induce bronchoconstriction, thereby driving inflammation 28, 44-46, whereas CYP-derived epoxyeicosatrienoic acids inhibit inflammation and protect against cell death 47.

Liver diseases, including acute liver injury, MASH, fibrosis, cirrhosis, and hepatocellular carcinoma, are associated with the activation of distinct cell death programs that cause functional loss and tissue degradation 6, 48-51, while also propagating inflammation through the release of damage-associated molecular patterns (DAMPs) 6, 49. Growing evidence implicates ferroptosis in the pathogenesis of liver diseases, particularly MASH 50, 52, 53, although its molecular mechanisms and clinical relevance remain incompletely understood. Ferroptosis is a metabolically regulated form of cell death executed by iron-dependent peroxidation of PUFAs in membrane phospholipids, including phosphatidylethanolamines (PEs) 54-56. These oxidized phospholipids undergo truncation and disrupt membrane architecture through mechanisms that are still not fully resolved 57-59. Membrane peroxidation is triggered either by labile Fe(II), which generates hydroxyl radicals from hydrogen peroxide via the Fenton reaction 60, or enzymatically by iron-containing oxygenases, such as CYP enzymes 61, 62 or 15-lipoxygenase (ALOX15, 15-LOX) in complex with PE-binding protein 1 (PEBP1) 63, 64. A complex network of repair and protective systems, including glutathione peroxidase 4 (GPX4), multiple redox cycles, and Ca^2+^-independent PLA_2_, counteracts ferroptosis 65-67. Susceptibility to ferroptosis is strongly influenced by the membrane PUFA/monounsaturated fatty acid (MUFA) ratio, which in turn is regulated by metabolic drivers such as sterol regulatory element-binding protein 1 (SREBP-1) signaling, lipogenesis, lipid uptake, and phospholipid desaturation 68-71.

Although ferroptosis is immunogenic, it has also been reported to suppress specific immune cell populations 72-74. Its close link to lipid mediator production, however, is well established. For instance, (i) ALOX isoenzymes, particularly ALOX15, oxygenate both free fatty acids and membrane phospholipids 63, 64; (ii) PTGS2, a key enzyme in prostanoid biosynthesis 75, is upregulated during ferroptosis 70; and (iii) glutathione peroxidases detoxify (membrane) hydroperoxides 76, 77, while simultaneously reducing the redox tone required to activate the catalytic cycle of both PTGS and ALOX enzymes 77-79.

Liver diseases have metabolic, inflammatory, and degenerative/ferroptotic components and despite recent progress still lack effective therapies 6, 10, 50, 80, likely because single-target strategies are insufficient for such complex disorders. We therefore searched for multi-target natural products capable of modulating (neutral) lipid metabolism, protecting against ferroptosis, and exhibiting pronounced anti-inflammatory properties. This led us to the ent-trachylobane mitrephorone B (**2**), first isolated from *Mitrephora glabra* (Annonaceae) and previously reported to display moderate cytotoxicity against cancer cell lines as well as antimicrobial activity 81. Compound **2** inhibits membrane peroxidation and thereby preferentially protects against ferroptosis inducers over apoptosis, necroptosis, and pyroptosis inducers. Mechanistically, it alters neutral lipid content and fatty acid composition, including lipid saturation, but does not act as a lipophilic radical scavenger, does not affect labile iron availability or glutathione metabolism, and does not differentially regulate key ferroptosis-associated genes compared with a radical-trapping ferroptosis inhibitor used as control. Compound **2** protects against lipid overload-induced toxicity in immune cell-hepatocyte co-cultures *in vitro*, suppresses (pro-inflammatory) cytokine expression independently of nuclear factor kappa B (NF-κB) signaling, and inhibits LT biosynthesis by targeting ALOX5AP (dependent on the 9-oxo group). Context-dependently, it also reduces CE or TG levels in single or co-cultured hepatocyte and immune cell *in vitro* pre-disease and disease models. Small structural variations introduced via total synthesis allow fine-tuning of these activities, making **2** an attractive candidate lead structure for hepatoprotective drug development.

## Materials and Methods

### Small molecules, lipids and standards

Compounds **1**-**5** (purity ≥ 95% by ¹H-NMR) were synthesized as described 82, dissolved in DMSO, and stored in the dark at -80 °C (stock solutions) or -20 °C (working dilutions) under argon, minimizing freeze-thaw cycles. Inhibitors were dissolved in DMSO and stored at -20 °C. Lipid mediators, fatty acids, and standards were purchased from Cayman Chemical (Ann Arbor, MI) and stored at -80 °C. Aliquots were diluted in methanol/water (1:1) for fatty acids, oxylipins, and endocannabinoids, or in methanol for phospholipids, sphingolipids, neutral lipids, and free fatty acids, and kept at -80 °C under argon, protected from light.

### Culture of human HepaRG hepatocytes

Differentiated HepaRG cells (#HPR101, Biopredic International, Rennes, France; 3.1×10⁴ cells/cm²) were cultured in William's Medium E (Biochrom, Berlin, Germany) supplemented with 10% fetal calf serum (FCS; GIBCO, Darmstadt, Germany or Sigma-Aldrich, St. Louis, MO), 5 µg/mL human recombinant insulin (Lonza, Basel, Switzerland), 2 mM *L*-glutamine (GIBCO), 100 U/mL penicillin (GIBCO), 37 or 50 µM hydrocortisone 21-hemisuccinate (Cayman Chemical), and with or without 100 µg/mL streptomycin (GIBCO), at 37 °C and 5% CO_2_. Cells were regularly checked for mycoplasma contamination (Mycoplasma Alert plus; Lonza, #LT07-710) and their morphology monitored. For subculturing, cells were detached using trypsin/EDTA (GE Healthcare, Munich, Germany) before reaching confluence.

### Isolation of peripheral blood mononuclear cells (PBMCs)

Leukocyte reduction system chamber (LRSC) filters were obtained from male and female platelet donors (18-65 years) at the Central Institute for Blood Transfusion and Immunological Department of Tirol Kliniken GmbH (Austria). Platelet donors were physically examined by trained medical staff prior to donation. Buffy coats were obtained from female and male blood donors (18-65 years) at the Clinical Department of Blood Group Serology and Transfusion Medicine, Medical University of Graz (Austria). Blood donors were accepted based on a standardized health questionnaire and routine blood testing. All platelet and blood donors met the criteria of the Austrian Blood Donation Regulation (BGBI. II Nr. 217/2022) and provided informed consent for the use of their residual blood for scientific purposes.

Human PBMCs were freshly isolated from LRSC filters or buffy coats by dextran sedimentation followed by isopycnic density gradient centrifugation using Histopaque^®^-1077 (Sigma-Aldrich) at 400 × g for 20 min at room temperature 83, 84. The resulting PBMC fraction was further purified by hypotonic lysis of erythrocytes (water) and two consecutive washes with PBS pH 7.4 at 270 × g for 5 min at room temperature.

Studies on primary human innate immune cells were approved by the Ethical Committees of the Medical University Innsbruck (approval no. 1041/2020, June 19, 2020) and the University of Graz (approval no. 39/149/63 ex 2024/25, May 30, 2025).

### Monocyte-derived macrophages and polarization to M1 and M2 subtypes

Monocytes, the predominant cell type of PBMCs (46.4 ± 4.9% CD14⁺/CD16⁺ and CD14⁺/CD16⁻ cells as determined by flow cytometry), were cultured in RPMI 1640 medium (Sigma-Aldrich) supplemented with 10% FCS, 2 mM *L*-glutamine, 100 U/mL penicillin, 100 µg/mL streptomycin, and 20 ng/mL GM-CSF (HiSS Diagnostics GmbH, Freiburg, Germany) or 20 ng/mL M-CSF (HiSS Diagnostics GmbH) for 6 days to differentiate into GM-CSF- or M-CSF-derived macrophages. Differentiated macrophages were subsequently polarized for 48 h into M1-like macrophages by treatment with 100 ng/mL lipopolysaccharide (LPS; *Escherichia coli* O127:B8, Sigma-Aldrich) and 20 ng/mL interferon-γ (Peprotech, Hamburg, Germany), or into M2-like macrophages by treatment with 20 ng/mL interleukin (IL)-4 (Peprotech) 85.

### Treatment of PBMCs and macrophages for lipid mediator profiling

Human PBMCs (5×10⁶ cells/mL in 1 mL PBS pH 7.4, supplemented with 1 mM CaCl₂) were pre-incubated with vehicle (DMSO, 0.1%) or test compounds for 10 min at 37 °C, followed by stimulation with the Ca^2+^-ionophore A23187 (Cayman Chemical; 2.5 µM, 10 min) alone or in combination with AA/20:4 (Cayman Chemical; 20 µM).

M1 and M2 macrophages (2 mL PBS pH 7.4, plus 1 mM CaCl_2_) were pre-incubated with vehicle (DMSO, 0.1%) or test compounds for 15 min at 37 °C and 5% CO₂ prior to stimulation with *Staphylococcus aureus*-conditioned medium (SACM; 1%, 3 h). SACM was obtained by culturing *S. aureus* (LS1 strain) in brain heart infusion (BHI) medium for 18 h, followed by sterile filtration of the supernatant (3,400 × g, 10 min, room temperature) through a Rotilabo^®^ syringe filter (PVDF, 0.22 µm, Roth, Karlsruhe, Germany) 86.

Lipid mediator biosynthesis was terminated by addition of ice-cold methanol (2 mL for PBMCs, 3.5 mL for macrophages) containing deuterium-labeled internal standards: 200 pg d8-5(*S*)-HETE, d4-LTB_4_, d5-lipoxin (LX)A_4_, d5-resolvin (Rv)D2, d4-prostaglandin E_2_ (d4-PGE_2_), d11-(±)8(9)-epoxy-5*Z*,8*Z*,14*Z*-eicosatrienoic acid (d11-8,9-EET; for 48 h treatments only), and 2,000 pg d8-AA/20:4 (Cayman Chemical, Ann Arbor, MI).

### Determination of cell numbers, membrane integrity, and cellular dehydrogenase activity

Cell number and membrane integrity were determined after trypan blue staining using a Vi-CELL Series Cell Counter (Beckman Coulter, Krefeld, Germany; software: Vi-Cell XR Cell Viability Analyzer, version 2.06.3) 87.

Cellular metabolic activity was assessed via conversion of 3-(4,5-dimethylthiazol-2-yl)-2,5-diphenyltetrazolium bromide (MTT; Sigma Aldrich, St. Louis, MO) 88, 89. Briefly, HepaRG cells (1×10^4^/well in 100 µL routine culture medium) or human PBMCs (2×10^5^/well in 100 µL RPMI 1640 medium supplemented with 5% FCS, 2 mM *L*-glutamine, 100 U/mL penicillin, and 100 µg/mL streptomycin) were treated with vehicle (DMSO, 0.5%) or test compounds in the presence or absence of the ferroptosis inducer RSL3 (0.2 or 0.4 µM; Cayman Chemical) for 24 or 48 h at 37 °C and 5% CO_2_. The pan-kinase inhibitor staurosporine (1 µM, Sigma Aldrich) served as a cytotoxic reference. After incubation, MTT solution (5 mg/mL in PBS pH 7.4; 20 µL) was added and cells were incubated for 3 h. The resulting blue formazan crystals were solubilized with SDS lysis buffer (10% SDS in 20 mM HCl; 100 µL) for more than 16 h under orbital shaking (Grant Bio PSU-20i, Cambridge, United Kingdom; Wippschüttler RS-RS 6 digital, Phoenix, Garbsen, Germany). Absorbance was measured at 570 nm using a SpectraMax iD3 Microplate Reader (Molecular Devices, San José, CA) or a Hidex Sense microplate reader (Hidex, Turku, Finland).

### Assessment of apoptotic and necroptotic cell death in hepatocytes

HepaRG cells were plated in 96-well plates at 1×10^4^ cells per well in 100 µL of routine culture medium and maintained for 24 h at 37 °C under 5% CO_2_. To trigger apoptotic cell death, cells were exposed to staurosporine (0.05 µM, Sigma-Aldrich), with DMSO (0.5%) used as the vehicle control. Cells were co-treated with vehicle (DMSO, 0.5%), the pan-caspase inhibitor Q-VD-OPh (50 µM; Sigma-Aldrich), or mitrephorone **2** (1 µM). Necroptosis was induced by treatment with tumor necrosis factor-α (TNF-α, 10 ng/mL, PreproTech, Cranbury, NJ) in the presence of Q-VD-OPh (50 µM) or cells were treated with DMSO (0.5%) as vehicle control. Co-treatments included vehicle (DMSO, 0.5%), the necroptosis inhibitor necrostatin-1s (50 µM; Cayman Chemical), or mitrephorone **2** (1 µM). Metabolic activity was determined by MTT assay as described above**.**

### Assessment of pyroptotic cell death in LPS-primed hepatocytes

HepaRG cells were plated in 96-well plates at 1×10^4^ cells per well in 100 µL of routine culture medium and maintained for 24 h at 37 °C under 5% CO₂. For pyroptosis induction, cells were primed with LPS (1 µg/mL) for 4 h, followed by treatment with nigericin (10 μM, Cayman Chemical). Where indicated, cells were co-treated with vehicle (DMSO, 0.5%), the pyroptosis inhibitor MCC950 (sodium salt, 1 µM; AdipoGen Life Sciences, San Diego, CA), or mitrephorone **2** (1 µM). Cell death rate was monitored by live-cell imaging using an Incucyte^®^ system (Sartorius, Göttingen, Germany). Propidium iodide (MedChemExpress, Monmouth Junction, NJ) was added to the medium at a final concentration of 2.5 µM to detect loss of plasma membrane integrity as an indicator of cell death. Plates were placed into the Incucyte^®^ live-cell analysis system immediately after treatment. Phase-contrast and red fluorescence images were acquired at 3-h intervals throughout the incubation period using a label-free high definition phase imaging system. Red fluorescence was detected using an excitation filter of 525/40 nm and an emission filter of 635 nm (625-805 nm). Cell confluency was automatically determined from phase-contrast images using the Incucyte^®^ Live-Cell Analysis Software (Sartorius), and propidium iodide-positive cells were identified and quantified using the fluorescence channel-based analysis. The relative death rate was calculated by normalizing the number of propidium-iodide-positive objects, indicating dead cells, to cell confluence (%) for each time point and treatment.

### Lipid peroxidation analysis by BODIPY-C11 staining and flow cytometry

HepaRG cells were seeded into 12-well plates at a density of 2×10⁵ cells per well and maintained at 37 °C in a humidified incubator containing 5% CO_2_. Cells were treated with vehicle (DMSO, 0.25%), mitrephorone **2** (10 and 30 µM), or ferrostatin-1 (10 µM; Cayman Chemical) for 24 h and subsequently challenged with vehicle (DMSO, 0.25%) RSL3 (0.5 µM) to induce ferroptosis. After 2 h, C11-BODIPY (2 µM, Cayman Chemical) was added, and cells were incubated for an additional 30 min at 37 °C in the dark. Following staining, cells were washed with PBS pH 7.4, detached using 1× trypsin/EDTA, neutralized with growth medium, and collected together with all wash fractions. Samples were centrifuged at 1,200 × g for 5 min at 4 °C, washed twice with Hank's Balanced Salt Solution (HBSS) pH 7.4, and resuspended in the same buffer. Flow cytometric acquisition (λ_ex/em_ = 488/550 nm) was performed on a CytoFLEX system (Beckman, Austria), recording 10,000 events within a predefined gate for HepaRG cells. The same gating strategy was uniformly applied to all samples and is shown in **Figure S3A**. Data were analyzed using FlowJo v10.10.0 (FlowJo, Ashland, OR). Mean fluorescence intensity values were normalized to the vehicle control and expressed as percentage change.

### Lipid peroxidation analysis by 4-HNE immunofluorescence microscopy

HepaRG cells were seeded on sterile glass coverslips at a density of 2.5×10^5^ cells per well and incubated at 37 °C in a humidified atmosphere containing 5% CO_2_. Cells were treated for 24 h with vehicle (DMSO, 0.2%), mitrephorone **2** (10 µM), or ferrostatin-1 (10 µM). Ferroptosis was subsequently induced by exposure to RSL3 (0.5 µM). After 2 h, the culture medium was removed and cells were washed twice with PBS pH 7.4. Fixation was performed using 4% paraformaldehyde in PBS pH 7.4 for 15 min at room temperature, followed by three washes with PBS pH 7.4. Cells were then permeabilized with 0.05% Triton X-100 in PBS pH 7.4 for 10 min, washed three additional times with PBS pH 7.4, and blocked for 30 min at room temperature in PBS pH 7.4 containing 1% BSA. Samples were exposed for 1 h at room temperature to a primary mouse monoclonal anti-4-HNE antibody (clone 12F7; 1:100 dilution; SMC-511D-STR; StressMarq Biosciences, Victoria, Canada) in PBS pH 7.4 containing 0.1% BSA and 0.01% Tween 20. After washing, cells were incubated for 30 min at room temperature with an Alexa Fluor™ 555-conjugated goat anti-mouse IgG secondary antibody (1:750; A21422, Lot 2418520; Thermo Fisher Scientific). Samples were counterstained and mounted using ProLong™ Gold Antifade Mountant with DNA Stain DAPI (Thermo Fisher Scientific). Fluorescence images were acquired using a BZ-X800 fluorescence microscope (Keyence, Neu-Isenburg, Germany) equipped with a DAPI filter (OP-87762, λ_ex_ = 360 ± 20 nm, λ_em_ = 460 ± 25 nm) and TRITC filter (OP-87764, λ_ex_ = 545 ± 12.5 nm, λ_em_ = 605 ± 35 nm) along with the BZ-800 Viewer and Analyzer software. Images were capture using a Plan Apochromat 20× (BZ-PA20, NA 0.75) objective and a BZ-X800 camera, operated via the BZ-X800 Viewer software. Image acquisition parameters, including exposure times, were kept constant across all experimental conditions and biological replicates. Quantitative fluorescence analysis was performed using Fiji/ImageJ. Individual microscopic images are shown in the raw data file.

### Quantification of labile iron levels

HepaRG cells were seeded at a density of 2.5×10^5^ cells per well in 12-well plates in William's E medium without any supplements and serum to avoid potential interference from serum-derived iron. Cells were treated with compound **2** (10 µM) or vehicle (DMSO, 0.25%) for 24 h at 37 °C and 5% CO_2_. Ferroptosis was subsequently induced by the addition of imidazole ketone erastin (IKE, 10 µM, Cayman Chemical) for an additional 24 h. Control cells received the corresponding vehicle treatment (0.25 % DMSO). Cells were detached using trypsin/EDTA and collected by centrifugation (1150 × g, 5 min, 4 °C), washed once with HBSS pH 7.4, and stained with Ferro Orange (0.5 µM) for 30 min at 37 °C and 5% CO_2_. As a negative control, 2,2′-bipyridine (0.5 mM, Sigma-Aldrich) was pre-incubated for 30 min with the FerroOrange (Sigma-Aldrich) staining solution prepared in HBSS pH 7.4. After staining, cells were centrifuged again (1,150 × g, 5 min, 4 °C), the supernatant was removed, and the cell pellet was resuspended in HBSS pH 7.4. FerroOrange fluorescence was quantified by flow cytometry using a CytoFLEX flow cytometer (Beckman Coulter; excitation at 488 nm; emission collected using a 585/42 nm band-pass filter). The gating strategy is shown in **Figure S3B**.

### Measurement of intracellular GSH and GSSG

HepaRG cells were seeded in 12-well plates (3.6×10^5^ cells/well) and cultured for 24 h at 37 °C and 5% CO_2_ before treatment with vehicle (DMSO, 0.1%), IKE (10 µM), buthionine sulfoximine (BSO, 20 µM, Cayman Chemical), or mitrephorone **2** (10 µM) for an additional 24 h. After treatment, cells were washed with PBS pH 7.4, detached using trypsin/EDTA, and collected by centrifugation (400 × g, 5 min, 4 °C). The resulting cell pellets were washed twice with PBS pH 7.4 and taken up in ice-cold KPE buffer (0.1 M potassium phosphate buffer pH 8 with 0.01 M EDTA) containing protease inhibitors (10 µg/mL leupeptin, 60 µg/mL soybean trypsin inhibitor, 2.7 mM sodium vanadate, 2.5 mM sodium pyrophosphate, and 1 mM phenylmethanesulfonyl fluoride). Cell lysis was achieved by repeated syringe aspiration using a 25G needle (Zlloo/Amazon, Seattle, WA). Lysates were centrifuged (18,000 × g, 10 min, 4 °C), and protein concentrations in the supernatants were determined using the DC Protein Assay Kit (Bio-Rad Laboratories, Munich, Germany). Proteins in the supernatants were subsequently precipitated by adding 50% aqueous trichloroacetic acid (TCA, Carl Roth). Samples were vortexed, incubated on ice for 10 min, and centrifuged at 9,100 × g for 10 min at 4 °C. The resulting supernatants were directly used for the determination of reduced glutathione (GSH) levels. For glutathione disulfide (GSSG) quantification, supernatants were pre-incubated with *N*-ethylmaleimide (NEM, 8 mM, Sigma Aldrich) for 30 min at room temperature beforehand. Samples, blank (KPE buffer), or standards were diluted with KPE or 0.1 N NaOH for GSH or GSSG measurements, respectively. Subsequently, *o*-phthalaldehyde (1 mg/mL in methanol; Sigma-Aldrich) was added, and samples were incubated for 15 min. Fluorescence was measured at λ_ex/em_ = 355/460 nm using a Hidex Sense microplate reader (Hidex, Finland). Corresponding blank values (KPE buffer, *o*-phthalaldehyde, and NaOH) were subtracted prior to data analysis.

### Analysis of phospholipid peroxidation in artificial membranes

Unilaminar liposomes composed of egg phosphatidylcholine (PC, Sigma-Aldrich) were prepared following an established protocol 90-92. PC was initially dissolved in chloroform, and the solvent was removed under a continuous stream of argon to form a uniform lipid film. To ensure complete solvent evaporation, the vial was maintained under argon for an additional 30 min. The lipid film was subsequently rehydrated with PBS pH 7.4 to a final lipid concentration of 20 mM. The resulting suspension underwent ten freeze-thaw cycles consisting of alternating incubation on ice (4 min) and at room temperature (4 min). Each cycle was followed by sonication for 4 min in an ultrasonic water bath (Sonorex Super Rx 2554, Germany). Liposomes were then extruded 20-25 times through a 100 nm polycarbonate membrane using a mini-extruder (Avanti Research, Alabaster, AL). The mean particle diameter and polydispersity index (PDI) were determined by dynamic light scattering (Zetasizer Advance Ultra, Malvern Panalytical, Almelo, Netherlands). Only liposome preparations with an average diameter of approximately 100 nm and a PDI between 0.2 and 0.3 were used for subsequent experiments. Liposome suspensions were stored at 4 °C and used within two weeks. Phospholipid peroxidation was quantified using a modified fluorescence-enhanced inhibited autoxidation (FENIX) assay. Experiments were performed in black 96-well polypropylene plates (96-Well Optical Bottom Plate, polymer base with lid; Thermo Fisher Scientific). Liposomes (15 µL, 20 mM in PBS pH 7.4) were mixed with 1 µL STY-BODIPY (300 µM in PBS pH 7.4; Cayman Chemical) and PBS pH 7.4 to obtain a final volume of 285 µL per well. Vehicle (DMSO, 0.67%), compound **2** (10 µM), and/or liproxstatin-1 (10 µM, Cayman Chemical) were added to yield the indicated final concentrations, and plates were incubated at 37 °C for 10 min with gentle agitation using a CLARIOstar microplate reader (BMG Labtech, Ortenberg, Germany). Autooxidation was initiated by the addition of 3 µL of the radical initiator DTUN (2 µM; Cayman Chemical), and the volume was adjusted with PBS pH 7.4 to 300 µL, yielding final concentrations of 1 mM liposomes, 1 µM STY-BODIPY, and 2 µM DTUN. After 5 min of vigorous mixing and a 10 min equilibration at 37 °C, fluorescence was measured using a Clariostar microplate reader (BMG Labtech) in bottom-read mode at excitation/emission wavelengths of 488/528 nm at 5 min intervals for up to 10-18 h.

### Solid phase extraction and UPLC-MS/MS analysis of lipid mediators

Samples from cell-based lipid mediator studies were stopped with methanol as described above. Aliquots (PBMCs: 3 mL; macrophages: 5.5 mL) were stored at -20 °C for at least 1 h to allow protein precipitation. After centrifugation (750 × g, 10 min, 4 °C), the supernatants were mixed with acidified water (pH 3.5; 8 mL for PBMCs, 9 mL for M1/M2 macrophages) and applied to solid-phase extraction cartridges (Sep-Pak^®^ Vac 6cc, 500 mg/6 mL C18, Waters, Milford, MA), which were conditioned with 6 mL methanol and equilibrated with 2 mL water. Cartridges were sequentially washed with 6 mL water and 6 mL *n*-hexane (4 °C). Free fatty acids and lipid mediators were eluted with 6 mL methyl formate. The eluates were evaporated to dryness using a TurboVap LV (Biotage, Uppsala, Sweden) and redissolved in methanol/water (1:1). Samples were centrifuged three times (750 × g, 10 min, 4 °C; then twice at 21,100 × g, 5 min, 4 °C) before UPLC-MS/MS analysis 84, 85.

Oxylipins were chromatographically separated on an Acquity UPLC BEH C18 column (130 Å, 1.7 µm, 2.1 × 100 mm; Waters, Milford, MA) at 55 °C using an ExionLC AD UHPLC system (Sciex, Framingham, MA). Mobile phase A consisted of methanol with 0.01% acetic acid, and mobile phase B of water/methanol (90/10) with 0.01% acetic acid. Separation was achieved with a linear gradient from 35.6% to 84.4% A over 12.5 min, followed by 5 min of isocratic elution at 97.8% A 169. For the analysis of lipid mediator profiles 48 h after treatment with mitrephorones and for **Figure 5B**-**C**, an alternative gradient was applied, ramping from 35.6% to 84.4% A within 12.5 min, further increasing to 87.0% A over 2.5 min, and holding for 3 min at 97.8% A 83, 84.

Lipid mediators were analyzed by scheduled multiple reaction monitoring (MRM) using default detection windows of 90 s (negative ion mode) and 60 s (positive ion mode) on a QTRAP 6500^+^ mass spectrometer (Sciex) equipped with an IonDrive Turbo V source and a TurbolonSpray probe for electrospray ionization (Sciex, Framingham, MA) under polarity switching. Negative ion mode was employed to detect oxylipins and PUFAs, whereas endocannabinoids were analyzed in positive ion mode. Curtain, sheath, and auxiliary gas pressures were set to 40 psi, collision gas to medium, heated capillary temperature to 500 °C, and ion spray voltage to -4000 V and 4000 V for negative and positive modes, respectively. MRM transitions used for quantitation are listed in **Table 1**.

For **Figure 5C**, LTB_4_ (*m/z* 335.2228 → *m/z* 195.1032) and the internal standard LTB_4_-d_4_ (Cayman Chemical; *m/z* 339.2479 → *m/z* 197.1146) were quantified by MRM^HR^ in negative ion mode using a ZenoTOF 7600 mass spectrometer (Sciex) equipped with an OptiFlow Turbo V electrospray ionization source (Sciex). Instrument control and data processing were performed using Sciex OS software version 4.0 (Sciex). Mass spectrometric source parameters were set as follows: ion source gas 1 (nebulizer gas), 60 psi; ion source gas 2 (heating gas), 60 psi; curtain gas, 55 psi; heated capillary temperature, 575 °C; ion spray voltage, -4,400 V. TOF-MS1 data were acquired over a mass range of *m/z* 60-650 with an accumulation time of 0.1 s, a declustering potential of -100 V, and a collision energy of 10 eV. Scheduled TOF-MS2 data acquisition by MRM^HR^ covered a mass range of m/z 60-400 with an accumulation time of 0.01 s, a Zeno threshold of 80,000 counts s^-1^, a declustering potential of -50 V (LTB_4_) and -70 V (LTB_4_-d_4_), and a collision energy of -21 eV for both analytes. The instrument was automatically calibrated every five injections using ESI Negative Calibration Solution (#5042913) for the SCIEX X500 System (Sciex).

Lipid amounts were calculated from 10-11-point standard curves and normalized to a subclass-specific deuterated internal standard and cell number. Mass spectra were acquired and processed using Analyst software versions 1.6.3, 1.7.1, or 1.7.2 or Sciex OS 4.0 (Sciex).

Recent studies on di- and trihydroxylated oxylipins indicate that certain isoforms are not easily resolved on non-chiral stationary phases 93. Accordingly, these analytes are reported here as specialized pro-resolving mediators (SPMs) and/or isomers (SPM/iso). Obvious isomers distinguishable by MS/MS fragmentation or retention time were excluded from the analysis.

### Extraction and quantitative lipidomic analysis of phospholipids and neutral lipids

Phospholipids, neutral lipids, sphingolipids, and free fatty acids were extracted from cell pellets by successive addition of PBS pH 7.4, methanol, chloroform, and saline in a final ratio of 14:34:35:17 87, 94. The lower organic phase was evaporated to dryness using a Concentrator Plus System (Eppendorf, Hamburg, Germany; high vapor pressure mode), and the resulting lipid film was dissolved in methanol, centrifuged twice (21,100 × g, 4 °C, 5 min), and subjected to UPLC-MS/MS analysis. Internal standards (obtained from Sigma-Aldrich) were as follows: 1) PBMCs (48 h) - 1,2-dimyristoyl-*sn*-glycero-3-phosphatidylcholine, 1,2-dimyristoyl-*sn*-glycero-3-phosphatidylethanolamine, 1,2-dimyristoyl-*sn*-glycero-3-phosphatidylglycerol, 1,2-myristoyl-*sn*-glycero-3-phosphoserine, 1,2-dioctanoyl-*sn*-glycero-3-phospho-(1'-myo-inositol), (15,15,16,16,17,17,18,18,18-d9)oleic acid, cholest-5-en-3ß-yl-heptadecanoate, 1,2,3-tritetradecanoyl-*sn*-glycerol, 1,2-dimyristoyl-*sn*-glycerol, 1′,3′-bis[1,2-dimyristoyl-*sn*-glycero-3-phospho]-glycerol, *N*-heptadecanoyl-*D*-erythro-sphingosine, *N*-heptadecanoyl-*D*-erythro-sphingosylphosphorylcholine. 2) HepaRG/PBMC co-culture - 1,3-dipentadecanoyl-2-oleoyl(d7)-glycerol and 25,26,26,26,27,27,27-heptadeuteriocholest-5-en-3β-ol (9*Z*-octadecenoate).

Chromatographic separation of PC, PE, phosphatidylinositol (PI), phosphatidylglycerol (PG), phosphatidylserine (PS), TG, diacylglycerol (DG), CE, cardiolipin (CL), sphingosine (So), sphinganine (Sa), (dihydro)ceramide ((dh)Cer), hexosylceramide (HexCer), ceramide-1-phosphate (C1P), and (dihydro)sphingomyelin [(dh)SM] species, as well as free fatty acids was performed on an Acquity UPLC BEH C8 column (130 Å, 1.7 μm, 2.1×100 mm, Waters) using an ExionLC AD UHPLC system (Sciex) 87, 95. Separation of these lipids was conducted at 45 °C with a flow rate of 0.75 mL/min, using acetonitrile/water (95:5) with 2 mM ammonium acetate as mobile phase A and water/acetonitrile (90:10) with 2 mM ammonium acetate as mobile phase B. In variation to these settings, PS species were separated at 65 °C with a flow rate of 0.85 mL/min. For TG, DG, and CE analysis, 100% isopropanol was used instead as mobile phase B, and CL analysis was performed at 55 °C with a flow rate of 0.60 mL/min using methanol (2 mM ammonium acetate) as mobile phase A and water (2 mM ammonium acetate) as mobile phase B. To separate PC, PE, PI, PG, PS, and free fatty acids, the gradient was increased linearly from 75% to 85% A over 5 min and to 100% A over the next 2 min, followed by 2 min of isocratic elution. The same gradient was applied for sphingolipids, with the isocratic phase extended to 13 min. TG, DG, and CE were separated using an initial A/B ratio of 90/10, which was ramped to 70/30 over 6 min, and held for 4 min 87. For **Figure S8A**, the gradient alternatively started at 100% mobile phase A, linearly increased to an A/B ratio of 70/30 over 9 min, and maintained isocratically for 3 min. CL were eluted by increasing mobile phase A from 85% to 98% over 8 min, followed by 1 min of isocratic elution.

Lipids were detected using a QTRAP 6500^+^ mass spectrometer (Sciex) with an IonDrive Turbo V Source and TurbolonSpray probe. Mass spectrometric parameters are listed in **Table 2A** and** B**. PC ([M+OAc]^-^ to fatty acid anions) 96, other glycerophospholipids ([M-H]^-^ to fatty acid anions) 96, and CL ([M-2H]²⁻ to fatty acid anions) were quantified by MRM in negative ion mode 87. Free fatty acids were analyzed by single reaction monitoring in negative ion mode 87. TG, DG, and CE were quantified by MRM from [M+NH_4_]^+^ to [M - fatty acid anion]^+^ in positive ion mode 87, 97. Sphingolipids were quantified using transitions from [M+H]^+^ to [M+H-H_2_O]^+^ (So, Sa, dhCer), *m/z* 184.1 ([dh]SM), and *m/z* 264.4 (Cer, HexCer, C1P) 83, 84.

### Determination of human recombinant 5-lipoxygenase activity

Human recombinant ALOX5 enzyme (Cayman Chemical, 10 U) was preincubated in PBS pH 7.4 containing 1 mM EDTA and 1 mM ATP (1 mL) with vehicle (DMSO, 0.1%) or test compounds for 10 min on ice. AA/20:4 (20 µM; Cayman Chemical) and CaCl_2_ (2 mM) were then added, and samples were incubated at 37 °C for 10 min 85, 89. The enzymatic reaction was stopped with ice-cold methanol (1 mL), and PGB_1_ (200 ng; Cayman Chemical) was added as an internal standard. Samples were acidified with 530 µL PBS plus 60 mM HCl, centrifuged (750 × g, 10 min, 4 °C), and the supernatants were loaded onto Clean-Up C-18 Endcapped SPE cartridges (100 mg, 10 mL, UCT, Bristol, PA), conditioned with methanol (1 mL, twice) and equilibrated with water (1 mL). Cartridges were washed with water (1 mL) and methanol/water (75/25, 1 mL), and lipid mediators were eluted with 100% methanol (300 µL). Eluates were diluted in 120 µL water, centrifuged (21,100 × g, 10 min, 4 °C), and analyzed by UPLC-PDA 85, 89.

LTB_4_ isomers and 5-HETE were separated on a Kinetex C-18 LC column (100 Å, 1.3 μm, 2.1×50 mm, Phenomenex, Torrance, CA) at a flow rate of 0.45 mL/min using a Nexera X2 UHPLC system (Shimadzu, Kyoto, Japan) operated at 40 °C. The gradient used solvent A (50% methanol/50% water/0.05% trifluoroacetic acid) and solvent B (100% methanol/0.05% trifluoroacetic acid), starting at 14% B and 86% A. After 2 min of isocratic elution, the gradient was increased stepwise to 46% B over 2 min, then to 90% B over another 2 min. LTB_4_ isomers and PGB_1_ were detected at 280 nm, and 5-HETE at 235 nm using a photodiode array detector (SPD-M20A, Shimadzu). Chromatograms were acquired and processed using LabSolutions (version 5.97, Shimadzu), and lipid mediator amounts were calculated using analyte-specific extinction coefficients with PGB_1_ as an internal standard 85, 89.

### Assessment of ALOX5AP-dependent LTB_4_ formation

Human embryonic kidney (HEK)-293 cells (#CRL-1573, ATCC) were maintained in high-glucose DMEM (4.5 g/L; Thermo Fisher Scientific) containing 10% FCS, 100 U/mL penicillin, and 100 µg/mL streptomycin at 37 °C in a humidified atmosphere containing 5% CO_2_. Cells were routinely tested for mycoplasma contamination, monitored for characteristic morphology, and passaged using trypsin/EDTA before reaching confluence.

For transfection, HEK-293 cells were seeded at 4×10^5^ cells per well in 6-well plates and transfected 24 h later with an empty control vector (pRP[EXP]-EGFP-CAG>hypBase), a human ALOX5 expression vector (pPB[EXP]-CMV>ALOX5[NM_000698.5]), and/or a human ALOX5AP expression vector (pPB[EXP]-CMV>hALOX5AP[NM_001204406.2]). Vector DNA (1 µg) in 250 µL Opti-MEM (Thermo Fisher Scientific) was combined with 3 µL TurboFectin 8.0 (OriGene, Rockville, MD), incubated 15 min at room temperature, and added dropwise to cells in 1 mL culture medium. After 48 h, the cells were washed and preincubated in 1.5 mL PBS pH 7.4 containing 1 mM CaCl_2_ in the presence of vehicle (DMSO, 0.1%) or **2** (10 µM) for 15 min. LTB_4_ formation was initiated by addition of A23187 (2.5 µM) and AA/20:4 (2 µM) and terminated by the addition of 2.5 mL ice-cold methanol containing 200 pg d4-LTB_4_ as internal standard.

### Analysis of cytokine expression

Human PBMCs (1.4×10^6^ cells/mL in 1 mL RPMI 1640 supplemented with 5% FCS, 2 mM *L*-glutamine, 100 U/mL penicillin, and 100 µg/mL streptomycin) were preincubated with vehicle (DMSO, 0.1%) or test compounds for 30 min and then stimulated with LPS at 10 ng/mL for 4 h (TNF-α, IL-8) or 18 h (IL-1β, IL-1 receptor antagonist (IL-1ra), IL-6, monocyte chemoattractant protein-1 (MCP-1), IL-10, IL-12 (p70), IL-23). Supernatants were collected, centrifuged (21,000 × g, 5 min, 4 °C), and cytokine levels were measured immunologically. TNF-α, IL-1β, IL-6, IL-8, MCP-1, and IL-10 were detected using in-house ELISA systems based on DuoSet ELISA Development Kits (Bio-Techne, Minneapolis, MN) according to the manufacturer's instructions. IL-1ra, IL-12 (p70), and IL-23 were quantified using a Bio-Plex 200 System with Bio-Plex Pro Human Cytokine Singleplex Sets, Bio-Plex Pro Reagent Kit III with Flat Bottom Plate, and Bio-Plex Pro Human Cytokine Screening Panel Standards (BIO-RAD, Hercules, CA). Cytokine and chemokine concentrations were calculated from an 8-point standard curve 83.

### RNA isolation, cDNA synthesis, and qPCR

HepaRG cells were seeded at a density of 1×10⁶ cells per well in 6-well plates and incubated for 24 h at 37 °C in a humidified atmosphere containing 5% CO_2_. The cells were then treated with vehicle control (DMSO, 0.1%), RSL3 (0.5 µM), RSL3 in combination with mitropherone **2** (10 µM), or RSL3 in combination with ferrostatin-1 (10 µM) for 48 h. After treatment, total RNA was extracted using the innuPREP RNA Mini kit (#845-KS-2040250, IST Innuscreen, Berlin, Germany) according to the manufacturer's instructions. The RNA concentrations were adjusted to equal levels, and cDNA was generated using qScript reverse transcriptase (#95047-100, Quantabio, Beverly, MA). Quantitative real-time polymerase chain reaction (qPCR) was performed using 0.6 µL of the cDNA template (final concentration: 1.5 ng/µL), gene-specific forward and reverse primers (0.5 µM each; Eurofins Genomics AT, Vienna, Austria, **Table S1**), and innuMIX qPCR DSGreen standard according to the manufacturer's instructions (#845-AS-1320500, IST InnuScreen, Berlin, Germany). Reactions were run in 0.2 ml Multiply-µStrip PCR tubes (Sarstedt) on a QuantStudio 3 real-time PCR System (Thermo Fisher Scientific). The thermal cycling conditions consisted of an initial denaturation at 95 °C for 2 min, followed by 40 amplification cycles of denaturation at 95 °C for 20 s and annealing/extension at 60 °C for 45 s. This was followed by a melt curve analysis comprising a step at 95 °C for 15 s, incubation at 60 °C for 1 min, and a final step at 95 °C for 30 s. The QuantStudio^TM^ Design & Analysis Software v1.5.1 (Thermo Fisher Scientific) was used for amplification and data acquisition. Transcript levels were quantified using the standard 2^-ΔΔCT method. All samples and standards were analyzed in technical duplicates. Gene expression levels were normalized to the mean of the reference gene GAPDH.

### SDS-PAGE and Western Blotting

For the analysis of NF-κB inhibitor α (NFKBIA, IκBα) expression and phosphorylation, freshly isolated PBMCs (3.84×10^6^ cells in RPMI 1640 supplemented with 2 mM *L*-glutamine, 100 U/mL penicillin, and 100 µg/mL streptomycin) were serum-starved overnight. Cells were pre-incubated for 30 min with vehicle (DMSO, 0.1%) or test compounds in the presence of 2% FCS, then stimulated with LPS at 10 ng/mL for 15 min or 1 h. Samples were placed on ice, washed twice with ice-cold PBS pH 7.4, and lysed by sonication (2 × 5 s on ice, 35% of 125 W; Q125 Sonicator, QSonica, Newtown, CT) in 100 µL lysis buffer containing 20 mM Tris-HCl pH 7.4, 150 mM NaCl, 2 mM EDTA, 1% Triton X-100, 5 mM sodium fluoride, 10 µg/mL leupeptin, 60 µg/mL soybean trypsin inhibitor, 2.7 mM sodium vanadate, 2.5 mM sodium pyrophosphate, and 1 mM phenylmethanesulfonyl fluoride.

To determine the expression of ALOX5, MAP2K1/2, and MAP2K1/2 phosphorylation, PBMCs (1×10^7^ cells) were pre-incubated with vehicle (DMSO, 0.1%) or test compounds for 48 h in RPMI 1640 medium supplemented with 5% FCS, 2 mM *L*-glutamine, 100 U/mL penicillin, and 100 µg/mL streptomycin. Cells were harvested by sequential rinsing of the wells with ice-cold PBS pH 7.4 containing 5 mM EDTA (1 mL) and PBS pH 7.4 (1 mL). After two additional washes with ice-cold PBS pH 7.4 (1 mL each; 270 × g, 7 min, 4 °C and 2000 × g, 7 min, 4 °C), cell pellets were resuspended in 100 µL lysis buffer and sonicated (2 × 5 s on ice, 35% of 125 W; Q125 Sonicator, QSonica, Newtown, CT).

To assess the cleavage of gasdermin D as a marker of pyroptosis, HepaRG cells were seeded at a density of 1×10⁶ cells per well in a 6-well plate and incubated for 48 h at 37 °C in a humidified atmosphere containing 5% CO_2_. Cells were then left untreated in routine culture medium or primed with LPS (1 μg/ml) for 4 h. Following LPS priming, cells were co-treated for 1 h with either vehicle (DMSO, 0.1%), the NLRP3 inhibitor MCC950 (1 µM), or mitrephorone **2** (1 µM). Subsequently, the cells were treated with vehicle (ethanol, 0.1%) or stimulated with nigericin (10 µM) for an additional 2 h to induce inflammasome activation.

To determine the phosphorylation of receptor-interacting serine/threonine kinase 1 (RIPK1) during necroptosis induction, HepaRG cells were seeded at a density of 1×10⁶ cells per well in a 6-well plate and incubated for 48 h at 37 °C in a humidified atmosphere containing 5% CO_2_. Cells were pretreated in routine culture medium with vehicle (DMSO, 0.1%) or the pan-caspase inhibitor Z-VAD-FMK (20 µM) for 30 min and subsequently treated with vehicle (DMSO, 0.1%) or human TNF-α (20 ng/mL) in combination with the SMAC mimetic SM-164 (100 nM) to induce necroptosis. For control and inhibition experiments, cells treated with human TNF-α/Z-VAD-FMK/SM-164 were co-treated with either vehicle (DMSO, 0.1%), the RIPK1 inhibitor necrostatin-1 (50 µM, Cayman Chemical), or mitrephorone **2** (1 µM).

Lysates were centrifuged (12,000 × g, 10 min, 4 °C), and protein concentrations were determined using a DC Protein Assay Kit (Bio-Rad Laboratories). Equal amounts of total protein were mixed with 5× SDS/PAGE loading buffer (125 mM Tris-HCl, pH 6.5; 25% sucrose; 5% SDS; 0.25% bromophenol blue; 5% β-mercaptoethanol), heated at 95 °C for 5 min, and 6-30 µg of protein per lane were separated by 10% SDS-PAGE. Proteins were transferred onto 0.45 µm nitrocellulose membranes (Cytiva Amersham™ Protran™, GE Healthcare, Munich, Germany) and blocked with 5% BSA (Roth) or 5% skim milk (Sigma-Aldrich) in PBS pH 7.4 for 1 h at room temperature. Membranes were incubated overnight at 4 °C with primary antibodies: mouse monoclonal anti-NFKBIA (1:800; #4814S; Cell Signaling, Danvers, MA), rabbit monoclonal anti-phospho-NFKBIA (1:1000; #2859; Cell Signaling), mouse anti-ALOX5 (1:1000; #610695; BD Biosciences), rabbit anti-MAP2K1/2 (1:1000; #9122; Cell Signaling), rabbit anti-phospho-MAP2K1/2 (1:1000; #9121; Cell Signaling), rabbit anti-cleaved gasdermin D (Asp275) (1:500, E7H9G, #36425T; Cell Signaling), rabbit anti-phospho-RIPK1 (Ser166) (1:500, D1L3S, #65746; Cell Signaling, Danvers, MA), rabbit anti-RIPK1 (1:500, D94C12, #3493; Cell Signaling), rabbit monoclonal anti-β-actin (1:1000; #4970; Cell Signaling), or mouse monoclonal anti-β-actin (1:1000; #3700; Cell Signaling). Following three washes with TBS pH 7.4 containing 1% Tween-20 (8 min each), membranes were incubated for 1 h at room temperature with the corresponding secondary antibodies: DyLight^®^ 800 anti-mouse IgG (1:10,000; #SA5-10176; Thermo Fisher Scientific), DyLight^®^ 800 anti-rabbit IgG (1:10000; #SA5-10036 and #SA5-10044; Thermo Fisher Scientific), DyLight^®^ 680 anti-mouse IgG (1:10,000; #35519; Thermo Fisher Scientific), or DyLight^®^ 680 anti-rabbit IgG (1:10,000; #35569; Thermo Fisher Scientific).

For **Figure 2C** and **Figure 7C**, fluorescence signals of immunoreactive bands were detected using a Fusion FX7 Edge Imaging System (spectra light capsules: C680, C780; emission filters: F-750, F-850; VILBER Lourmat, Collegien, France). Densitometric analysis was performed with Bio-1D imaging software (version 15.08c, VILBER Lourmat) using rolling ball background subtraction. For **Figure 3F** and** Figure 4F, I**, the fluorescence of immunoreactive bands was visualized using a ChemiDoc MP Imaging system (BioRad, Hercules, CA) with epi-far red (excitation: 650-675 nm) or epi-near IR (excitation: 755-777 nm) and emission filters of 715/30 and 835/50. Images were processed with ImageLab software (BioRad) using local background subtraction. Protein levels were normalized to β-actin or GAPDH, and phospho-protein levels were normalized to their respective total protein levels. Uncropped blots are presented in the raw image file.

### OxLDL-induced conversion of monocyte-derived macrophages to foam cells

Human primary monocyte-derived macrophages (2.5×10^5^ M_GM-CSF_ per well of a 96-well plate) were serum-starved overnight in RPMI 1640 medium supplemented with 2 mmol/L *L*-glutamine, 100 U/mL penicillin, and 100 µg/mL streptomycin (100 µL) at 37 °C and 5% CO₂. The culture medium containing non-adherent cells was discarded, and cells were incubated in fresh medium supplemented with 2% FCS. Cells were preincubated with vehicle (DMSO, 1%) or test compounds for 1 h (37 °C, 5% CO_2_) and subsequently treated with oxLDL (50 µg/mL, Fischer Scientific) or left untreated for 48 h. After fixation (4% paraformaldehyde in PBS pH 7.4; 50 µL, 40 min), cells were washed with 60% aqueous isopropanol (60 µL, 5 min) and stained with Oil-Red-O (0.3% in 60% aqueous isopropanol; 50 µL, 25 min; Sigma Aldrich) to visualize intracellular lipid accumulation. Phase-contrast images were captured using a Motic AE31E Trinocular 100W microscope equipped with a Moticam 10+ camera and Motic Images Plus 3.0 ML Software (Motic China Group Co., Ltd.). For **Figure S8C**, phase-contrast images were acquired using a BZ-X800 fluorescence microscope (Keyence) equipped with a Plan-Apochromat 10× objective and a BZ-X800 camera, operated with BZ-X800 Viewer software. Following extensive washing (5 × 100 µL water), Oil-Red-O was extracted with 60% aqueous isopropanol (50 µL, 3 min) 98, and absorbance was measured at 345 nm using a SpectraMax iD3 Microplate Reader (Molecular Devices) or at 510 nm using a Hidex Sense microplate reader (Hidex) 99.

### Hepatocyte and PBMC co-culture models reflecting lipid overload, lipotoxicity, or (peroxidative) membrane stress

HepaRG cells (1×10^4^/well in 75 µL medium for 96-well plates or 1×10^6^/well in 1.5 mL medium for 6-well plates) were seeded in William's medium E supplemented with 10% FCS, 2 mM *L*-glutamine, 5 µg/mL human recombinant insulin (Lonza), 100 U/mL penicillin, and 100 µg/mL streptomycin. After 5-7 h at 37 °C and 5% CO₂, an equal number of freshly isolated PBMCs (25 µL for 96-well plates, 0.5 mL for 6-well plates) were added, and co-cultures were incubated overnight. For fatty acid supplementation, palmitic acid (PA)/16:0 and oleic acid (OA)/18:1 were first complexed with 5% BSA in culture medium by sonication and heating. Cells were then challenged with either: i) a balanced mixture of PA/16:0 and OA/18:1 (1 mM; 1:2) to induce lipid overload, ii) excess PA/16:0 (200 µM) to trigger lipotoxicity, or iii) AA/20:4 (20, 200, or 1000 µM) to increase susceptibility of cellular membranes to peroxidative stress. Metabolic stress was applied for 24 h before addition of vehicle (DMSO, 1% for 96-well plates, 0.1% for 6-well plates), compound **2** (10 µM), metformin (1 mM, Cayman Chemical), simvastatin (1 µM, Cayman Chemical), or pioglitazone (100 µM, Sigma-Aldrich)**,** followed by a further 24 h incubation. Cellular metabolic activity and intracellular lipid accumulation were determined in 96-well plates by MTT assay and Oil-Red-O staining, respectively (as detailed above). TG and CE levels, IL-1β expression, and membrane integrity were assessed by quantitative lipidomics, ELISA, and trypan blue staining, respectively, as described above. Phase-contrast images were captured using a Motic AE31E Trinocular 100W microscope equipped with a Moticam 10+ camera and Motic Images Plus 3.0 ML Software (Motic China Group Co., Ltd.).

### Co-regulated lipid networks and their correlation with ferroptosis sensitivity

Pearson correlation coefficients were calculated from the mean percentage changes in absolute or relative abundances of phospholipids, sphingolipids, neutral lipids, or free fatty acids (1 and 10 µM). Correlation-based networks were generated using the MetScape 3.1.3 plugin for Cytoscape 3.10.1 (Cytoscape Consortium) 100-102. Nodes represent individual lipid species and were connected by edges when positively correlated (*r* ≥ 0.7). Data were visualized using the Edge-weighted Spring Embedded Layout (correlation from matrix algorithm). Correlations between the efficiency to protect against RSL3-induced ferroptotic cell death and changes in absolute or relative lipid composition were calculated in Microsoft Excel Version 2302 (Microsoft 365 Apps for Enterprise; Microsoft) using Pearson correlation.

### Statistics

Data are presented as mean, mean ± SEM and individual values or single data from *n* independent experiments. Normal distribution was assessed using Shapiro-Wilk tests, and outliers were identified with Grubb's test for **Figure 5C** or for datasets with *n* ≥ 4 in all other figures (GraphPad Software, San Diego, CA). Sample size was not predetermined by a statistical method, and samples were not blinded. Data analysis was performed using Microsoft Excel (Version 2302, Microsoft 365 Apps for Enterprise, Redmond, WA). Non-transformed or log-transformed data were analyzed using two-tailed Student's *t* tests for pairwise comparisons (paired or unpaired; α = 0.05), ordinary or repeated measures one-way or two-way ANOVAs, or mixed-effects models (REML) ANOVAs for independent or correlated samples, followed by Dunnett's or Tukey's *post hoc* tests. Negative log10(*P* values) in volcano plots were calculated from two-tailed multiple paired Student's *t* tests. Statistical analyses were conducted with GraphPad Prism 9 or 10 (GraphPad Software), and *P* values < 0.05 were considered statistically significant.

### Generative AI and AI-assisted technologies

During the preparation of this work the authors used ChatGPT 4.0 and 5.0 and DeepL Write in order to improve readability and language of all manuscript sections. After using these tools, the authors reviewed and edited the content as needed and take full responsibility for the content of the publication.

## Results

### Mitrephorones combine anti-ferroptotic and anti-inflammatory activities

**Figure 1:**
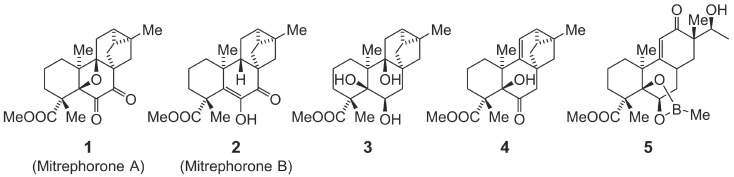
Structures of the natural products 1 and 2 and their derivatives 3 to 5.

**Figure 2:**
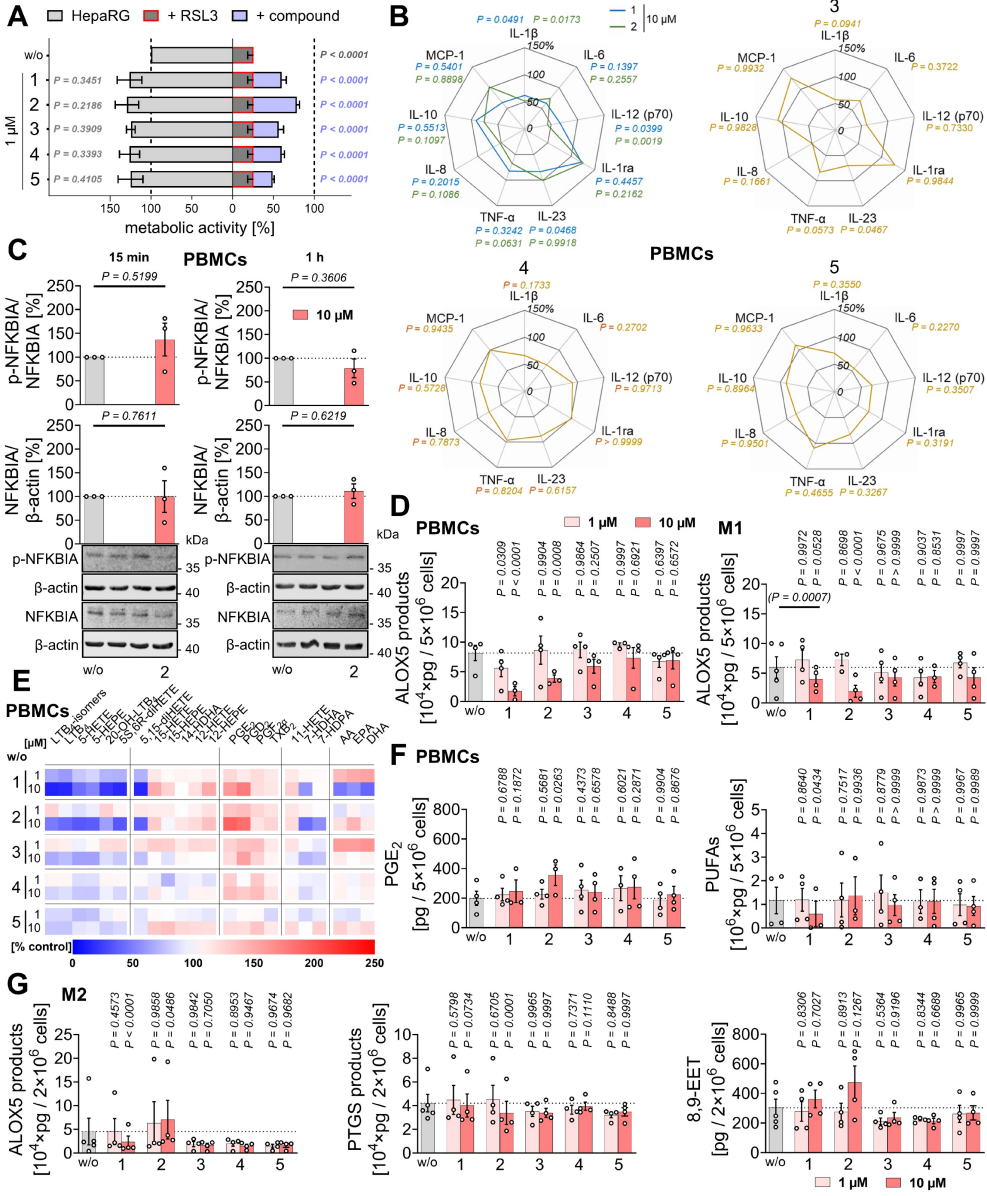
**Mitrephorones 1 and 2 protect from ferroptosis, reduce pro-inflammatory cytokine release, and suppress leukotriene biosynthesis.** (**A**) Metabolic activity of HepaRG hepatocytes (1×10^4^) incubated with vehicle (DMSO, 0.5%) or mitrephorones alone or in the presence of RSL3 (0.2 µM) for 48 h, measured by MTT assay. (**B**) PBMCs (1.4×10^6^) were pretreated with vehicle (DMSO, 0.1%) or mitrephorones for 30 min and then stimulated with LPS for 4 h (TNF-α, IL-8) or 18 h (IL-1β, IL-6, IL-10, MCP-1, IL-12 (p70), IL-23, IL-1ra). Cytokine and chemokine expression was determined by ELISA. (**C**) PBMCs (3.84×10^6^) were treated with vehicle (DMSO, 0.1%) or **2** for 30 min and stimulated with LPS for the indicated time points. NFKBIA phosphorylation and protein levels were determined by Western blot (representative of *n* = 3 independent experiments). Uncropped blots are shown in the raw image file. The vehicle controls are identical to Waltl et al. 83. (**D-F**) PBMCs (5×10^6^) were preincubated with vehicle (DMSO, 0.1%) or mitrephorones for 10 min and then stimulated with A23187 for another 10 min. (**D**, left) ALOX5 products. (**E**) Heatmap of mean percentage changes in oxylipin and fatty acid levels relative to vehicle. (**F**) PGE_2_ and PUFA levels. (**D**, **G**) M1-like or M2-like macrophages (1-2×10^6^) were treated with vehicle (DMSO, 0.1%) or mitrephorones for 15 min and stimulated with SACM (1%) for 180 min. (**D**, right) ALOX5 product formation in M1-like macrophages. (**G**) Levels of ALOX5 products, PTGS products, and 8,9-EET in M2-like macrophages. Mean (**B**, **E**) or mean ± SEM (**A**) and single data (**C**, **D**, **F**, **G**) from *n* = 3 (**A**, **C**), *n* = 3-4 (**B**, **D**, **E**, **F**) and *n* = 4 (**G**) independent experiments. *P* values given *vs.* vehicle control (**A-D**,** F**,** G**); ordinary one-way ANOVA of log data + Tukey's *post hoc* tests (**A**) and repeated measures one-way ANOVA of log data (**B**) or mixed-effects model (REML) ANOVA of log data (**B**,** D**,** F**,** G**) + Dunnett's *post hoc* tests. Two-tailed paired Student's *t* test of log data for pairwise comparisons as indicated by bars and *P* values in brackets (**C**, **D**). The vehicle controls shown in **B-G** are identical to those published previously 83. EPA, eicosapentaenoic acid; HDHA, hydroxydocosahexaenoic acid; HDPA, hydroxydocosapentaenoic acid; HEPE, hydroxyeicosapentaenoic acid; (di)HETE, (di)hydroxyeicosatetraenoic acid; TX, thromboxane.

Our search for hepatoprotective natural products combining anti-ferroptotic and anti-inflammatory properties led to the discovery of two closely related diterpenoids, **1** and **2** (**Figure 1**). Both compounds effectively suppressed ferroptosis induced by the GPX4 inhibitor RSL3 in human HepaRG hepatocytes (**Figure 2A**), used as a surrogate for normal hepatocytes 103-105.

As a substitute for hepatic immune cells, including Kupffer cells, we employed human primary PBMCs and monocyte-derived M1 and M2 macrophages, which, like Kupffer cells, release a variety of immunomodulatory mediators 106-109. Compounds **1** and **2** reduced the expression of pro-inflammatory and immunostimulatory cytokines and chemokines (IL-1β, IL-12 (p70) > IL-8, TNF-α), with IL-23 expression additionally affected by **1** in LPS-challenged PBMCs (**Figure 2B**, **Figure S1A**), seemingly independent of an interference with NF-κB signaling. Thus, compound **2** did not substantially affect NFKBIA phosphorylation or subsequent proteasomal degradation (**Figure 2C**). Acute cytotoxicity of the compounds was excluded under our experimental conditions (24 h). Neither compound reduced PBMC viability at 1 µM up to 48 h (**Figure S1B**). Mild cytotoxic effects of **2** (but not **1**) were observed at 10 µM starting at 48 h, measured as decreased metabolic activity, without reaching significance and without compromising membrane integrity (**Figure S1B**).

In addition, compounds **1** and **2** potently inhibited ALOX5 product formation in activated PBMCs and macrophages, while especially **2** moderately increased PGE_2_ and prostaglandin D_2_ (PGD_2_) levels in A23187-activated PBMCs (**Figure 2D-F**, **Figure S2A**,** B**, **Table S2**), but not, or only minimally, in activated macrophages (**Figure S2A**,** C**), possibly by redirecting the common substrate AA/20:4. PGE_2_ and PGD_2_ exhibit both pro- and anti-inflammatory activities 25, 33, 110-112. Levels of free PUFAs, including AA/20:4, were not substantially decreased, with one exception: compound **1** reduced PUFA release at higher concentrations (10 µM) in PBMCs (**Figure 2E**,** F**) and, to a lesser extent, in macrophages (**Figure S2A**,** D**), likely contributing to its greater efficacy compared to **2** in suppressing ALOX5 product formation in PBMCs. Due to variability between datasets, largely reflecting inter-individual differences among platelet donors, fold changes shown in the heatmap in **Figure 2E** do not necessarily indicate statistical significance. Heatmaps are therefore used throughout the manuscript solely for overview purposes, whereas conclusions are primarily based on bar or radar charts displaying statistical analyses (**Figure 2F**), with comprehensive datasets provided in the Supplementary Material (**Table S2**).

Conversely, compound **2** was more effective than **1** in suppressing ALOX5 product formation in M1 macrophages challenged with SACM, a physiological stimulus (**Figure 2D**). In activated M2 macrophages, **2** increased ALOX5 product formation by upregulating the production of 5*S*,6*R*-dihydroxyeicosatetraenoic acid (5*S*,6*R*-diHETE) (**Figure 2G**, **Figure S2A**, **Table S3**), a non-enzymatic hydrolysis product of LTA_4_ 93. In these cells, this increase correlated with reduced PTGS product formation, mainly prostaglandin F_2α_ (PGF_2α_) and thromboxane B_2_ (TXB_2_), and tendentially elevated cytochrome P450 monooxygenase-derived 8,9-epoxyeicosatrienoic acid (8,9-EET) levels (**Figure 2G**, **Figure S2A**), an anti-inflammatory mediator that limits inflammation and suppresses cell death 47.

Other lipid mediators were not or less affected (**Figure 2E**, **Figure S2A**), except for increased 12-lipoxygenase (ALOX12)/ALOX15 product formation, particularly in activated M1 macrophages treated with **1** (**Figure S2A**,** D**). These products serve as precursors of SPMs that were proposed to suppress leukocyte infiltration and activation, and promote phagocytosis, bacterial clearance, efferocytosis, and tissue repair 31, 34, 113-115. However, the increased ALOX12/15 product levels upon treatment with **1** did not translate into increased biosynthesis of SPMs or their isomers (SPM/iso), including protectins (PD/iso) or maresins (MaR/iso) (**Figure S2A**,** D**), whose formation is independent of ALOX5 30, 31, 116.

Collectively, compounds **1** and **2** protect liver cells from ferroptosis and limit the production of pro-inflammatory LTs, cytokines, and chemokines by activated innate immune cells, with effects on the lipid mediator profile strongly depending on the immune cell type and stimulus.

### Inhibition of membrane peroxidation through an unconventional mechanism

**Figure 3:**
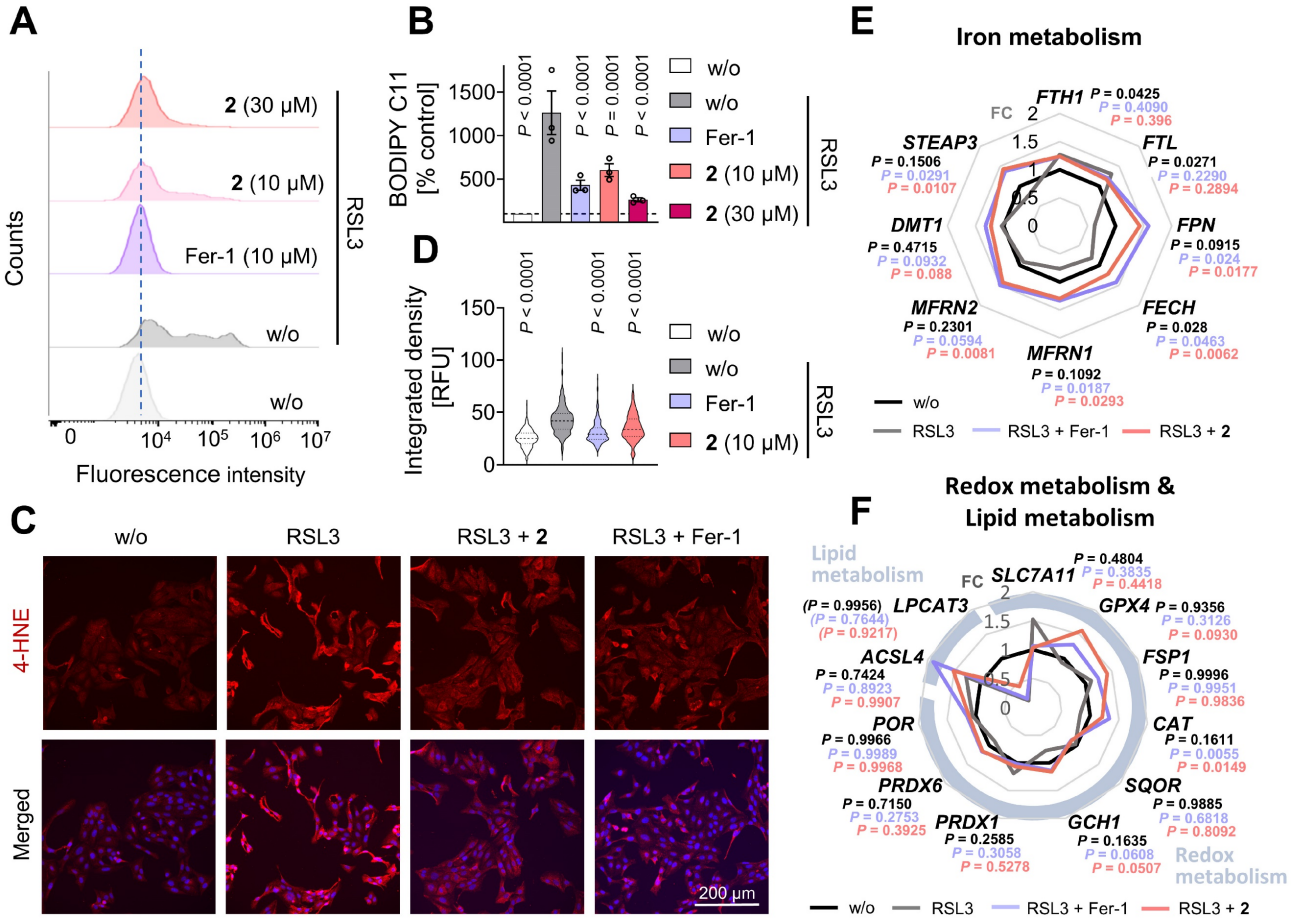
** Mitrephorone 2 suppresses lipid peroxidation and modulates ferroptosis-associated gene expression.** (**A**, **B**) Lipid peroxidation in HepaRG cells (2×10⁵ cells/well) was assessed using BODIPY 581/591 C11 staining. Cells were pretreated with vehicle (DMSO, 0.25%), mitrephorone **2**, or ferrostatin-1 (Fer-1, 10 µM) for 24 h, followed by exposure to vehicle (DMSO, 0.25%) or RSL3 (0.5 µM) for 2 h. Control experiments included cells treated with vehicle (DMSO, 0.5%) alone. (**A**) Representative histograms are shown. (**B**) Quantitative analysis of mean intensities of BODIPY-C11-stained cells. (**C**, **D**) HepaRG cells (2.5×10^5^ cells/well) were pre-treated (24 h) with vehicle (DMSO, 0.4%), mitrephorone **2** (10 µM), or Fer-1 (10 µM) and then challenged with RSL3 (0.5 µM, 2 h). (**C**) Immunofluorescence images (representative of 923-1152 single cells per group, *n* = 4) stained for 4-HNE and nuclei (DAPI). (**D**) Integrated density of the 4-HNE signal. (**E**, **F**) HepaRG cells (1×10⁶ cells/well) were treated for 48 h with vehicle (DMSO, 0.1 %), RSL3 (0.5 µM), or RSL3 (0.5 µM) in combination with mitrephorone **2** (10 µM) or ferrostatin-1 (Fer-1, 10 µM). Radar charts indicate the fold change (FC) of mRNA expression in cells treated with RSL3 alone or in combination with **2** or Fer-1, relative to vehicle-treated controls. Mean (**E**, **F**), mean and single data ± SEM (**B**), or mean, first and third quartiles, and data distribution (**D**) from *n* = 2 (**F**, *LPCAT3*), *n* = 3 (**B**, **E**, **F**, except *LPCAT3*), or *n* = 4 (**D**) independent experiments. *P* values given *vs.* RSL3-treated controls; repeated measures one-way ANOVA (**B**) of log data (**E**, **F,** except *LPCAT3*), mixed-effects model (REML) ANOVA of log data (**F**, *LPCAT3*) + Dunnett's *post hoc* tests, or ordinary one-way ANOVA (**D**) + Dunnett's *post hoc* tests.

The diverse pathways leading to ferroptosis generally converge on elevated labile ferrous iron pools, dysregulated glutathione metabolism, and/or impaired antioxidative defense and lipid quality control, ultimately causing uncontrolled oxidative membrane stress and phospholipid peroxidation in a metabolic state-dependent manner 66, 70. To confirm that **2** suppresses lipid peroxidation, HepaRG hepatocytes were challenged with the GPX4 inhibitor RSL3, stained with the lipid reactive oxygen species (ROS)-sensitive probe BODIPY-C11, and analyzed by flow cytometry (**Figure 3A**,** B**, **Figure S3A**). In a complementary approach, 4-hydroxynonenal (4-HNE), an oxidative lipid degradation product 65, was visualized by immunofluorescence microscopy (**Figure 3C-D**). As expected, treatment with **2** (10 µM) markedly reduced both membrane peroxidation readouts (**Figure 3A-D**). In addition, **2** attenuated the dysregulation of multiple ferroptosis markers, including *FPN*, *FECH*,* MFRN1*, *STEAP3*, *SLC7A11*, *CAT*, *GCH1*, *PRDX1*, *PRDX6*, and *LPCAT3*, at the mRNA level in RSL3-challenged hepatocytes (**Figure 3E**,** F**). Significant effects were observed for *FPN* and *CAT*, and the overall expression profile closely resembled that induced by the selective ferroptosis inhibitor ferrostatin-1. In contrast, the RSL3-induced upregulation of *FTH1* and *FTL* expression was not, or only marginally, affected, by either **2** or ferrostatin-1 (**Figure 3E**).

**Figure 4:**
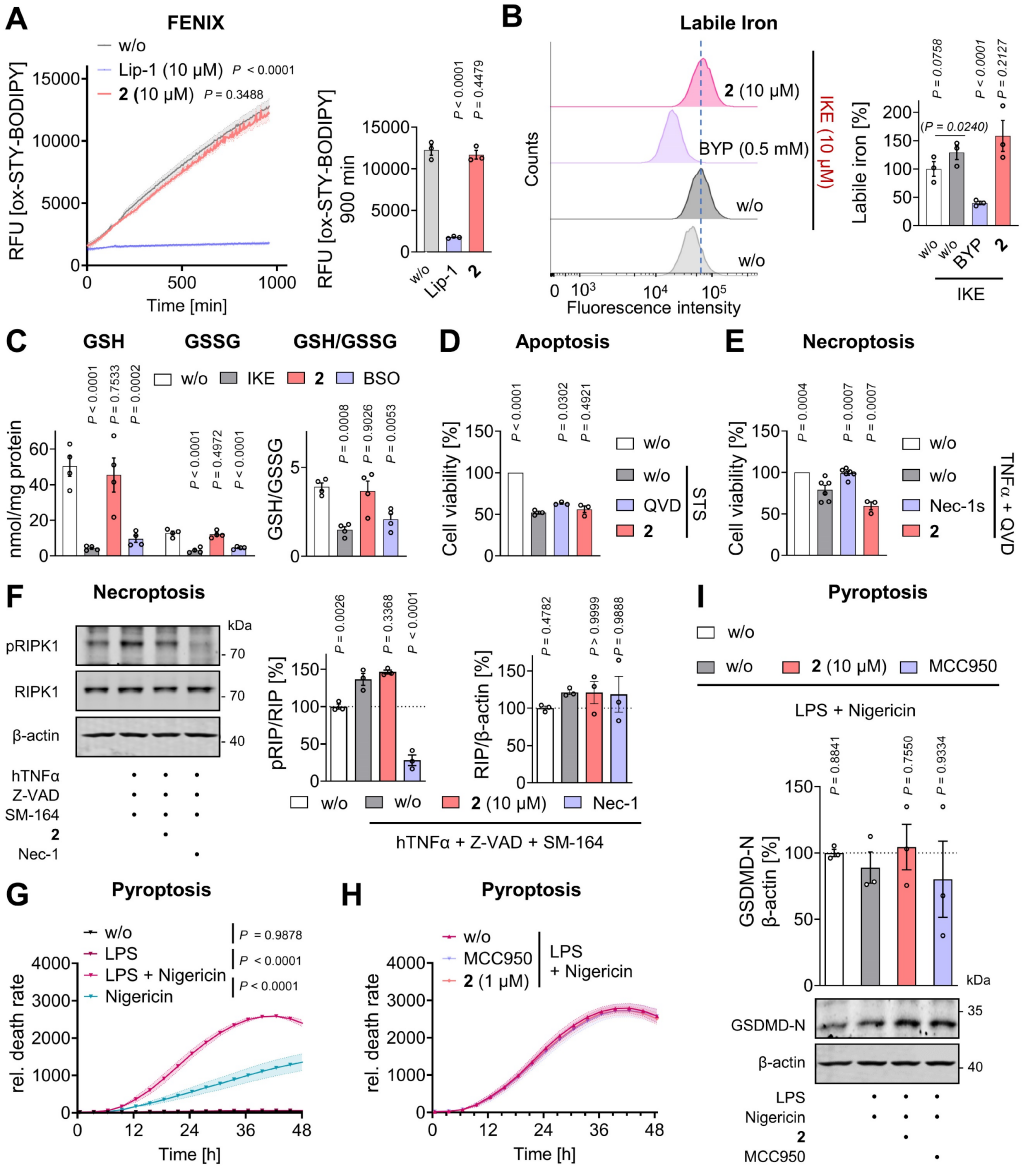
** Mitrephorone 2 inhibits ferroptosis through an unconventional mechanism.** (**A**) Time-course analysis of PC peroxidation in liposomes treated with mitrephorone **2** (10 µM) or liproxstatin-1 (Lip-1; 10 µM), measured using STY-BODIPY in the FENIX assay. Right panel: Bar graph showing the peroxidation levels at 900 min, based on the data presented in the left panel. (**B**) Labile iron levels in HepaRG cells (2.5×10^5^ cells/well) pretreated with vehicle (DMSO, 0.25%) or **2** (10 µM) for 24 h, followed by ferroptosis induction with vehicle (DMSO, 0.25%) or IKE (10 µM) for an additional 24 h, assessed using FerroOrange staining. 2,2′-Bipyridine (BYP, 0.5 mM) was included as a control. Representative histograms of *n* = 3 independent experiments are shown. Right panel: Quantification of mean fluorescence intensities derived from flow cytometry analysis of the FerroOrange-stained cells. (**C**) Cellular GSH and GSSG levels, as well as the GSH/GSSG ratio, were measured in HepaRG cells (3.6×10^5^ cells/well) treated with vehicle (DMSO, 0.1%), IKE (10 µM), BSO (20 µM), or **2** (10 µM) for 24 h. (**D**) Apoptosis was induced in HepaRG cells (1×10^4^ cells/well) using staurosporine (STS; 50 nM). Cells were co-treated with vehicle (DMSO, 0.5%) or **2** (1 µM), and metabolic activity was assessed after 48 h using the MTT assay. The pan-caspase inhibitor Q-VD-OPh (QVD, 50 µM) was used as control. (**E**) Necroptosis was induced by TNF-α (10 ng/mL) in the presence of QVD (50 µM). HepaRG cells (1×10^4^ cells/well) were co-treated with vehicle (DMSO, 0.5%) or **2** (1 µM), and metabolic activity was determined by MTT assay after 48 h. Necrostatin-1s (Nec-1s, 50 µM) was included as control. (**F**) RIPK1 protein expression and RIPK1 phosphorylation determined by Western blot (representative of *n* = 3 independent experiments). Uncropped blots are shown in the raw image file. Necrostatin-1 (Nec-1, 50 µM) was included as control. (**G, H**) Pyroptosis was induced by nigericin (10 µM) in LPS (10 ng/mL)-primed HepaRG cells (1×10^4^ cells/well). Cells were co-treated with vehicle (DMSO, 0.5%) or **2** (1 µM), and cell death was quantified by propidium iodide staining. The NLRP3 inhibitor MCC950 (1 µM) was used as a control. (**I**) Cleaved gasdermin levels determined by Western blot (representative of *n* = 3 independent experiments). Uncropped blots are shown in the raw image file. Mean ± SEM (**A**,** G**, **H**) or mean ± SEM and single data (**A**-**E**, **F**, **I**) from *n* = 3 (**A**,** B**, **D**, **E**: mitrephorone **2**-treatment, **F**, **I**), and *n* = 4 (**C**, **H**), *n* = 6 (**E**: except mitrephorone **2**-treatment, **G**) independent experiments. *P* values given *vs.* vehicle controls (**A**, **C**), *vs.* IKE-treated cells (**B**) or *vs.* respective cell death inducer (**D**-**I**); repeated measures two-way ANOVA + Dunnett's *post hoc* tests (**A,** left panel, **G**, **H**), repeated measures one-way ANOVA (**A**, right panel, **C**, **E, F**, **I**) of log data (**B, D**) + Dunnett's *post hoc* tests. Two-tailed paired Student's *t* test of log data for pairwise comparisons as indicated by bars and *P* values in brackets (**B**).

To obtain initial insights into the mechanism underlying the anti-ferroptotic activity of **2**, we first examined whether it directly interferes with membrane lipid peroxidation as lipophilic radical trap or modulates iron or glutathione metabolism. In contrast to the ferroptosis inhibitor liproxstatin-1, compound **2** did not inhibit phospholipid peroxidation by a lipophilic radical generator in artificial membranes (**Figure 4A**). It also did not affect intracellular labile iron levels (**Figure 4B**, **Figure S3B**), intracellular glutathione (GSH) levels, glutathione disulfide (GSSG) levels, or the GSH/GSSG ratio, indicative of the cellular redox state (**Figure 4C**). Interestingly, compound **2** modulated the expression of several key ferroptosis-associated factors (*MFRN2*, *DMT1, GPX4*, *FSP1*) in RSL3-challenged hepatocytes, even in cases where RSL3 alone had no marked effect (**Figure 3E**, **F**). In other instances, the expression of ferroptosis regulators (*FPN*, *FECH*, *MFRN1*, *STEAP3*, *CAT*, *PRDX6*) was counter-regulated by **2** beyond a simple restoration of homeostasis. However, as comparable expression patterns were also observed for the selective ferroptosis inhibitor ferrostatin-1 (**Figure 3E**, **F**), we consider these transcriptional changes to be secondary consequences of ferroptosis inhibition rather than indicative of a primary molecular target. Together, these findings suggest that compound **2** inhibits ferroptosis via a non-canonical mechanism that does not primarily involve radical scavenging, regulation of iron availability, alteration of glutathione metabolism, or specific transcriptional regulation of major ferroptosis-associated proteins.

### Preferential inhibition of ferroptosis over alternative cell death programs

Multiple cell death programs besides ferroptosis have been implicated in tissue degeneration during metabolic liver disease, including apoptosis, necroptosis, and pyroptosis 117. We therefore investigated whether **2** preferentially inhibits ferroptosis or also modulates other cell death pathways. Apoptosis was induced in HepaRG hepatocytes using the pan-kinase inhibitor staurosporine. Necroptosis was triggered by TNF-α in the presence of the pan-caspase inhibitor z-VAD-FMK. All treatments robustly reduced metabolic activity as assessed by MTT assay, and this effect was attenuated by the selective apoptosis inhibitor Q-VD-OPh or the necroptosis inhibitor necrostatin-1s (**Figure 4D**,** E**). Necroptosis induction was further confirmed by phosphorylation of RIPK1 (**Figure 4F**), a key component of the necrosome 117. In contrast to its potent anti-ferroptotic activity (**Figure 2A**, **Figure 3**), compound **2** had little effect on metabolic activity following apoptosis induction (**Figure 4D**) and even enhanced the loss of metabolic activity upon necroptosis induction (**Figure 4E**), without affecting RIPK1 phosphorylation (**Figure 4F**).

To assess whether **2** modulates pyroptosis, HepaRG cells were primed with LPS and exposed to the NLR Family Pyrin Domain Containing 3 (NLRP3) inflammasome activator nigericin, a commonly used approach to induce pyroptosis in immune and non-immune cells 118, and in a limited number of studies in hepatocytes 119. Loss of membrane integrity was monitored by propidium iodide staining and automated live-cell microscopy. Nigericin induced cell death in HepaRG cells, which was further enhanced by LPS priming, as expected (**Figure 4G**). However, the selective NLRP3 inhibitor MCC950 did not prevent cell death (**Figure 4H**), and enhanced cleavage of gasdermin D into pore-forming fragments by LPS/nigericin-treatment - an established hallmark of pyroptosis 117 - was not detected (**Figure 4I**). These findings indicate that HepaRG cells are susceptible to nigericin-induced cell death under LPS-primed conditions without activation of canonical pyroptotic signaling. Consistently, compound **2** did not affect nigericin-induced cell death (**Figure 4H**) or gasdermin D cleavage (**Figure 4I**), further supporting its preferential protection against ferroptosis among the cell death programs investigated.

### Mitrephorones antagonize 5-lipoxygenase-activating protein (ALOX5AP)

**Figure 5:**
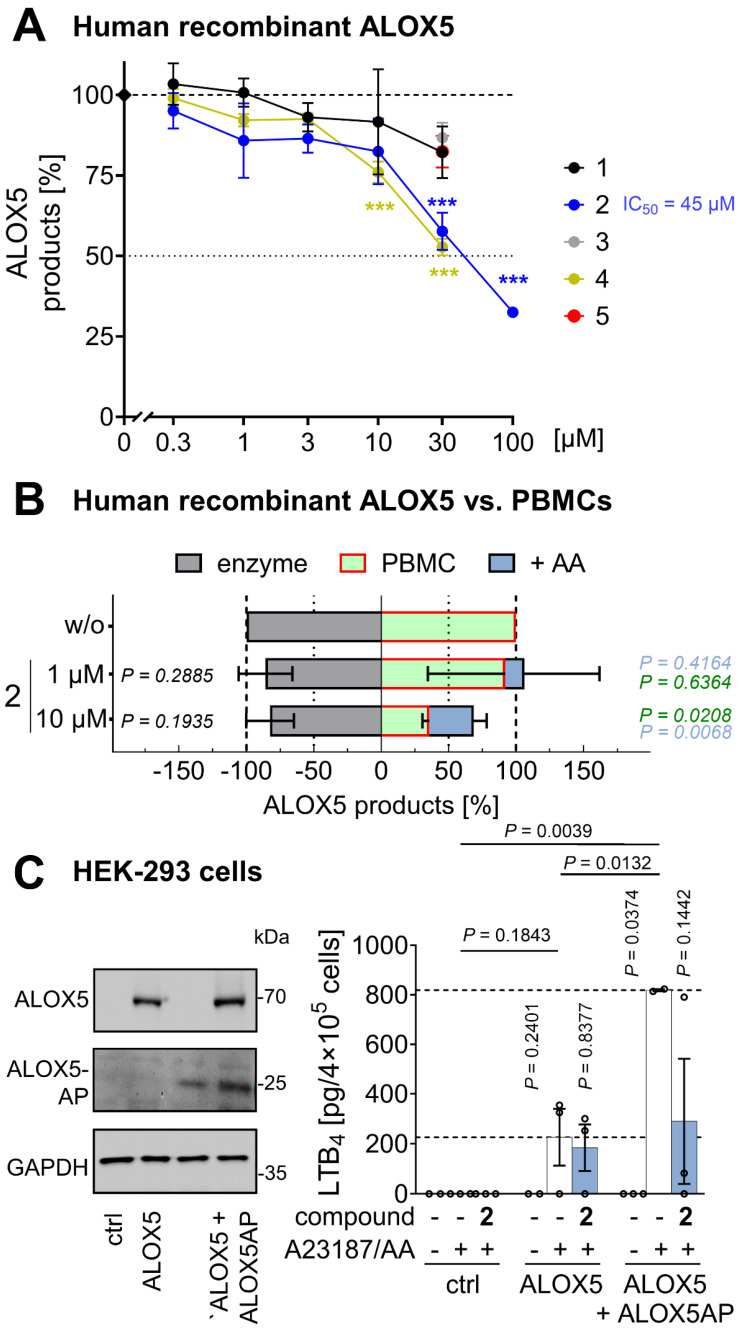
** Mitrephorone 2 antagonizes ALOX5AP.** (**A**, **B** left) Human recombinant ALOX5 was pretreated with vehicle (DMSO, 0.1%) or mitrephorones for 10 min, and enzymatic activity was determined. Data shown in panel **B** for the recombinant enzyme are identical to panel **A**. (**B**, right) PBMCs (5×10^6^) were preincubated with vehicle (DMSO, 0.1%) or **2** for 10 min and then treated with A23187 in the presence or absence of exogenous AA/20:4 to assess the effect of AA/20:4 on ALOX5 product formation. (**C**) HEK-293 cells were transfected with an empty control vector ('ctrl') or expression vectors for ALOX5 or ALOX5AP. Protein levels of ALOX5 and ALOX5AP were determined by Western blot (representative of *n* = 3 independent experiments); uncropped blots are shown in the raw image file. To assess the capacity for LTB_4_ formation, cells were preincubated with vehicle (DMSO, 0.1%) or **2** (10 µM) for 15 min, followed by treatment with A23187 and AA/20:4. Mean ± SEM (**A**, **B**) and single data (**C**) from *n* = 3 (**A**, **B** left panel), *n* = 2-3 (**C**), and *n* = 4 (**B** right panel) independent experiments. *P* values given *vs.* the respective vehicle control (**B**,** C**) or compound-treated PBMCs (**B** blue *P* values) or ***P < 0.001 *vs.* vehicle control (**A**); repeated measures one-way ANOVA of log data (**A 1**, **2** and** 4**) or mixed-effects model (REML) ANOVA (**C**) of log data (**B** gray and green) + Dunnett's *post hoc* tests. Two-tailed paired Student's *t* test of log data for pairwise comparisons (**A 3** and **5**;** B** blue *P* values) as indicated by bars (**C**). The vehicle controls shown in **A** and** B** are identical to those published previously 83.

ALOX5 product formation can be suppressed by limiting AA/20:4 supply, inhibiting ALOX5 or LTA_4_ hydrolase, antagonizing ALOX5AP, or interfering with ALOX5-activating pathways 31, 98. Because AA/20:4 levels were not reduced by **2** at concentrations that effectively decreased ALOX5 product formation (**Figure 2D**,** E**, **Figure S2A**,** D**), impaired AA/20:4 supply was excluded as a primary mechanism. Compounds **1** and **2** also did not significantly inhibit human recombinant ALOX5 in a cell-free assay (1 and 10 µM), ruling out direct enzyme inhibition as a major factor (**Figure 5A**).

Additionally, LTB_4_ and 20-OH-LTB_4_ were not preferentially suppressed relative to other ALOX5 products, excluding LTA_4_ hydrolase as a target (**Figure 2E**, **Figure S2A**). ALOX5AP mediates transfer of AA/20:4 from cytosolic phospholipase A_2α_ (PLA2G4A, cPLA_2α_) to ALOX5, but becomes unnecessary when AA/20:4 is supplied exogenously 32. To test for potential ALOX5AP antagonism, PBMCs were treated with **2**, and ALOX5 product formation was induced by Ca²⁺ influx and exogenous AA/20:4. As expected for a ALOX5AP antagonist, the ability of **2** to suppress ALOX5 products was strongly diminished in the presence of excess AA/20:4 (**Figure 5B**).

To confirm ALOX5AP as a target of **2**, we overexpressed ALOX5 alone or together with ALOX5AP in human embryonic kidney (HEK)-293 cells, which otherwise lack efficient ALOX5 product formation. Robust LTB_4_ formation upon treatment with A23187 and AA/20:4 was observed in ALOX5-expressing cells and was further enhanced by co-expression of ALOX5AP (**Figure 5C**). Compound **2** suppressed LTB_4_ formation in cells co-expressing ALOX5 and ALOX5AP, whereas its inhibitory effect was markedly reduced in cells expressing ALOX5 alone (**Figure 5C**), supporting ALOX5AP as the primary cellular target mediating ALOX5 inhibition by **2**.

### Structural requirements for anti-ferroptotic and anti-inflammatory activity

To define the structural features in compounds **1** and **2** responsible for ferroptosis protection, ALOX5AP antagonism, and repressed cytokine expression, we screened a small library of derivatives (**3**-**5**) (**Figure 1**) 82. These compounds lacked the 9-oxo group and featured additional modifications in rings B and C, including 4b,10a-dihydroxylation (**3**), 10a-hydroxylation plus Δ4b-desaturation (**4**), and more extensive structural changes (**5**).

All three derivatives retained moderate anti-ferroptotic activity, comparable to **1** but lower than **2** (**Figure 2A**), yet largely lost the ability to suppress ALOX5 product formation (**Figure 2D**, **Figure S2A**), suggesting that the 9-oxo group is critical for ALOX5AP antagonism. None of the derivatives exhibited acute cytotoxicity in PBMCs within 48 h (**Figure S1B**).

Cytokine expression revealed a more differentiated pattern: compound **3** resembled **2**, significantly reducing IL-1β and IL-23 levels, and showing trends toward decreased TNF-α, IL-8 and IL-12 levels (**Figure 2B**, **Figure S1A**). Compounds **4** and **5** were considerably less active and displayed more heterogeneous responses across datasets (**Figure 2B**, **Figure S1A**). Notably, the shared structural modification of **2** and **3** is the 10-hydroxy group, which replaces the 5-oxo group present in **1** and **4**.

Together, these findings indicate that mitrephorones are effective anti-ferroptotic agents, with the 9-oxo group required for ALOX5AP inhibition and the 10-hydroxy group likely contributing to pronounced suppression of pro-inflammatory cytokine and chemokine expression.

### Mitrephorones manipulate the capacity of PBMCs to produce lipid mediators

**Figure 6:**
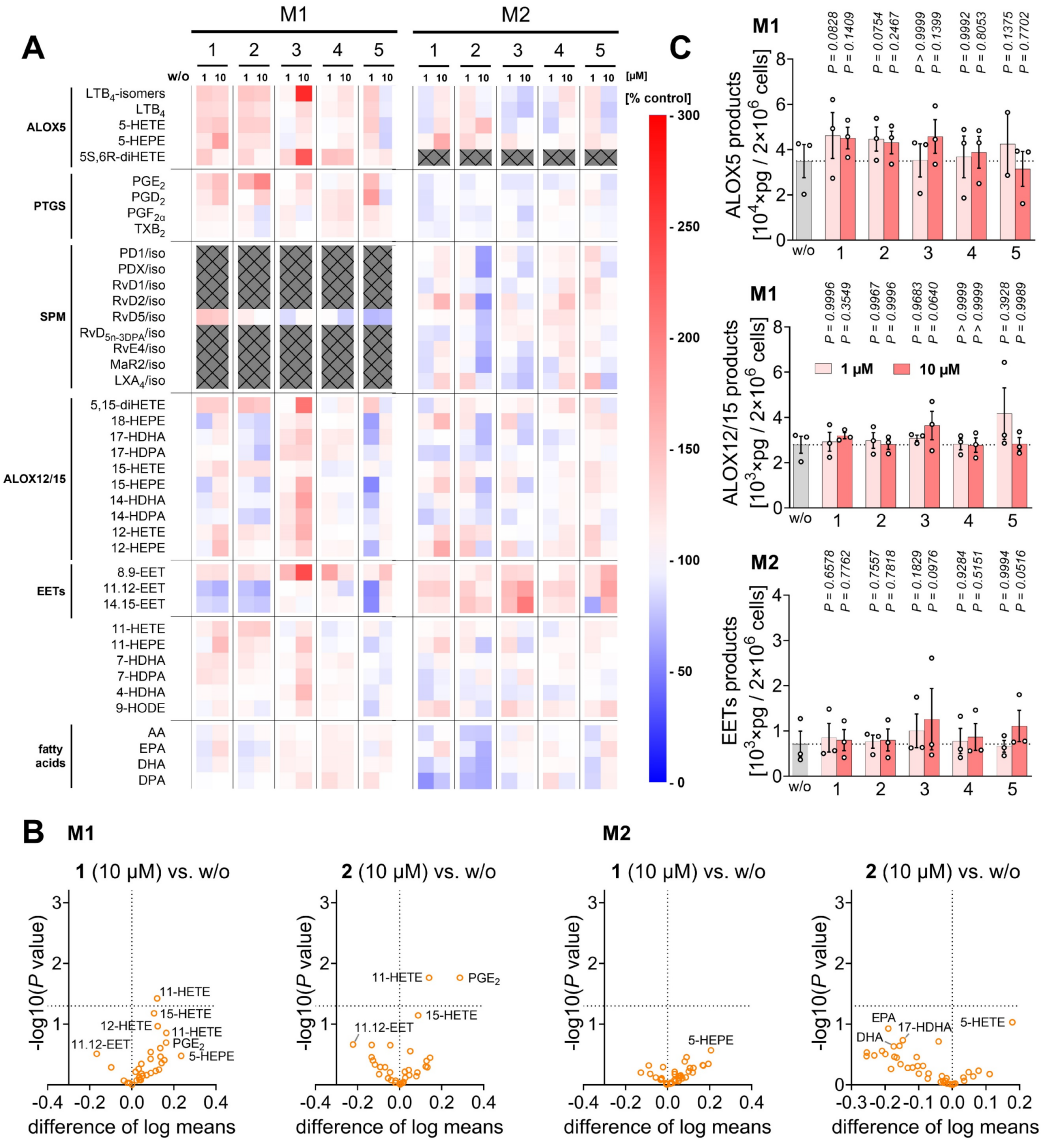
** Effect of mitrephorones on lipid mediator profiles during macrophage polarization.** Macrophages (2×10^6^) were treated with vehicle (DMSO, 0.1%) or mitrephorones during polarization to M1- or M2-like subtypes. Cells were stimulated with SACM (1%) for 180 min, and lipid mediator profiles were analyzed. (**A**) Heatmap of mean percentage changes in oxylipin and fatty acid levels relative to vehicle. Lipid mediators are grouped as ALOX5 products, PTGS products, SPM, monohydroxylated ALOX12/15 products, EETs, and free PUFAs. Crossed boxes indicate lipid mediators that were not detected. (**B**) Volcano plots depict differences in logarithmized means relative to vehicle control and -log10(*P* value) calculated *vs.* vehicle control; two-tailed multiple paired Student's *t* tests. (**C**) Formation of ALOX5 and ALOX12/15 products in M1-like macrophages and EETs in M2-like macrophages. Mean (**A**,** B**) or mean ± SEM and single data (**C**) from *n* = 2 (**5** 1 µM, M1) or *n* = 3 independent experiments. *P* values given *vs.* vehicle control (**C**); mixed-effects model (REML) ANOVA of log data or repeated measure one-way ANOVA of log data (**C**) + Dunnett's *post hoc* tests.

The studies on lipid mediator production described above were designed to capture short-term effects of mitrephorones, without considering changes arising from macrophage polarization or gene expression. To assess whether compounds **1**-**5** influence macrophage polarization and indirectly alter the lipid mediator profile, they were applied during the differentiation of monocyte-derived macrophages: toward the M1 phenotype by stimulation with LPS and interferon-γ, or toward the M2 phenotype by treatment with IL-4. Only a few significant changes were observed, primarily in the levels of PGE_2_ and monohydroxylated fatty acids, including 15-HETE, during polarization to M1-like macrophages (**Figure 6A**, **B**, **Table S3**). Modest trends were noted for increased ALOX5 products in M1-like macrophages (**1-3**), ALOX12/15 products in M1-like macrophages (**3**), and EETs in M2-like macrophages (**3**, **5**) (**Figure 6C**).

To assess whether compounds **1**-**5** alter the capacity of PBMCs to produce lipid mediators, cells were preincubated with the compounds for 48 h, then washed before eliciting lipid mediator production with the Ca²⁺ ionophore A23187. Because treatment with compound **2** (10 µM) slightly decreased PBMC metabolic activity (**Figure S1B**), lipid mediator concentrations were normalized to viable cell numbers to avoid overestimating inhibitory effects due to cytotoxicity.

**Figure 7:**
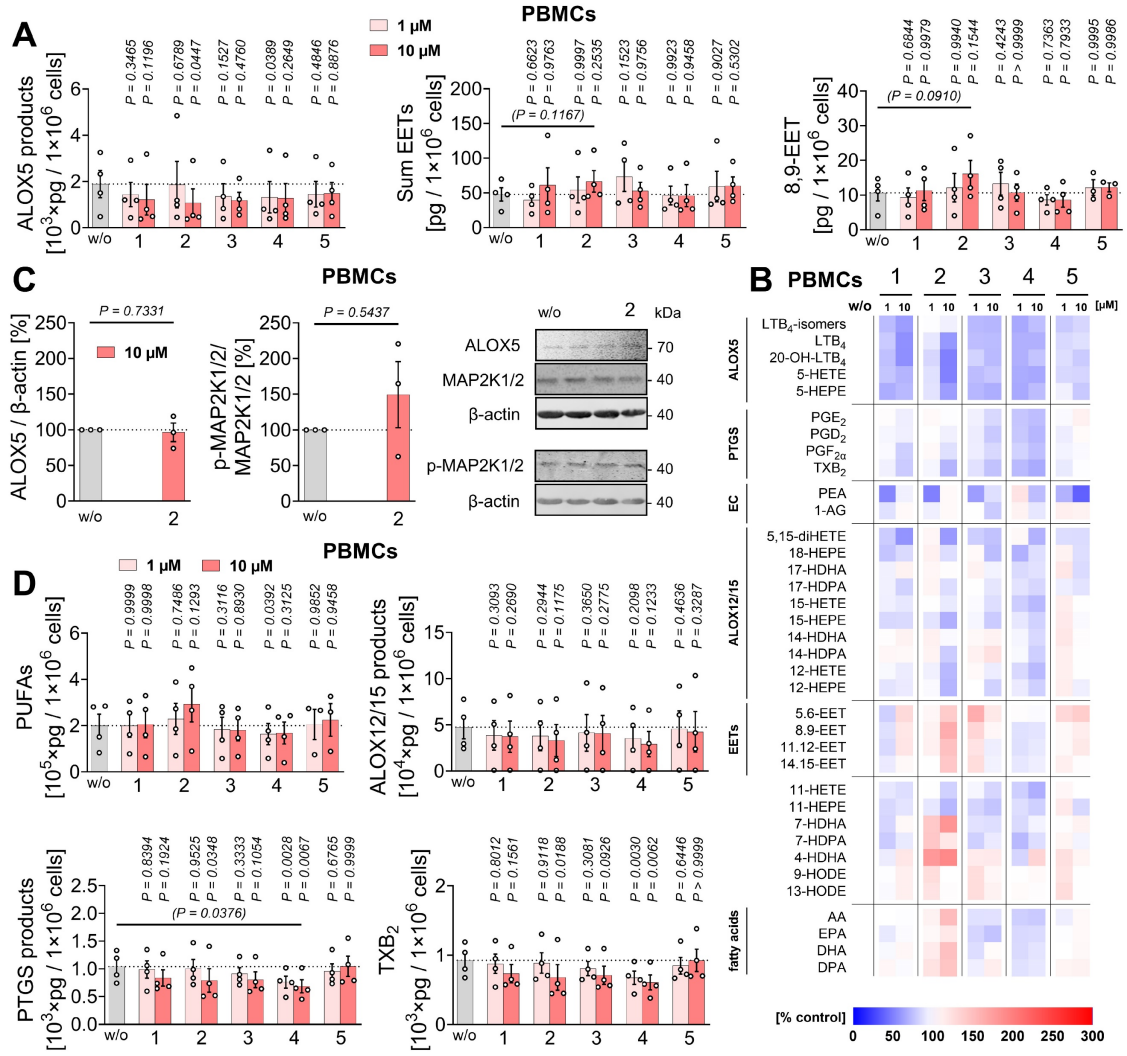
** Mitrephorone 2 reprograms PBMC lipid mediator production towards an anti-inflammatory profile.** (**A**-**D**) PBMCs (1×10^7^) were preincubated with vehicle (DMSO, 0.1%) or mitrephorones for 48 h, washed (**C**), and subsequently treated with A23187 to induce lipid mediator formation (**A**, **B**, **D**). (**A**) Levels of ALOX5 products, EETs, and 8,9-EET. (**B**) Heatmap of mean percentage changes in oxylipin and fatty acid levels relative to vehicle. Lipid mediators are grouped as ALOX5 products, PTGS products, endocannabinoids (EC), monohydroxylated ALOX12/15 products, EETs, and free PUFAs. (**C**) ALOX5 and MAP2K1/2 protein expression and MAP2K1/2 phosphorylation determined by Western blot (representative of *n* = 3 independent experiments). Uncropped blots are shown in the raw image file. The vehicle controls are identical to Waltl et al. 83. (**D**) Levels of PUFAs, ALOX12/15 products, PTGS products, and TXB_2_. Mean (**B**) or mean ± SEM and single data (**A**,** C**,** D**) from *n* = 3 (**C**) or *n* = 3-4 (**A**, **B**, **D**) independent experiments. *P* values given *vs.* vehicle control (**A**,** C**,** D**); repeated measures one-way ANOVA or mixed-effects model (REML) ANOVA of log data (**A**, **D**) + Dunnett's *post hoc* tests and two-tailed paired Student's *t* test of log data for pairwise comparisons as indicated by bars and *P* values (**C**) in brackets (**A**, **D**). The vehicle controls shown in** A-D** are identical to those published previously 83.

Compounds **1** and **2**, as well as **3** and **4**, which were less potent in short-term incubations, showed a similar profile in the long-term experiments, characterized by inhibition of ALOX5 product formation (significant for **2** and **4**) and tendentially increased EET levels (mainly 5,6-EET and 8,9-EET), except for **4** (**Figure 7A**,** B**, **Table S4**). Mechanistically, the reduced ALOX5 product formation was not due to decreased ALOX5 expression or phosphorylation by phospho-mitogen-activated protein kinase kinase 1/2 (p-MAP2K1/2, p-MEK1/2) (**Figure 7C**), which promotes ALOX5 nuclear translocation 120. The modest reduction of PUFA release, particularly AA/20:4, may contribute to the decreased ALOX5 and PTGS product formation observed with **4**, whereas **2** actually tended to enhance AA/20:4 release (**Figure 7B**,** D**).

In contrast to short-term incubation, compounds **1**-**4** reduced overall PTGS-derived prostanoid formation (significant for **2** and **4**), with **2** tending to increase PUFA release (**Figure 7B**,** D**), potentially facilitating EET production (**Figure 7A**, **B**). While **1** and **2** preferentially decreased TXB_2_ levels (mainly derived from platelet contamination via PTGS1 and TX synthase in PBMC preparations 121, 122), **3** and **4** caused a more balanced reduction of major PTGS products (**Figure 7B**).

Overall, long-term treatment with **1**-**4** decreased ALOX5 and PTGS product formation, tended to increase EET levels, and exerted divergent different effects on PUFA release, with compounds **2** and **4** being most effective.

### Compound 2 depletes cholesteryl esters (CEs) and promotes a peroxidation-protective lipid composition in PBMCs

The content and composition of phospholipids and neutral lipids are dysregulated in liver pathologies such as steatosis 5, 123-125, control cellular susceptibility to peroxidative damage and ferroptosis 57, 70, and determine the fatty acids available for the production of immunoregulatory lipid mediators 31, 116, 126. Consequently, changes in the cellular lipid profile can have profound effects on liver function and disease progression 3, 127-129, while shaping ferroptosis sensitivity 57, 70 and the capacity for lipid mediator biosynthesis over time 116, 126, 130, 131.

**Figure 8:**
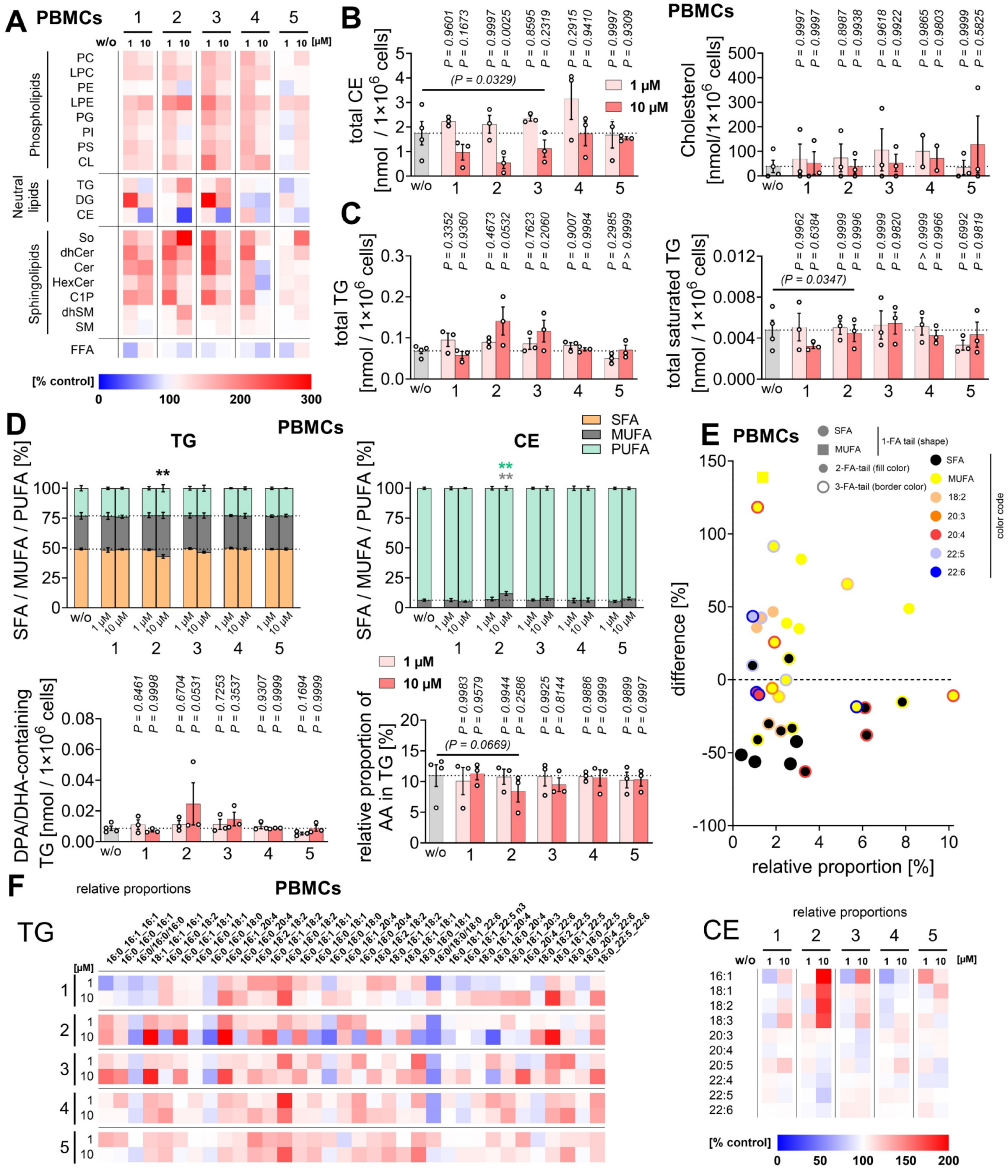
** Mitrephorone 2 reduces CE levels and reorganizes the fatty acid composition of neutral lipids in PBMCs.** (**A**-**F**) PBMCs (4×10⁶) were treated with vehicle (DMSO, 0.1%) or mitrephorones for 48 h, and cellular lipid composition was analyzed. (**A**) Heatmap of mean percentage changes in the amount of lipid subclasses relative to vehicle. (**B**) Total CE and cholesterol content. (**C**) Total TG and saturated TG levels. (**D**) Fatty acid composition of TG and CE, highlighting the fractions of SFAs, MUFAs, PUFAs, DPA/22:5 plus DHA/22:6, and AA/20:4. (**E**) Relative proportion of TG species (x-axis) plotted against their percentage differences following treatment with **2** (10 µM) compared to vehicle (difference of vehicle = 0%) (y-axis). (**F**) Heatmap of mean percentage changes in the relative proportions of TG and CE species relative to vehicle. Mean (**A**,** E**,** F**) or mean ± SEM (**D** top) and single data (**B**, **C**, **D** bottom) from *n* = 2 (**A** CL **3** - 1 µM, **B** cholesterol **4**), *n* = 4 (**A**-**F** w/o), or *n* = 3 (**A**-**F**) independent experiments. *P* values given *vs.* vehicle control (**B**-**D**) or **P < 0.01 *vs.* vehicle control (**D**); mixed-effects model (REML) ANOVA (**D** top) of log data (**B**,** C**, **D** bottom) + Dunnett's *post hoc* tests and two-tailed paired Student's *t* test of log data (**B**,** C**, **D** bottom) for pairwise comparisons as indicated by bars and *P* values in brackets. The vehicle controls shown are identical to those published previously 83. PC, phosphatidylcholine; LPC, lysophosphatidylcholine; PE, phosphatidylethanolamine; LPE, lysophosphatidylethanolamine; PG, phosphatidylglycerol; PI, phosphatidylinositol; PS, phosphatidylserine; CL, cardiolipin; TG, triglyceride; DG, diacylglycerol; CE, cholesteryl ester; So, sphingosine; (dh)Cer, (dihydro)ceramide; HexCer, hexosylceramide; C1P, ceramide-1-phosphate; (dh)SM, (dihydro)sphingomyelin; FFA, free fatty acid.

We investigated whether compounds **1**-**5** exploit these mechanisms by quantitatively analyzing 202 lipids from 18 subclasses of glycerophospholipids, sphingolipids, neutral lipids, and free fatty acids in PBMCs (**Figure 8A**, **Figure S4**, **Table S5**). Notably, compound **2** strongly reduced cellular levels of CEs (**Figure 8B**), a major component of lipid droplets alongside TGs 132, 133, which are positively associated with metabolic diseases such as MASLD and atherosclerosis 134-137. Free cholesterol levels did not decrease (**Figure 8B**), suggesting that the effect is not due to inhibition of *de novo* cholesterol biosynthesis, but rather relies on CE hydrolysis with subsequent cholesterol metabolism or excretion.

Intracellular TG levels, which like CEs are dysregulated in metabolic disease and can serve as an disease indicator 138-140, were increased (**Figure 8C**). This accumulation was mainly driven by monounsaturated fatty acid (MUFA)-containing species relative to SFA-containing ones, while the overall proportion of PUFAs remained constant despite AA/20:4 depletion (**Figure 8C-F**). Accordingly, compound **2** increased the proportion of MUFA-containing CEs at the expense of PUFA-containing species (**Figure 8D**,** F**).

These shifts in CE and TG fatty acid composition are consistent with a reduced susceptibility of lipid droplets to peroxidative damage 141, 142, given that lipid droplets have recently been identified as possible initiation sites for ferroptosis 143, 144. In addition, compound **2** tended to increase total TG levels (including PUFA-containing species at an unchanged ratio). This alteration, together with enhanced lipid droplet formation, has been linked to ferroptosis protection by buffering excess free PUFAs and thereby limiting their incorporation into membrane phospholipids, which would otherwise sensitize cells to ferroptosis 70, 145, 146.

**Figure 9:**
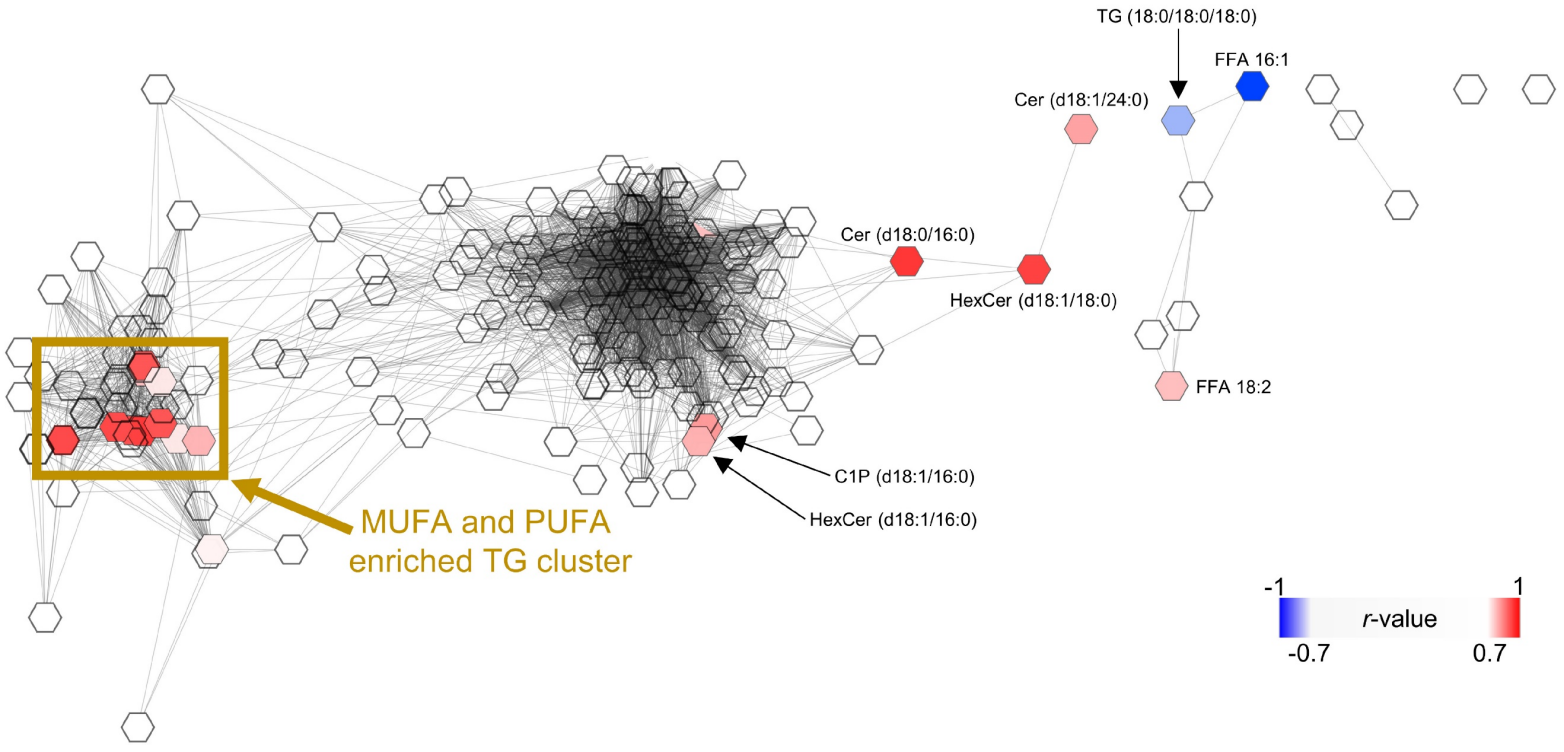
** Compound 1-5-induced changes in TG fatty acid composition of PBMCs correlate with ferroptosis protection in hepatocytes.** Correlation-based lipid network showing positive lipid-lipid correlations (*r* ≥ 0.7). Lipid species are represented as nodes (see **Figure S4** for detailed information). The network combines data from PBMCs treated with compounds **1**-**5** (1 and 10 µM each) for 48 h and was calculated from the mean percentage changes in lipid amounts relative to vehicle control from *n* = 2 (CL **3** - 1 µM), *n* = 4 (w/o), or *n* = 3 independent experiments. Node color indicates the strength and direction of Pearson correlations between mitrephorone **1**-**5** (1 µM)-induced changes in PBMC lipid species and the mean percentage changes in the metabolic activity of RSL3-treated HepaRG cells (**Figure 2A**) within 48 h relative to vehicle control: positive correlations (*r* ≥ 0.7) are shown in red, negative correlations (*r* ≤ -0.7) in blue; *n* = 2 (CL **3** - 1 µM), *n* = 4 (w/o), or *n* = 3 independent experiments.

To explore lipid alterations induced by mitrephorones that may affect ferroptosis sensitivity, we constructed a global lipid co-regulation network. Pearson correlations were calculated from percentage changes in 202 lipid species measured in PBMCs. Lipids with strong positive correlations (*r* ≥ 0.7) were positioned close to each other and connected by edges (**Figure 9**). Changes in individual lipid species induced by compounds **1**-**5** were then correlated with protection of human HepaRG hepatocytes from RSL3-induced ferroptosis, assessed by measuring metabolic activity via MTT assay. Positive and negative correlations are indicated in red and blue, respectively. The strongest positive correlations with ferroptosis protection were observed for TG and CE species, particularly within a cluster of MUFA- and PUFA-containing TGs. Similar results were obtained when the network was based on relative lipid proportions normalized to 100% of the respective lipid subclasses (**Figure S5**). These findings suggest that mitrephorone-induced changes in neutral lipids, abundant in lipid droplets, are associated with ferroptosis protection, although whether this relationship is causal remains unresolved.

**Figure 10:**
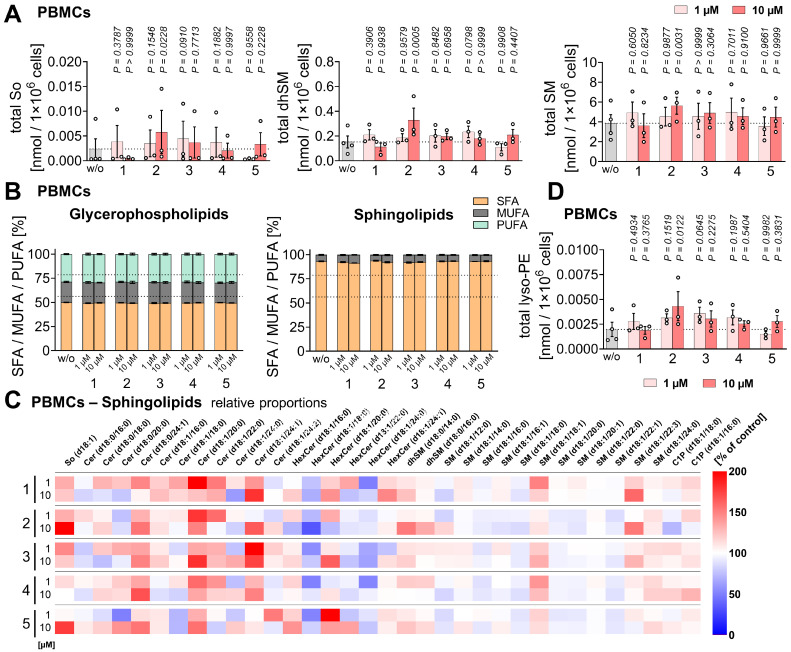
** Mitrephorone 2 leads to the accumulation of sphingolipids and a PE remodeling intermediate in PBMCs.** (**A**-**D**) PBMCs (4×10⁶) were preincubated with vehicle (DMSO, 0.1%) or mitrephorones for 48 h, and cellular lipid composition was analyzed. (**A**) Content of sphingosine (So) and (dihydro)sphingomyelin [(dh)SM]. (**B**) Fatty acid composition of glycerophospholipid and sphingolipid species, highlighting the fractions of SFAs, MUFAs, and PUFAs. (**C**) Heatmap of mean percentage changes in the relative proportions of sphingolipid species relative to vehicle. (**D**) Lyso-PE levels. Mean (**C**) or mean ± SEM (**B**) and single data (**A**,** D**) from *n* = 3 independent experiments. *P* values given *vs.* vehicle control; mixed-effects model (REML) ANOVA (**B**) of log data (**A**,** D**) + Dunnett's *post hoc* tests. The vehicle controls shown are identical to those published previously 83.

Membrane lipids are composed of glycerophospholipids and sphingolipids, the levels of which tended to increase upon treatment with compound **2** (**Figure 8A**), reaching significance for So, dhSM, and SM (**Figure 10A**, **Figure S6A**). The fatty acid composition, including PUFAs, was not markedly affected in either glycerophospholipids or sphingophospholipids (**Figure 10B**,** C**, **Figure S6B**), arguing against a redirection of AA/20:4 from TGs to phospholipids.

Interestingly, lysophosphatidylethanolamine (lyso-PE) levels were substantially increased in cells treated with **2** (**Figure 10D**, **Figure S4**), suggesting enhanced PE turnover by PLA_1_ or PLA_2_ isoenzymes 147. Since PE is a major phospholipid subclass peroxidized during ferroptosis 54-56, and specific PLA_2_s protect against ferroptosis by releasing oxidized fatty acids 148, 149, it is tempting to speculate that increased PE remodeling removes peroxidized or truncated fatty acids that might otherwise disrupt membrane architecture 57, 58.

Other mitrephorones (**1**, **3**, and **4**) showed similar but less pronounced effects on PBMC lipid composition, with some differences (**Figure 8A-D**,** E**, **Figure S4**, **Figure S6**). For example, compound **4** did not substantially decrease CE or increase TG levels (**Figure 8C**,** D**,** F**). Overall, our data indicate that compound **2** in particular induces changes in neutral lipid and phospholipid composition that may alleviate (cholesterol-driven) steatosis and other diseases while decreasing susceptibility to ferroptosis.

### Compound 2 reduces TG levels and mitigates loss of metabolic activity in an immunocompetent steatosis model *in vitro*

To better understand how the unique pharmacological profile of compound **2** might translate into clinical efficacy, we investigated its effects in four cell-based disease models at the intersection of (lipid) metabolic disease and inflammation. First, we assessed the uptake and accumulation of oxidized low-density lipoprotein (oxLDL) by human monocyte-derived macrophages, a critical step in atherosclerosis progression that generates foam cells and propagates arterial inflammation 137, 150. Second, we co-cultured HepaRG hepatocytes and PBMCs, the latter serving as a surrogate for hepatic Kupffer cells, and challenged this immunocompetent 2D hepatocyte model with three lipid-based stressors. i) A balanced mixture of PA/16:0 and OA/18:1 (1:2) to induce massive lipid overload, mimicking steatosis 138, 151; ii) excess saturated PA/16:0, causing severe lipotoxic stress 152, 153; or iii) polyunsaturated AA/20:4, which promotes peroxidative stress and sensitizes cells to ferroptosis when incorporated into membranes 56, 70, while simultaneously boosting the production of AA/20:4-derived pro-inflammatory lipid mediators 28.

**Figure 11:**
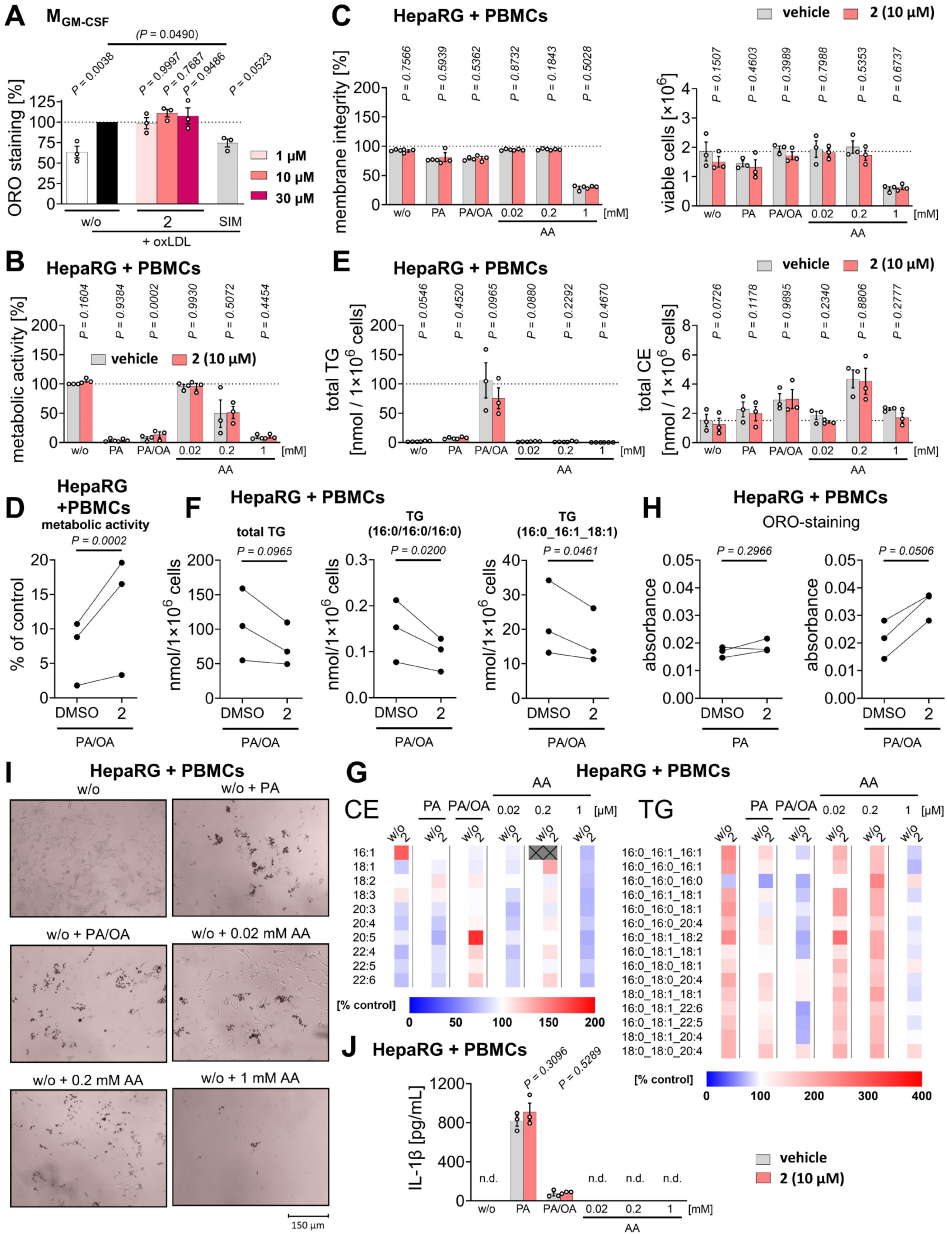
** Mitrephorone 2 alleviates lipid overload in cocultures of hepatocytes and PBMCs.** (**A**) Serum-starved M_GM-CSF_ (2.5×10⁵) were preincubated with vehicle (DMSO, 0.1%), **2**, or simvastatin ('SIM'; 100 µM) for 1 h and subsequently exposed to oxLDL for 48 h before Oil-Red-O (ORO) staining. (**B**-**J**) HepaRG cells and PBMCs were co-cultured (1:1) and preincubated with fatty acids (PA/16:0: 0.2 mM; PA/16:0 and OA/18:1: 1 mM, 1:2; AA/20:4 as indicated) for 24 h. Cells were then treated with vehicle (DMSO, 0.1%) or **2** (10 µM) and incubated for another 24 h. (**B**) Metabolic activity (MTT assay). (**C**) Membrane integrity and viable cell numbers. (**D**) Metabolic activity of PA/OA-treated cells (MTT assay). (**E**) Total TG and CE content. (**F**) Content of TG and selected TG species in PA/OA-treated cells. (**G**) Heatmap of mean percentage changes in the amount of TG and CE species relative to vehicle. Crossed boxes indicate CEs that were not detected. (**H**, **I**) ORO staining: absorbance of organic extracts (**H**) and microscopic images (**I**). (**J**) IL-1β levels; n.d., not detected. Mean (**G**) or mean ± SEM and single data (**A**-**C**, **E**, **J**) or interconnected single data (**D**,** F**,** H**) from *n* = 3 independent experiments. *P* values given *vs.* vehicle control (**A**-**F**, **H**, **J**); repeated measures one-way ANOVA of log data (**A**) + Dunnett's *post hoc* tests or two-tailed paired Student's *t* tests (**C** left) of log data (**B**, **C** right,** D**-**F**, **H**, **J**) for pairwise comparisons.

Compound **2** did not attenuate oxLDL-induced intracellular lipid accumulation in macrophages, as measured by Oil-Red-O staining (**Figure 11A**), nor did it reduce saturated or PUFA-induced stress in hepatocyte-PBMC co-cultures, as assessed by measuring metabolic activity (**Figure 11B**), viable cell number, and membrane integrity (**Figure 11C**). However, during lipid overload with PA/OA, **2** significantly increased cellular metabolic activity (**Figure 11B**,** D**) without affecting viable cell number or membrane integrity (**Figure 11C**). This increase in metabolic activity was accompanied by reduced levels of TGs, but not CE, reaching significance for individual species such as TG(16:0/16:0/16:0) (**Figure 11E**-**G**, **Table S6**), while Oil-Red-O staining of lipid droplets was not reduced and even tended to increase (**Figure 11H**,** I**, **Figure S7A**). In contrast, the peroxisome proliferator-activated receptor γ (PPARγ) agonist pioglitazone and, by trend, metformin and simvastatin, clinically used drugs that directly or indirectly target lipid metabolism in patients with metabolic syndrome 7-12, increased cellular TG levels (**Figure S8A**) and, in the case of simvastatin, reduced lipid droplet accumulation (**Figure S8B**,** C**) together with a trend toward lower CE levels (**Figure S8A**). However, none of these treatments increased metabolic activity (**Figure S8D**).

Interestingly, in unchallenged hepatocyte-PBMC co-cultures (**Figure 11E**, **Figure S7B**,** C**), compound **2** affected lipid metabolism similarly to PBMCs alone (**Figure 8**), depleting CEs (significant for AA/20:4-containing CEs) and tendentially enriching TGs, in contrast to its effect under PA/OA-induced lipid overload (**Figure 11E-G**). Compound **2** did not suppress the PA- or PA/OA-induced increase in cytokine expression, as exemplarily shown for IL-1β (**Figure 11J**).

In conclusion, in non-stressed hepatocyte-immune cell cultures reflecting pre-disease conditions, compound **2** adjusts neutral lipid metabolism, resulting in decreased CE levels that may contribute to prevention of metabolic disease. During acute lipid overload, **2** exhibits a distinct pharmacological profile, moderately buffering TG accumulation while enhancing cellular metabolic capacity under stress.

## Discussion

Chronic liver diseases are driven by a variety of endogenous and exogenous factors and are influenced by diet, lifestyle, and genetic predisposition 6, 49, 50, 80, 138, 154. Despite their mechanistic and phenotypic diversity, they share key processes that drive initiation and progression: metabolic dysregulation 155-158, low-grade persistent inflammation 159-161, and tissue degeneration with loss of function 51, 162. Our search for small molecules that simultaneously target these three pillars led to the identification of ent-trachylobane diterpenoids that (i) inhibit GPX4-dependent ferroptosis in hepatocytes, (ii) suppress the production of pro-inflammatory lipid mediators (i.e., LTs) and cytokines (e.g., IL-1β, IL-12), (iii) modestly enhance the levels of anti-inflammatory mediators (i.e., EETs), and (iv) redirect neutral lipid composition in immune cells and hepatocyte-immune cell co-cultures.

Mechanistically, mitrephorones exert anti-ferroptotic activity in hepatocytes by protecting membranes from phospholipid peroxidation under radical stress, as demonstrated for the most active compound **2**. Consistent with the absence of structural features characteristic of efficient radical scavengers, compound **2** requires a cellular context to confer protection against membrane peroxidation. Although the expression of several key factors involved in redox homeostasis, membrane quality control, iron metabolism, and lipid metabolism is modulated, these effects are largely comparable to those observed for the selective ferroptosis inhibitor ferrostatin-1. Moreover, central hallmarks of ferroptosis, including the labile iron pool and glutathione metabolism, remain unaffected, pointing toward an unconventional anti-ferroptotic mechanism. While the precise mechanisms remain incompletely defined, our data suggest that mitrephorone-mediated ferroptosis protection may involve adaptations in phospholipid and neutral lipid remodeling. Specifically, mitrephorones increase PE turnover, possibly facilitating the removal of membrane peroxides and truncated fatty acids 149, 163, 164. In parallel, they deplete PUFAs from CEs while increasing the MUFA ratio of both CEs and TGs, the principal constituents of the lipid droplet core 132, 133, and expand total TG levels, potentially buffering excess PUFAs, at least under non-disease conditions.

How the PUFA ratio impacts ferroptosis sensitivity is not fully understood and seems to be context-dependent. Similar to SFAs, whose incorporation into lipid droplets protects cells from lipotoxic stress 153, sequestration of PUFAs in lipid droplets is widely thought to reduce ferroptosis susceptibility by limiting their incorporation into membrane phospholipids 58, 165. Interestingly, lipid droplet biogenesis from exogenous fatty acids via ACSL3, an enzyme with substrate preference for palmitate 166, 167, has likewise been associated with increased ferroptosis resistance 165, 168-171. By contrast, lipid droplet peroxidation can induce ferroptosis in certain contexts 172, and selective autophagic degradation of lipid droplets has also been shown to promote ferroptosis 173, 174. How changes in lipid droplet fatty acid composition influence ferroptosis sensitivity remains less well understood, particularly under conditions where neither lipid droplet volume nor phospholipid composition are affected, and where it is unclear whether lipid droplet peroxidation alone can propagate oxidative damage to the plasma membrane. Mitrephorone **2** meets these criteria and thus enables us to address this question. It shifts TG fatty acid composition from SFAs toward MUFAs in PBMCs (used here as a surrogate for hepatic Kupffer cells), while conferring ferroptosis protection in hepatocytes. The total PUFA content of TG increased in parallel with total TG levels, leaving the PUFA proportion within TG unchanged. Although we cannot exclude that these effects arise from independent and possibly compensatory mechanisms, several observations argue for a causal link. First, ferroptosis can be initiated in different compartments and spread from the ER to the plasma membrane 143, 175. Second, while lipid droplet peroxidation has not been systematically studied, lipid droplets are highly dynamic and establish contact sites with the ER, mitochondria, and lysosomes 133, all of which can initiate ferroptotic lipid peroxidation 141-143, 172, 176.

Mitrephorone **2** shows a preference for suppressing ferroptosis in hepatocytes. In contrast, it did not prevent apoptosis or necroptosis, nor did it attenuate an undefined type of cell death triggered by pyroptosis inducers. Under necroptotic conditions, **2** even reduced cellular metabolic activity, indicating a potential context-dependent liability that warrants further investigation, particularly with respect to necroptosis-driven degenerative diseases.

Mitrephorones limit inflammation by antagonizing ALOX5AP. In addition, they modulate PUFA release and prostanoid formation, and interfere with the long-term capacity of innate immune cells, including M1 and M2 macrophages, to produce lipid mediators. Notably, M2 macrophages can arise from M1-like Kupffer cells during high-fat feeding 177, possess anti-inflammatory and tissue-regenerative properties 22, 31, 178, and are critically involved in the pathogenesis of liver disease 22, 178. The efficacy of mitrephorones, and in some cases even the direction of cytokine and lipid mediator regulation, varies considerably between experimental systems and stimuli. For instance, compounds **1** and **2** efficiently suppressed the expression of pro-inflammatory cytokines such as IL-1β in LPS-stimulated PBMCs, whereas **2** failed to reduce IL-1β levels in PBMC/hepatocyte co-cultures challenged with PA/16:0. These differences likely reflect distinct signaling mechanisms. LPS activates Toll-like receptor 4 (TLR4) signaling to induce cytokine expression 179, whereas PA/16:0 employs additional and less well-defined pathways. Reported mechanisms include immune cell activation via TLR4, voltage-gated potassium channels, lectin-type oxidized LDL receptor-1, and ROS generation 180-185. Differences in experimental design may also contribute. In PBMC cultures, compound **2** was applied before LPS stimulation, thereby interfering with the initiation of cytokine expression. In contrast, in PA/16:0-challenged co-cultures, **2** was added after the challenge, when the prerequisites for efficient cytokine expression were already established, potentially involving altered lipid mediator profiles 34, 186, 187.

The effects of compound **2** on neutral lipid metabolism are mixed and difficult to interpret in the context of clinical efficacy. While CE levels are favorably reduced in hepatocyte-immune cell co-cultures and in immune cells, TG levels tend to increase, with this pattern being reversed under metabolic stress caused by lipid overload. How such a lipid profile translates into pre-clinical settings remains to be determined and likely depends on the precise metabolic disease state. On the one hand, **2** seems to protect healthy or pre-disease cells from metabolic dysregulation, particularly when driven by cholesterol accumulation.

On the other hand, **2** appears to preferentially rebalance TG-driven lipid alterations, which may have adverse consequences in hypercholesterolemia. Importantly, the TG-lowering effect coincides with enhanced metabolic activity of hepatocyte-immune cell co-cultures in a simplistic *in vitro* MASLD model, suggesting protection against cytotoxic stress induced by lipid overload. However, the systemic consequences remain difficult to predict, as plasma TG and cholesterol clearance rely on hepatic lipid uptake, storage, and metabolism 3, 188, 189. Given these unique activities of **2** in rearranging lipid metabolism, the mitrephorone toolbox presented here provides a valuable resource to dissect these interrelationships and guide drug development. Small structural modifications enable, to some extent, the fine-tuning of cellular lipid profiles as well as lipid mediator and cytokine production, according to the requirements of specific disease contexts.

Despite the comprehensive mechanistic insights provided here into the action of **2** in primary cells, cell lines, and co-culture models, important questions regarding its precise molecular mechanism remain. We show that **2** inhibits ferroptosis by suppressing phospholipid peroxidation in a cellular context, without affecting other hallmark pathways of ferroptosis, including the glutathione-GPX4 axis, (phospho)lipid metabolism, or iron mobilization. With respect to lipid mediator modulation in innate immune cells, we attribute the most robust effect - the suppression of ALOX5 product formation - to antagonism of ALOX5AP. In contrast, the mechanistic basis for the inhibition of lipid peroxidation and the context-dependent modulation of other lipid mediator classes, as well as the direct molecular targets responsible for reduced TG levels and protection against lipid overload stress, remains unclear. Elucidation of these mechanisms will benefit from multi-omics approaches, including interactomics, ideally applied in a cell-type-specific manner.

Caution is also warranted when interpreting mitrephorone **2**'s potential hepatoprotective activity *in vivo* in metabolic liver disease. *In vivo* animal data are currently lacking, largely due to the limited availability of sufficient amounts of **2**, which requires a 10-step stereoselective synthesis 82. This limitation may be overcome by systematic optimization of the core structure of **2** to generate synthetically more accessible derivatives that retain the activity of the parent compound. Given the drug-like properties of compound **2** according to Lipinski's rule of five (M_r_ = 344, log*P* = 2.6, 1 hydrogen bond donor, 4 hydrogen bond acceptors) 190, the lipid-regulatory, anti-inflammatory, and anti-ferroptotic activities observed here at 1-10 µM fall within a pharmacologically relevant range, which now requires further delineation by pharmacodynamic and kinetic studies *in vivo*.

Few studies have previously addressed the biological activities of compounds **1** and **2**. Reported effects include disruption of membrane integrity in human cancer cell lines (KB > MCF-7 > H460 > Sf-268; EC_50_ = 20-90 µM) as well as antibacterial (*Micrococcus luteus*, *Mycobacterium smegmatis*; ≥ 63 µM) and antifungal activities (*Saccharomyces cerevisiae*, *Aspergillus niger; ≥ 63 µM*), with **1** being more potent than **2** 81. While the concentrations required for antitumor activity appear high, they might still be achievable *in vivo* after oral or intravenous administration, whereas antimicrobial effects are likely confined to cutaneous or gastrointestinal applications.

## Conclusion

We have identified mitrephorones as drug-like small molecules occupying a largely unexplored chemical space that qualify for further pre-clinical evaluation as hepatoprotective agents. They act on the three central pillars of degenerative liver diseases in hepatocytes, immune cells, and co-cultures - aberrant neutral lipid storage and metabolism, persistent inflammation, and cell death under metabolic stress, particularly ferroptosis. Their molecular mechanisms include i) effective inhibition of membrane peroxidation, ii) ALOX5AP antagonism, iii) NF-κB-independent suppression of pro-inflammatory cytokine release, iv) reduced inducible capacity of immune cells to produce lipid mediators, v) redirection of neutral lipids to lower intracellular CE or TG levels, vi) adaptations in phospholipid remodeling and neutral lipid fatty acid composition that counteract (per)oxidative membrane stress, and vii) potentially an upregulation of anti-inflammatory EETs, alongside broader changes in global lipid composition. Importantly, these activities are tunable by small structural modifications, providing access to a new spectrum of derivatives with optimized activity profiles tailored to specific disease contexts. Small molecules with such multi-targeted activity may overcome the limitations of single-pathway interventions in the treatment or prevention of necroinflammatory liver diseases, which otherwise progress toward cirrhosis, fibrosis, and hepatocellular carcinoma - an aspect that needs to be addressed in future preclinical studies.

## Supplementary Material

Supplementary Figures S1-S8 and Table S1.

Tables S2-S6 as well as raw images.

## Figures and Tables

**Table 1:** Quantitative MRM transitions of lipid mediators

Q1 [m/z]	Q3 [m/z]	rt [min]	ID	MRM window [s]	DP [V]	EP [V]	CE [eV]	CXP [V]	Standards
**351.3**	195.1	5.2	20-OH-LTB_4_		-80.0	-10.0	-24.0	-15.0	EX
**369.3**	169.1	6.3	TXB_2_	130	-80.0	-10.0	-22.0	-15.0	EX
**351.2**	271.0	6.7	PGE_2_		-120.0	-10.0	-20.0	-13.0	EX
**351.3**	189.1	6.8	PGD_2_		-120.0	-10.0	-20.0	-13.0	EX
**380.3**	141.2	6.9	d5-RvD_2_	120	-80.0	-10.0	-23.0	-14.0	IS
**353.3**	193.1	7.0	PGF_2α_	60	-80.0	-10.0	-34.0	-11.0	EX
**375.2**	175.1	7.0	RvD2		-80.0	-10.0	-30.0	-13.0	EX
**351.2**	235.1	7.3	LXA4		-80.0	-10.0	-20.0	-13.0	EX
**375.2**	215.1	7.4	RvD1		-80.0	-10.0	-26.0	-13.0	EX
**333.3**	115.1	8.4	RvE4		-80.0	-10.0	-22.0	-13.0	EX
**335.2**	195.1	9.2	LTB_4_-isomers	120	-80.0	-10.0	-22.0	-13.0	
**335.2**	201.0	9.3	5,15-diHETE	60	-50.0	-10.0	-30.0	-13.0	EX
**359.2**	153.1	9.3	PDX	120	-80.0	-10.0	-21.0	-9.0	EX
**359.2**	199.1	9.4	RvD5	60	-80.0	-10.0	-21.0	-13.0	EX
**359.2**	153.1	9.5	PD1	120	-80.0	-10.0	-21.0	-9.0	EX
**335.2**	195.1	9.7	LTB_4_	120	-80.0	-10.0	-22.0	-13.0	EX
**361.5**	143.1	9.7	RvD5_n-3DPA_		-60.0	-10.0	-20.0	-10.0	EX
**359.2**	221.0	10.2	MaR2		-80.0	-10.0	-20.0	-12.0	EX
**337.2**	207.1	10.5	14,15-DiHET	60	-60.0	-5.0	-20.0	-10.0	EX
**337.2**	167.1	10.7	11,12-DiHET	60	-30.0	-5.0	-30.0	-15.0	EX
**317.2**	259.1	10.9	18-HEPE		-80.0	-10.0	-16.0	-23.0	EX
**337.2**	127.1	11.0	8,9-DiHET	60	-60.0	-5.0	-30.0	-15.0	EX
**317.2**	219.1	11.1	15-HEPE	60	-80.0	-10.0	-18.0	-12.0	EX
**335.2**	115.1	11.1	5*S*,6*R*-diHETE	80	-80.0	-10.0	-20.0	-13.0	EX
**317.2**	167.1	11.2	11-HEPE	60	-80.0	-10.0	-19.0	-12.0	EX
**317.2**	179.1	11.3	12-HEPE	60	-80.0	-10.0	-19.0	-12.0	EX
**337.2**	145.0	11.5	5,6-DiHET	60	-70.0	-5.0	-20.0	-10.0	EX
**295.2**	171.0	11.6	9-HODE		-60.0	-10.0	-19.0	-13.0	EX
**295.2**	195.0	11.6	13-HODE		-60.0	-10.0	-25.0	-13.0	EX
**317.2**	115.1	11.6	5-HEPE	60	-80.0	-10.0	-18.0	-12.0	EX
**319.2**	219.1	11.8	15-HETE	60	-80.0	-10.0	-19.0	-12.0	EX
**343.2**	245.1	11.8	17-HDHA	60	-80.0	-10.0	-17.0	-14.0	EX
**319.2**	167.1	12.0	11-HETE	60	-80.0	-10.0	-21.0	-12.0	EX
**319.2**	179.1	12.1	12-HETE	60	-80.0	-10.0	-21.0	-12.0	EX
**343.2**	205.1	12.1	14-HDHA	60	-80.0	-10.0	-17.0	-14.0	EX
**343.2**	141.1	12.3	7-HDHA	60	-80.0	-10.0	-18.0	-15.0	EX
**319.2**	219.2	12.4	14.15-EET	60	-60.0	-10.0	-20.0	-10.0	EX
**345.2**	247.1	12.4	17-HDPA		-80.0	-10.0	-17.0	-14.0	
**345.2**	207.1	12.4	14-HDPA		-80.0	-10.0	-17.0	-14.0	
**319.2**	115.1	12.5	5-HETE	60	-80.0	-10.0	-21.0	-12.0	EX
**345.2**	143.1	12.5	7-HDPA		-80.0	-10.0	-18.0	-15.0	
**319.2**	167.2	12.6	11.12-EET	60	-40.0	-10.0	-20.0	-10.0	EX
**343.2**	101.1	12.7	4-HDHA	60	-80.0	-10.0	-17.0	-15.0	EX
**330.2**	155.2	12.7	d11-8.9-EET	60	-30.0	-5.0	-20.0	-10.0	IS
**319.2**	167.0	12.8	8.9-EET	60	-40.0	-5.0	-20.0	-10.0	EX
**319.2**	191.1	13.0	5,6-EET	60	-50.0	-5.0	-20.0	-15.0	EX
**355.3**	193.2	6.6	d4-PGE_2_	120	-80.0	-10.0	-25.0	-16.0	IS
**380.3**	141.2	6.9	d5-RvD_2_	120	-80.0	-10.0	-23.0	-14.0	IS
**356.3**	115.2	7.4	d5-LXA_4_	120	-80.0	-10.0	-19.0	-14.0	IS
**339.3**	197.2	9.7	d4-LTB_4_	120	-80.0	-10.0	-22.0	-13.0	IS
**327.3**	116.1	12.4	d8-5*S*-HETE	120	-80.0	-10.0	-17.0	-10.0	IS
**330.2**	155.2	12.7	d11-8.9-EET	60	-30.0	-5.0	-20.0	-10.0	IS
**311.3**	267.1	14.8	d8-AA/20:4		-100.0	-10.0	-16.0	-18.0	IS

rt, retention time; DP, declustering potential; EP, entrance potential; CE, collision energy; CXP, collision cell exit potential; AA/20:4, arachidonic acid; AG, arachidonoyl glycerol; DHA, docosahexaenoic acid; DiHET, dihydroxyeicosatrienoic acid; DPA, docosapentaenoic acid; EET, epoxyeicosatrienoic acid; EPA, eicosapentaenoic acid; EX, external standard; HDHA, hydroxydocosahexaenoic acid; HDPA, hydroxydocosapentaenoic acid; HEPE, hydroxyeicosapentaenoic acid; (di)HETE, (di)hydroxyeicosatetraenoic acid; HODE, hydroxyoctadecadienoic acid; IS, internal standard; LT, leukotriene; LX, lipoxin; MaR, maresin; PD, protectin; PEA, palmitoylethanolamide; PG, prostaglandin; Rv, resolvin; TX, thromboxane.

**Table 2A:** QTRAP 6500^+^ parameters for targeted lipidomics (negative mode)

Negative mode	PC	PE	PG	PI	PS	CL	FFA
curtain gas [psi]	40	40	40	40	40	40	40
collision gas	Medium	Medium	Medium	Medium	Medium	Medium	
ion spray voltage [V]	-4500	-4500	-4500	-4500	-4500	-4500	-4500
heated capillary temperature [°C]	350	650	550	500	550	650	500
shealth gas [psi]	55	55	55	55	45	40	60
auxiliary gas [psi]	75	75	75	75	80	80	80
declustering potential [V]	-44	-50	-45	-50	-40	-44	-45
entrance potential [V]	-10	-10	-10	-10	-10	-10	-10
collision energy [eV]	-46	-38	-52	-62	-56	-40	
collision cell exit potential [V]	-11	-12	-18	-11	-20	-12	

**Table 2B:** QTRAP 6500^+^ parameters for targeted lipidomics (positive mode)

Positive mode	TG	DG	CE	TG/CE	So/Sa	(dh)Cer	HexCer	C1P	(dh)SM
curtain gas [psi]	40	40	40	40	40	40	40	40	40
collision gas	Low	Low	Low	Low	Medium	Medium	Medium	Medium	Medium
ion spray voltage [V]	5500	5500	5500	5500	5000	5000	5000	5000	5000
heated capillary temperature [°C]	400	400	350	375	500	500	500	500	500
shealth gas [psi]	60	60	55	60	40	40	40	40	40
auxiliary gas [psi]	70	70	70	70	40	40	40	40	40
declustering potential [V]	120	120	55	120/55	30	30	40	30	40
entrance potential [V]	10	10	10	10	10	10	5	10	10
collision energy [eV]	35	35	22	35/22	20	40	50	40	30
collision cell exit potential [V]	26	26	22	26/22	25	20	20	5	10

## Data Availability

The mass spectrometric lipidomics data generated in this study have been deposited in the Metabolomics Workbench database (an international repository for metabolomics data and metadata, metabolite standards, protocols, tutorials and training, and analysis tools [191]) under the accession code PR002799 [ST004429, ST004485, ST004540, ST004487; https://doi.org/http://dx.doi.org/10.21228/M8VN9M]. All other data supporting the findings of the present study are either shown in the manuscript and Supporting Information or available from the corresponding author upon reasonable request.

## Abbreviations

AA/20:4: arachidonic acid
ALOX: lipoxygenase
ALOX5AP: 5-lipoxygenase-activating protein
C1P: ceramide-1-phosphate
CE: cholesteryl ester
(dh)CER: (dihydro)ceramide
CL: cardiolipin
PTGS: cyclooxygenase
CYP: cytochrome P450 monooxygenase
DG: diacylglycerol
EET: epoxyeicosatrienoic acid
FCS: fetal calf serum
GPX4: glutathione peroxidase 4
GSH: glutathione
GSSG: glutathione disulfide
HBSS: Hank's buffered salt solution
(di)HETE: (di)hydroxyeicosatetraenoic acid
HexCer: hexosylceramide
4-HNE: 4-hydroxynonenal
IL: interleukin
IL-1ra: interleukin-1 receptor antagonist
iso: isomer
LPS: lipopolysaccharide
LT: leukotriene
MaR: maresin
MASLD: metabolic dysfunction-associated steatotic liver disease
MAP2K: mitogen-activated protein kinase kinase
MRM: multiple reaction monitoring
MUFA: monounsaturated fatty acid
MTT: 3-(4:5-dimethylthiazol-2-yl)-2:5-diphenyltetrazolium bromide
NF-κB: nuclear factor kappa B
NFKBI: nuclear factor kappa B inhibitor
NLRP3: NLR family pyrin domain containing 3
OA/18:1: oleic acid
oxLDL: oxidized low-density lipoprotein
PA/16:0: palmitic acid
PBMC: peripheral blood mononuclear cell
PC: phosphatidylcholine
PD: protectin
PE: phosphatidylethanolamine
PG: phosphatidylglycerol
PI: phosphatidylinositol
PLA_2_: phospholipase A_2_: PS: phosphatidylserine
PUFA: polyunsaturated fatty acid
ROS: reactive oxygen species
RIPK1: receptor interacting serine/threonine kinase 1
Rv: resolvin
SA: sphinganine
SACM: *Staphylococcus aureus-*conditioned medium
SFA: saturated fatty acid
(dh)SM: (dihydro)sphingomyelin
So: sphingosine
SPM: specialized pro-resolving mediator
TG: triglyceride
TNF: tumor necrosis factor
TX: thromboxane
