# Supplementary material for: Context-dependent Activities of Mitrephorone Link Lipid Redirection, Anti-inflammatory Action, and Ferroptosis Control to Hepatocyte Protection
Source: Theranostics. 2026 Mar 17;16(10):5406–39. doi: 10.7150/thno.127100 (PMC13080623; doi:10.7150/thno.127100)
Supplement: Supplementary file 1 — Supplementary Figures S1-S8 and Table S1. [file thnov16p5406s1.pdf]

Supplementary Information

Context-dependent activities of mitrephorone link lipid redirection, anti-inflammatory action, and ferroptosis control to hepatocyte protection

Lorenz Waltl[1,2], Lukas A. Wein[3], Leonhard Bereuter[4], Fengting Su[4], Henriett Barta[4], Loc Le Xuan[1], David Holubek[4], Zahra Mahmoudi Eshkaftaki[4], Katharina Puskac[4], Anita Siller[5], Peter Schlenke[6], Harald Schennach[5], Eva-Maria Pferschy-Wenzig[4], Hans Schött[4], Silvia Racedo[4], Solveigh C. Koeberle[1,4], Thomas Magauer[3], Andreas Koeberle[1,4,*]

[1] Michael Popp Institute and Center for Molecular Biosciences (CMBI), University of Innsbruck, Mitterweg 24, 6020 Innsbruck, Austria

[2] Institute of Human Genetics, Medical University of Innsbruck, Peter-Mayr-Straße 1, 6020 Innsbruck, Austria

[3] Department of Organic Chemistry and Center for Molecular Biosciences (CMBI) University of Innsbruck, Innrain 80-82, 6020 Innsbruck, Austria

[4] Institute of Pharmaceutical Sciences and Excellence Field BioHealth, NAWI Graz, University of Graz, Beethovenstraße 8, 8010 Graz, Austria

[5] Central Institute for Blood Transfusion and Immunology, Tirol Kliniken GmbH, Anichstraße 35, 6020 Innsbruck, Austria

[6] Clinical Department of Blood Group Serology and Transfusion Medicine, Medical University of Graz, Auenbruggerplatz 48, 8036 Graz, Austria

*Corresponding author: Tel.: +43 316 380 – 8630, E-mail: andreas.koeberle@uni-graz.at (Andreas Koeberle).

Supplementary tables

Table S1. Human-specific primers for qPCR

Gene	RefSeq-ID	Sequence (5' → 3')
FTH1	NM_002032.3	fwd: TGAAGCTGCAGAACCAACGAGG<br>rev: GCACACTCCATTGCATTCAGCC
FTL	NM_000146.4	fwd: TACGAGCGTCTCCTGAAGATGC<br>rev: GGTTCAGCTTTTTCTCCAGGGC
SLC40A1 (FPN1)	NM_014585.6	fwd: CTACTTGGGGAGATCGGATGT<br>rev: CTGGGCCACTTTAAGTCTAGC
SLC11A2 (DMT1)	NM_000617.3	fwd: AGCTCCACCATGACAGGAACCT<br>rev: TGGCAATAGAGCGAGTCAGAACC
STEAP3	NM_018234.3	fwd: TGCAAACTCGCTCAACTGGAGG<br>rev: AGGCAGGTAGAACTTGTAGCGG
SLC25A37 (MFRN1)	NM_016612.4	fwd: CCACATGACAGCAGGAGCGATG<br>rev: CTTGTGTACTGGGCTTTGGGATC
SLC25A28 (MFRN2)	NM_031212.4	fwd: AGCCACTGTCACCACGCACATG<br>rev: GTCAGGCTGTAGACTCTGCATC
FECH	NM_000140.5	fwd: TCTTCTTGGACCGAGACCTCATG<br>rev: TCCAATCCTGCGGTACTGCTCT
GPX4	NM_002085.3	fwd: AGGCAAGACCGAAGTAAACTACAC<br>rev: TACACTCTCTTCGTTACTCCCTGGCT
SLC7A11	NM_014331.3	fwd: TGCTCTTCTCTGGAGACCTCGAC<br>rev: ACAGTGGCACCTTGAAAGGACG
PTGS2	NM_000963.4	fwd: GTTCCACCCGCAGTACAGAA<br>rev: AGGGCTTCAGCATAAAGCGT
PRDX1	NM_002574.2	fwd: TCGCGAGATCCCTACTGGC<br>rev: ACCAGTCCCAACACAAGTCG
PRDX6	NM_004905.3	fwd: CAGCTACCACTGGCAGGAACTT<br>rev: GGAAGGACCATCACACTATCCC
CAT	NM_001752.4	fwd: GTGCGGAGATTCAACACTGCCA<br>rev: CGGCAATGTTCTCACACAGACG
GCH1	NM_000161.3	fwd: GCCATGCAGTTCTTCACCAAGG<br>rev: ATGGAACCAAGTGATGCTCACAC
SQOR	NM_021199.4	fwd: GCTGACCTGATGCCTTTCCTGT<br>rev: CCAGAAGCCATCCAAGATGAGC
POR	NM_001395413	fwd: ACTCTGCTCTCGTCAACCAGCT<br>rev: TGGGTGCTTCTTGTTGGACTCC
FSP1/AIFM1	NM_004208.4	fwd: GACTCCTTCCACCACAATGTGG<br>rev: CAGCACCATCTGGTTCTTCAGG
ACSL4	NM_022977.3	fwd: GCTATCTCCTCAGACACACCGA<br>rev: AGGTGCTCCAACTCTGCCAGTA
LPCAT3	NM_005768.6	fwd: CAGGATACCTGGTCTGCTTCCA<br>rev: TGAAGAGCCAGTGGATGGTCTG
GAPDH	NM_002085.3	fwd: AGGCAAGACCGAAGTAAACTACAC<br>rev: TACACTCTCTTCGTTACTCCCTGGCT

Supplementary figures

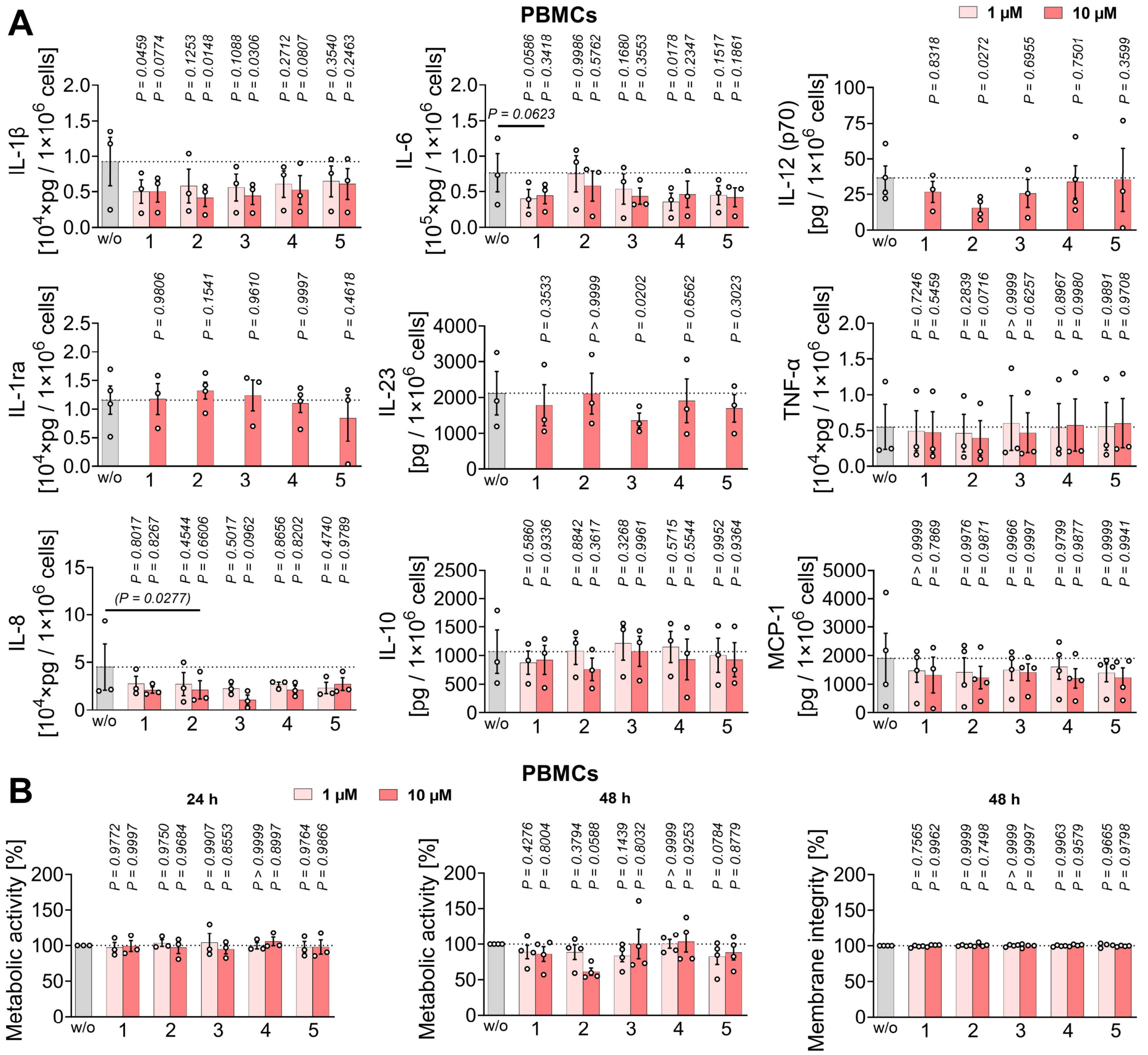

Supplementary Figure S1. Effect of mitrephorones 1–5 on cytokine expression, metabolic activity, and membrane integrity of PBMCs. (**A**) Cytokine and chemokine levels in PBMCs (1.4×10^6) preincubated with vehicle (DMSO, 0.1%) or mitrephorones for 30 min and stimulated with LPS for 4 h (TNF-α, IL-8) or 18 h (IL-1β, IL-6, IL-10, MCP-1, IL-12 (p70), IL-23, IL-1ra). (**B**) PBMCs (2×10^5 for metabolic activity; 4×10^6 for membrane integrity) were preincubated with vehicle (DMSO, 0.5% for metabolic activity; 0.1% for membrane integrity) or mitrephorones for 24 or 48 h. Metabolic activity (MTT assay) and membrane integrity were determined. Mean ± SEM and single data from *n* = 3-4 independent experiments. *P* values given *vs.* vehicle control; repeated measures one-way ANOVA of log data (**A**, **B**) or mixed-effects model (REML) ANOVA of log data (**A**) + Dunnett's *post hoc* tests, or two-tailed paired Student's *t* test of log data for pairwise comparisons as indicated by bars (**A**). The vehicle controls shown in **A** and **B** are identical to those published previously [1].

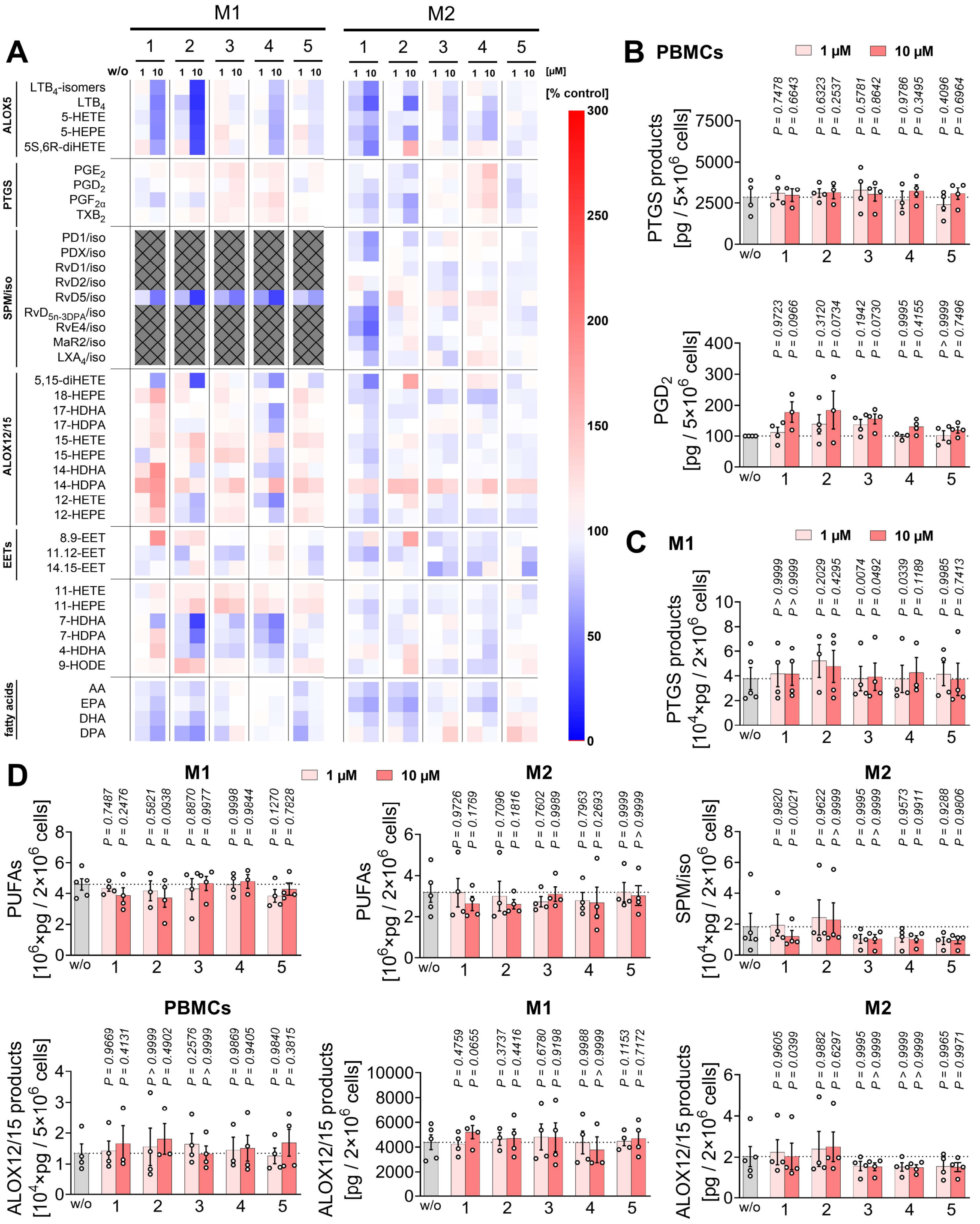

Supplementary Figure S2. Effect of mitrephorones 1–5 on lipid mediator profiles of activated human PBMCs and macrophages. (**A**, **C**, **D**) M1-like or M2-like macrophages (2×10^6) were preincubated with vehicle (DMSO, 0.1%) or mitrephorones for 15 min and stimulated with SACM (1%) for 180 min. (**A**) Heatmap of mean percentage changes in oxylipin and fatty acid levels relative to vehicle. Lipid mediators are grouped as ALOX5 products, PTGS products, SPM/iso, monohydroxylated ALOX12/15 products, EETs, and free PUFAs. Crossed boxes indicate lipid mediators that were not detected. (**B**, **D**) PBMCs (5×10^6) were preincubated with vehicle (DMSO, 0.1%) or mitrephorones for 10 min and treated with A23187 for 10 min. (**B**) Levels of PTGS products and PGD_2 in PBMCs. (**C**) PTGS product formation in M1-like macrophages. (**D**) Levels of PUFAs, ALOX12/15 products, and SPM/iso. Mean (**A**) or mean ± SEM and single data (**B-D**) from n = 3-4 independent experiments. *P* values given *vs.* vehicle control; mixed-effects model (REML) ANOVA of log data + Dunnett's *post hoc* tests. The vehicle controls shown in **A-D** are identical to those published previously [1].

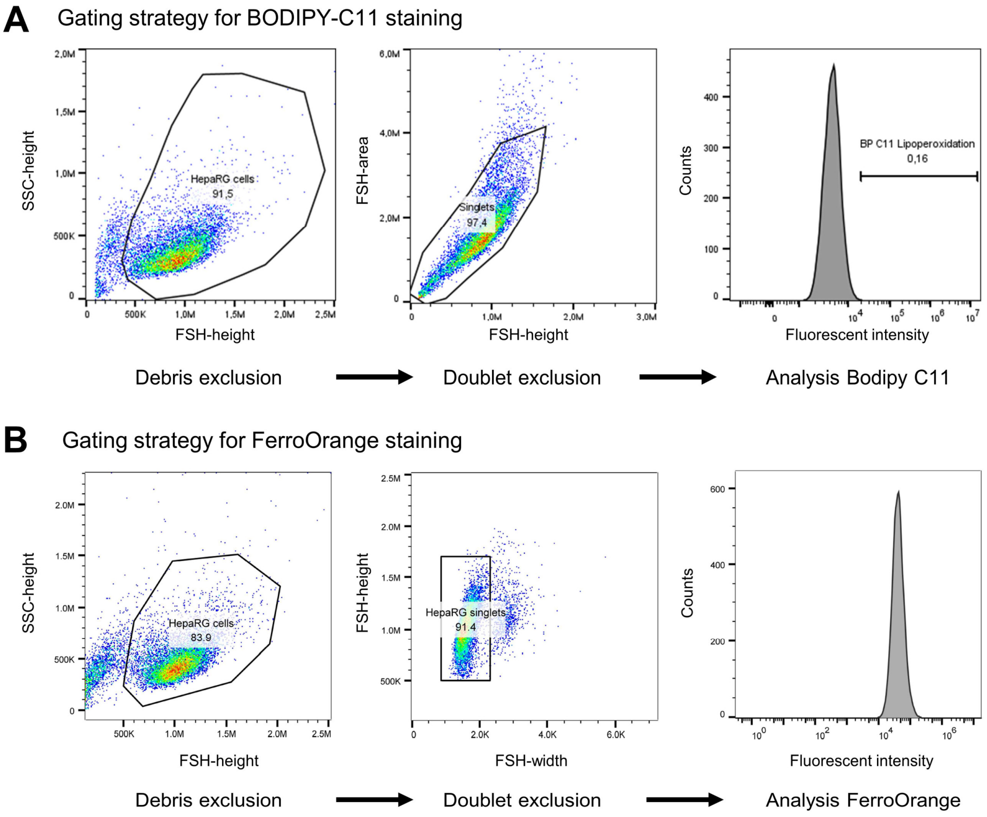

Supplementary Figure S3. Gating strategies. (A) Figure 3A, B. (B) Figure 4B.

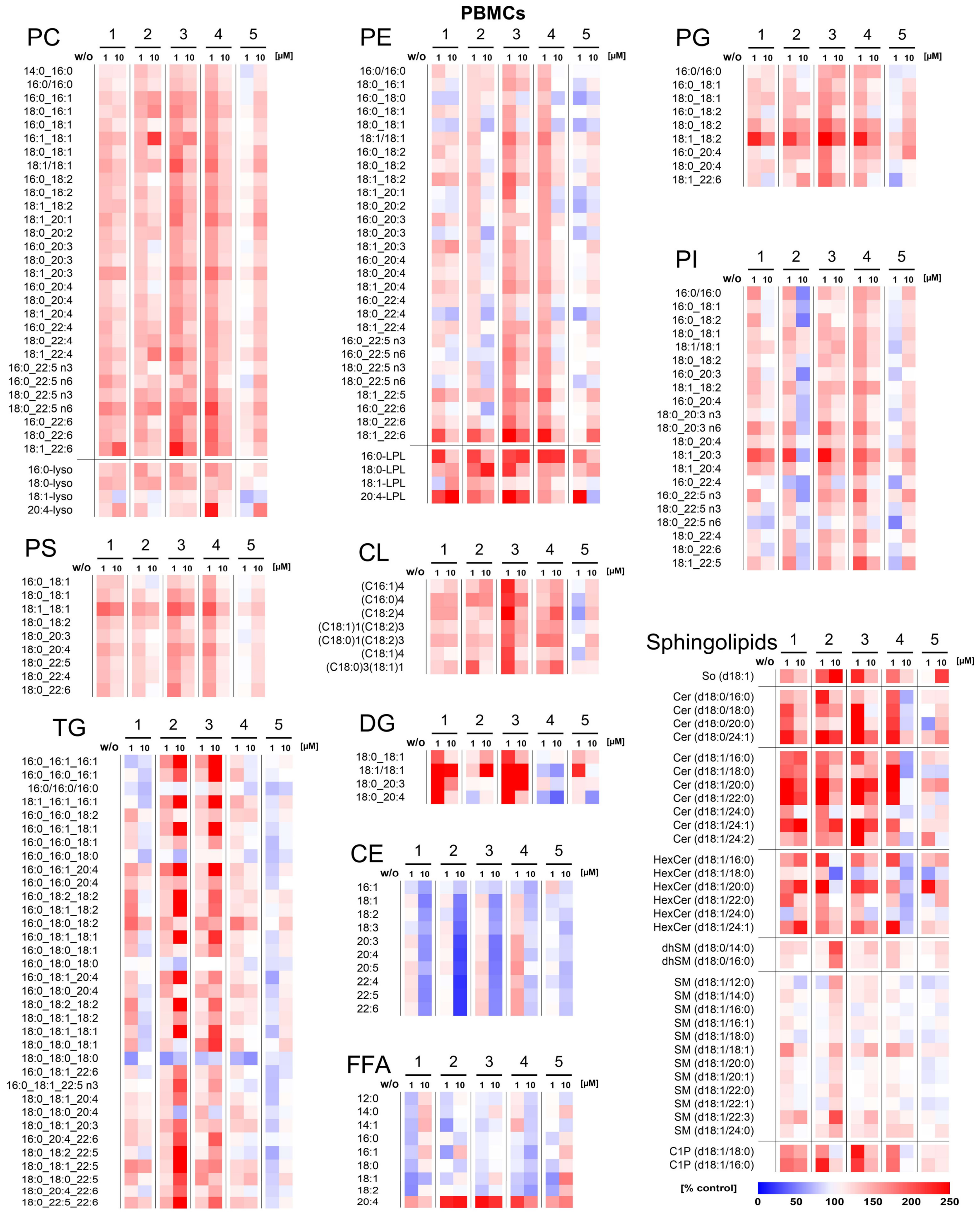

Supplementary Figure S4. Effect of mitrephorones 1–5 on the lipid composition of human PBMCs. Cells (4×10^6) were treated with vehicle (DMSO, 0.1%) or mitrephorones for 48 h. Heatmaps depict mean percentage changes in the amount of lipid species relative to vehicle. Mean from n = 2 (CL **3** – 1 µM) or n = 3 independent experiments. The vehicle controls shown are identical to those published previously [1].

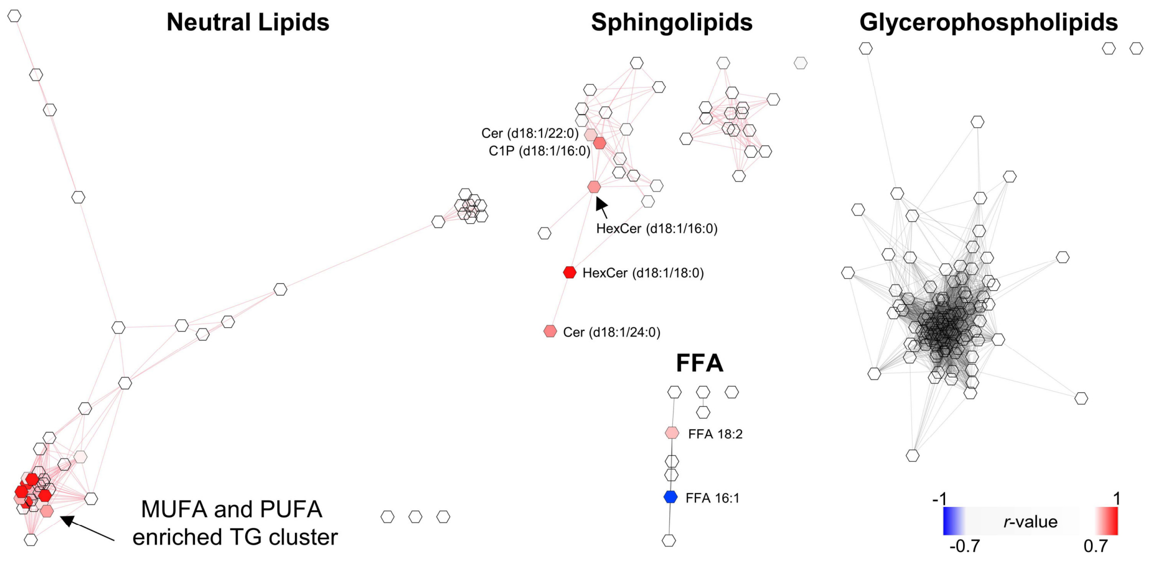

Supplementary Figure S5. Correlation between 1-5-induced changes in PBMC relative lipid proportions and ferroptosis protection in hepatocytes. Correlation-based lipid network showing positive lipid–lipid correlations ($r \geq 0.7$) among neutral lipids, sphingolipids, glycerophospholipids, or free fatty acids (FFAs). Lipid species are represented as nodes (see Figure S4 for detailed information). The network combines data from PBMCs treated with compounds **1**–**5** (1 and 10 µM each) for 48 h and was calculated from the mean percentage changes in relative lipid proportions relative to vehicle control from n = 2 (CL **3** – 1 µM) or n = 3 independent experiments. Node color indicates the strength and direction of Pearson correlations between mitrephorone **1**–**5** (1 µM)-induced changes in the proportion of PBMC lipid species and the mean percentage changes in the metabolic activity of RSL3-treated HepaRG cells (Figure 2A) within 48 h relative to vehicle control: positive correlations ($r \geq 0.7$) are shown in red, negative correlations ($r \leq -0.7$) in blue; n = 2 (CL **3** – 1 µM) or n = 3 independent experiments.

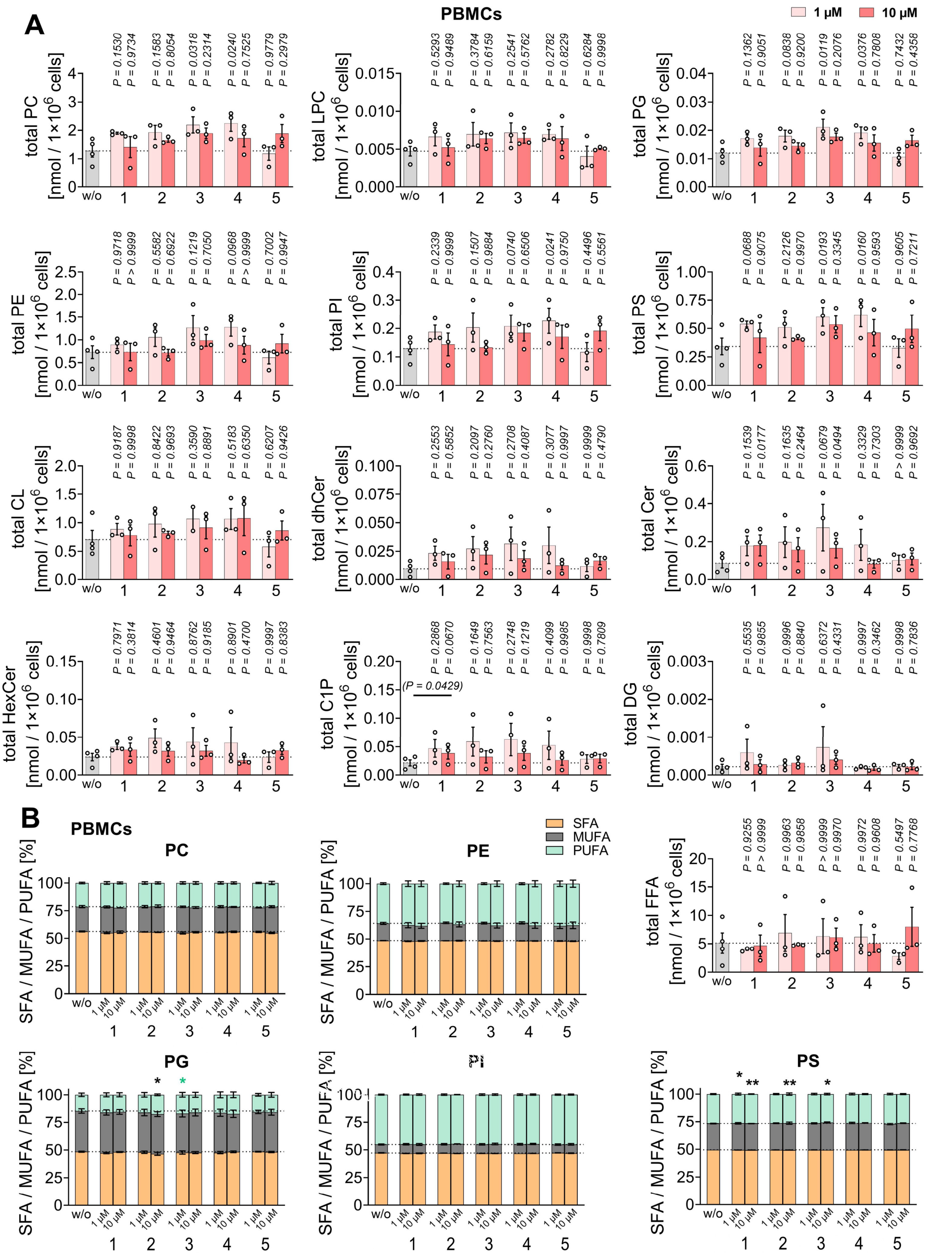

Supplementary Figure S6. Effect of mitrephorones 1-5 on the amount of lipid subclasses and the ratio of phospholipid-bound SFAs, MUFAs, and PUFAs in PBMCs. Cells (4×10^6) were treated with vehicle (DMSO, 0.1%) or mitrephorones for 48 h. (**A**) Amounts of lipid subclasses in pg per 1×10^6 cells. (**B**) Ratios of SFAs, MUFAs, and PUFAs in glycerophospholipids. Mean ± SEM (**B**) and single data (**A**) from *n* = 2 (**A** CL **3** – 1 µM) or *n* = 3 (**A**, **B**) independent experiments. *P* values given *vs.* vehicle control (**A**) or **P* < 0.05 *vs.* vehicle control; mixed-effects model (REML) ANOVA (**B**) of log data (**A**) + Dunnett's *post hoc* tests. The vehicle controls shown are identical to those published previously [1].

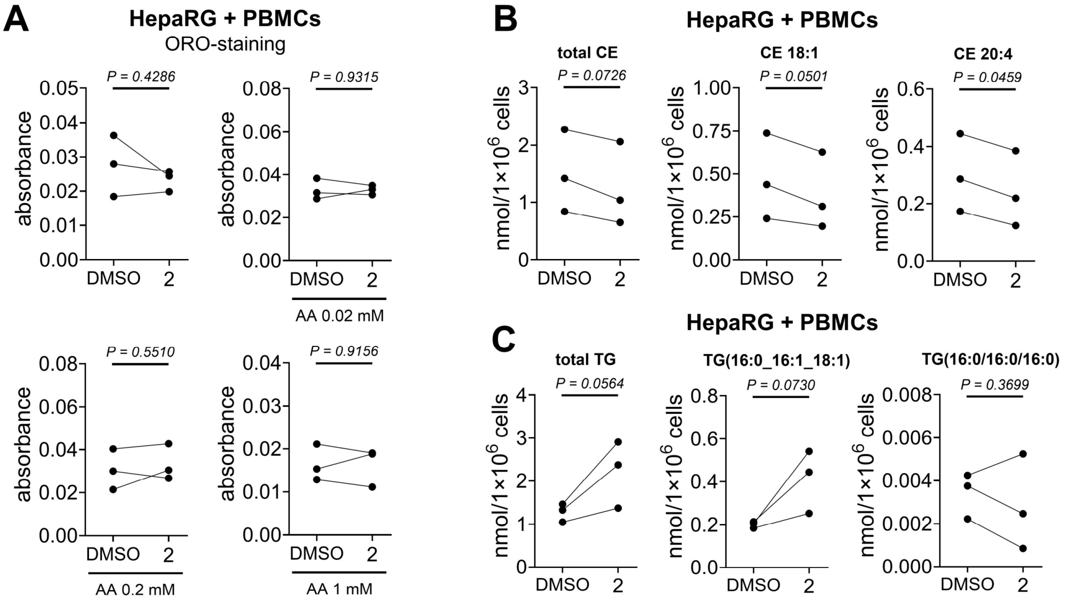

Supplementary Figure S7. Effect of mitrephorone 2 on lipid droplet staining and neutral lipid content in unchallenged and AA/20:4-challenged PBMCs. HepaRG cells and PBMCs (1:1) were co-cultured and preincubated with or without AA/20:4 for 24 h. Cells were then treated with vehicle (DMSO, 0.1%) or **2** (10 µM) for an additional 24 h. (**A**) Absorbance of organic extracts from Oil-Red-O (ORO)-stained cells. (**B**) Content of CE and selected CE species. (**C**) Content of TG and selected TG species. Interconnected single data from $n = 3$ independent experiments. P values given *vs.* vehicle control; two-tailed paired Student's t tests of log data.

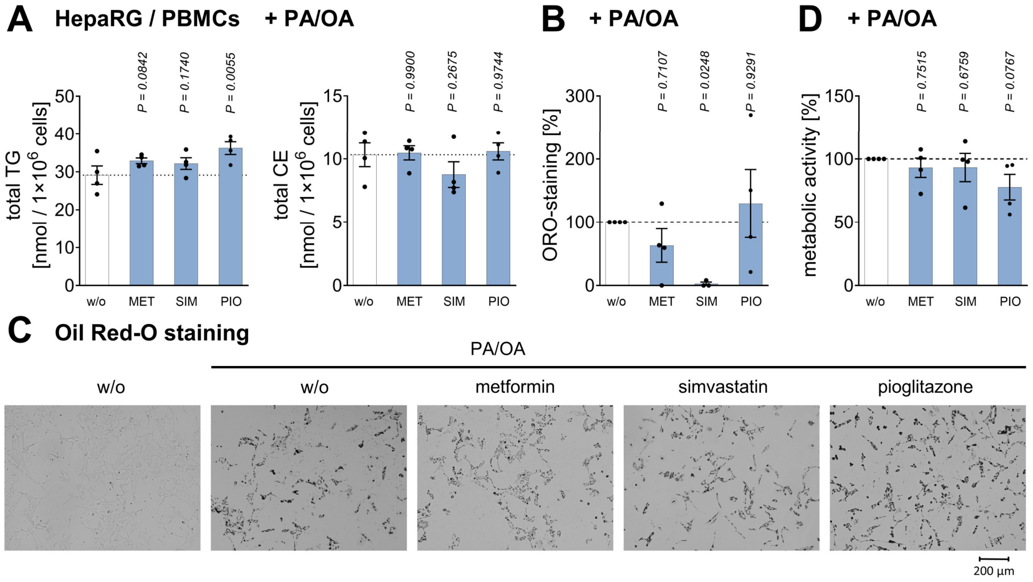

Supplementary Figure S8. Effect of metformin, simvastatin, and pioglitazone on lipid overload in cocultures of hepatocytes and PBMCs. HepaRG cells and PBMCs were co-cultured (1:1) and preincubated with PA/16:0 and OA/18:1 (1 mM) for 24 h. Cells were then treated with vehicle (DMSO, 0.1%) or metformin (1 mM), simvastatin (1 µM), or pioglitazone (100 µM) and incubated for another 24 h. (**A**) Total TG and CE content. (**B**) ORO staining – absorbance of organic extracts. (**C**) ORO staining – microscopic images. (**D**) Metabolic activity (MTT assay). Mean ± SEM and single data from *n* = 3-4 independent experiments. *P* values given *vs.* vehicle control; repeated measures one-way ANOVA of log data + Dunnett's *post hoc* tests.

References

1. Waltl L, Speck K, Wildermuth R, Haut F, Permann P, D'Avino D, et al. Reorganization of innate immune cell lipid profiles by bioinspired meroterpenoids to limit inflammation. bioRxiv. 2024: doi: https://doi.org/10.1101/2024.05.24.595516.
