# Supplementary material for: Context-dependent Activities of Mitrephorone Link Lipid Redirection, Anti-inflammatory Action, and Ferroptosis Control to Hepatocyte Protection
Source: Theranostics. 2026 Mar 17;16(10):5406–39. doi: 10.7150/thno.127100 (PMC13080623; doi:10.7150/thno.127100)
Supplement: Supplementary file 2 — Tables S2-S6 as well as raw images. [file thnov16p5406s2.zip › Raw_images.pdf]

Raw images for
ORIGINAL ARTICLE

Context-dependent activities of mitrephorone link lipid redirection, anti-inflammatory action, and ferroptosis control to hepatocyte protection

Lorenz Waltl[1,2], Lukas A. Wein[3], Leonhard Bereuter[4], Fengting Su[4], Henriett Barta[4], Loc Le Xuan[1], David Holubek[4], Zahra Mahmoudi[4], Katharina Puskac[4], Anita Siller[5], Peter Schlenke[6], Harald Schennach[5], Eva-Maria Pferschy-Wenzig[4], Hans Schött[4], Silvia Racedo[4], Solveigh C. Koeberle[1,4], Thomas Magauer[3], Andreas Koeberle[1,4,*]

[1] Michael Popp Institute and Center for Molecular Biosciences (CMBI), University of Innsbruck, Mitterweg 24, 6020 Innsbruck, Austria

[2] Institute of Human Genetics, Medical University of Innsbruck, Peter-Mayr-Straße 1, 6020 Innsbruck, Austria

[3] Department of Organic Chemistry and Center for Molecular Biosciences (CMBI) University of Innsbruck, Innrain 80-82, 6020 Innsbruck, Austria

[4] Institute of Pharmaceutical Sciences and Excellence Field BioHealth, NAWI Graz, University of Graz, Beethovenstraße 8, 8010 Graz, Austria

[5] Central Institute for Blood Transfusion and Immunology, Tirol Kliniken GmbH, Anichstraße 35, 6020 Innsbruck, Austria

[6] Clinical Department of Blood Group Serology and Transfusion Medicine, Medical University of Graz, Auenbruggerplatz 48, 8036 Graz, Austria

*Corresponding author: Tel.: +43 316 380 – 8630, E-mail: andreas.koeberle@uni-graz.at (Andreas Koeberle).

Figure 2C – uncropped blots / PBMCs – 15 min

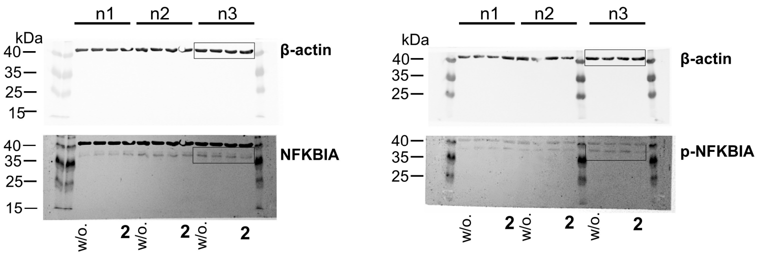

Figure 2C – uncropped blots / PBMCs – 1 h

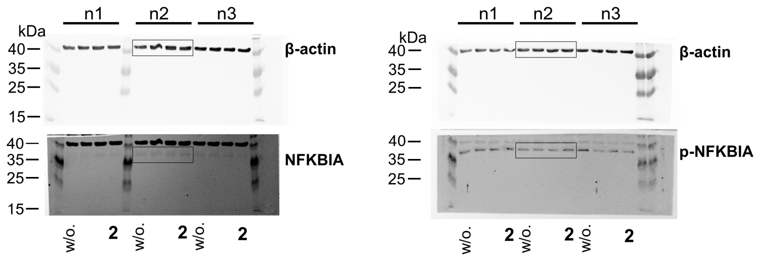

Uncropped Western blots corresponding to Figure 2C. The vehicle controls shown in A and B are identical to those published previously [1].

Figure 3C – individual microscopic images / HepaRG cells

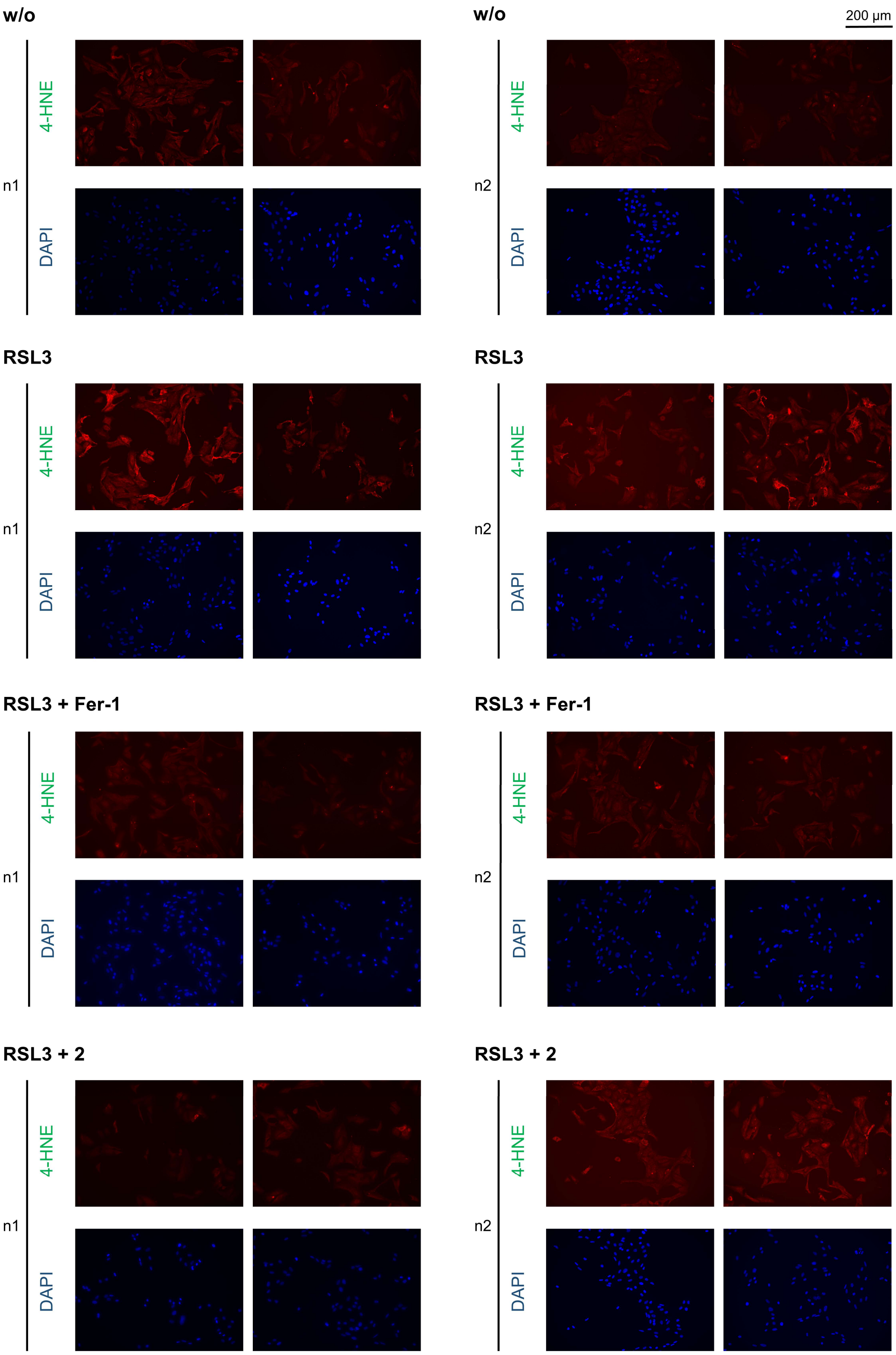

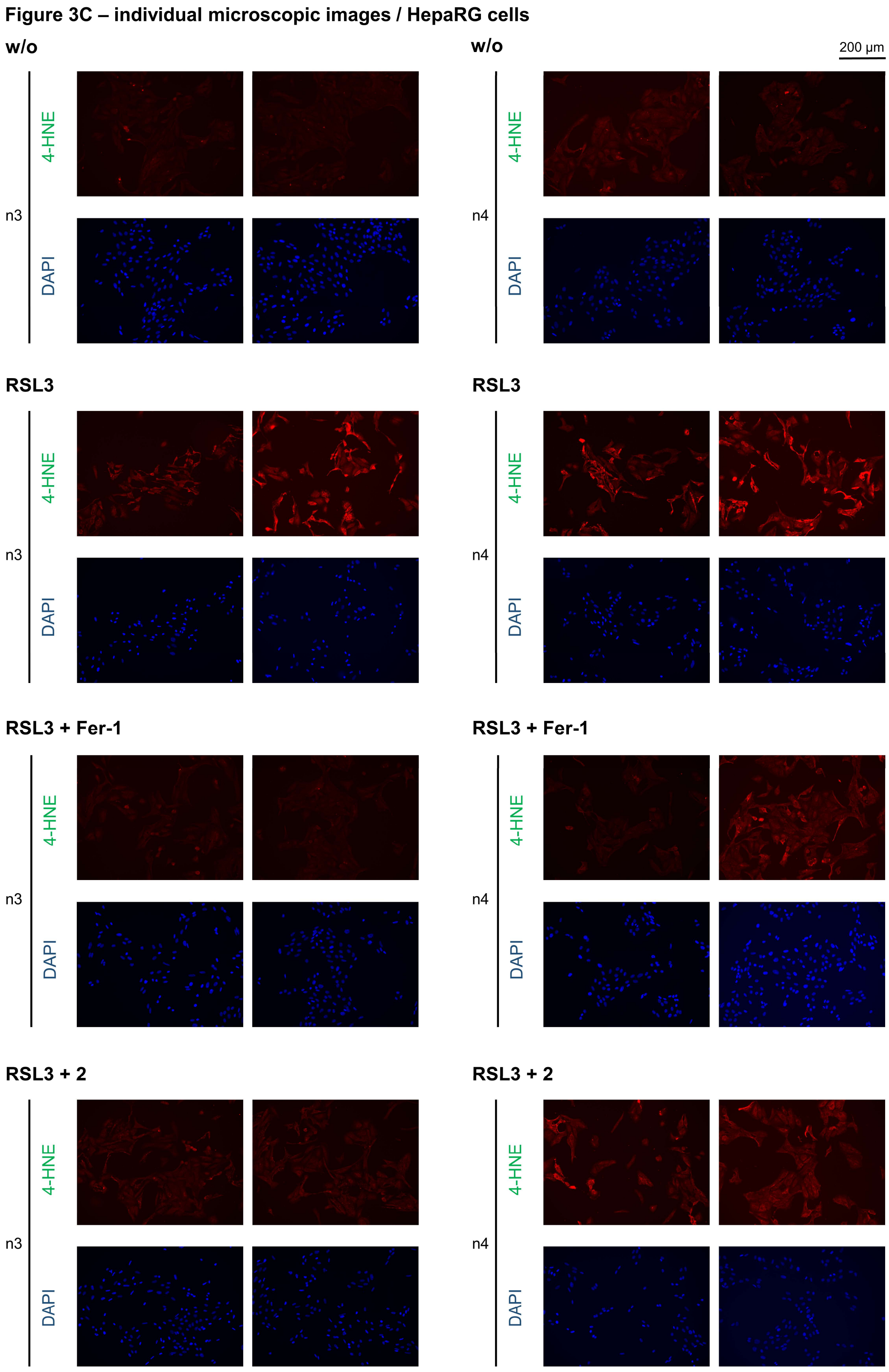

Individual microscopic images corresponding to Figure 3C.

Figure 4F – uncropped blots / HepaRG cells

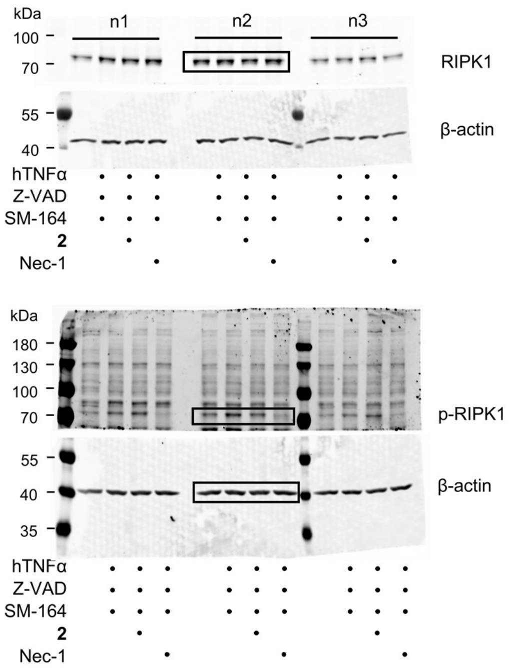

Figure 4I – uncropped blots / HepaRG cells

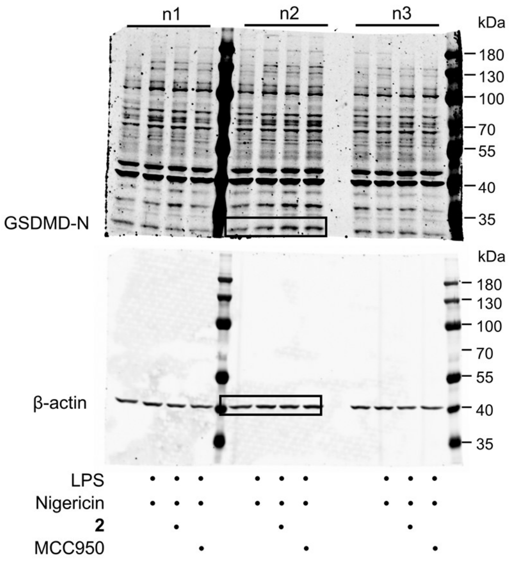

Uncropped Western blots corresponding to Figure 4F and Figure 4I.

Figure 5C – uncropped blots / HEK-293 cells

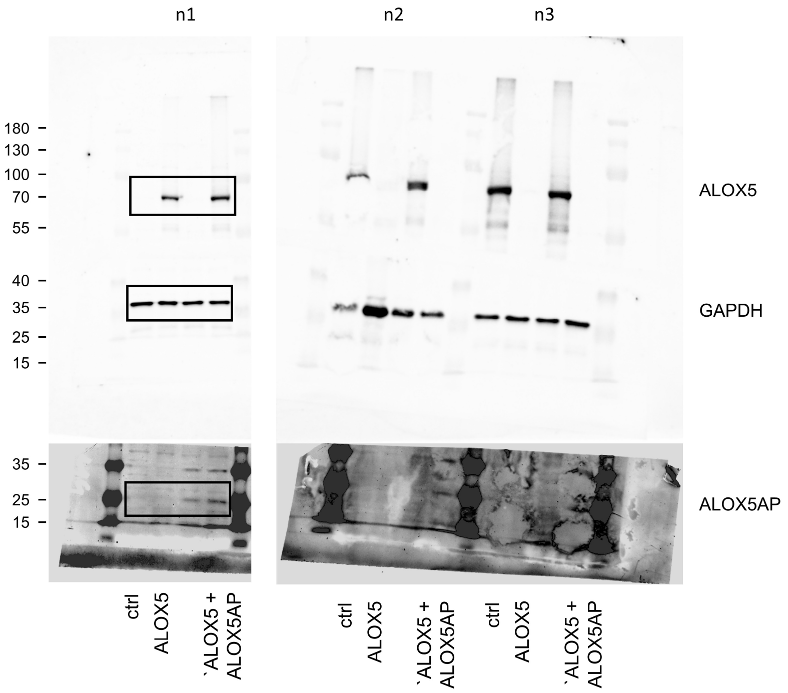

Uncropped Western blots corresponding to Figure 5C.

Figure 7C – uncropped blots / PBMCs

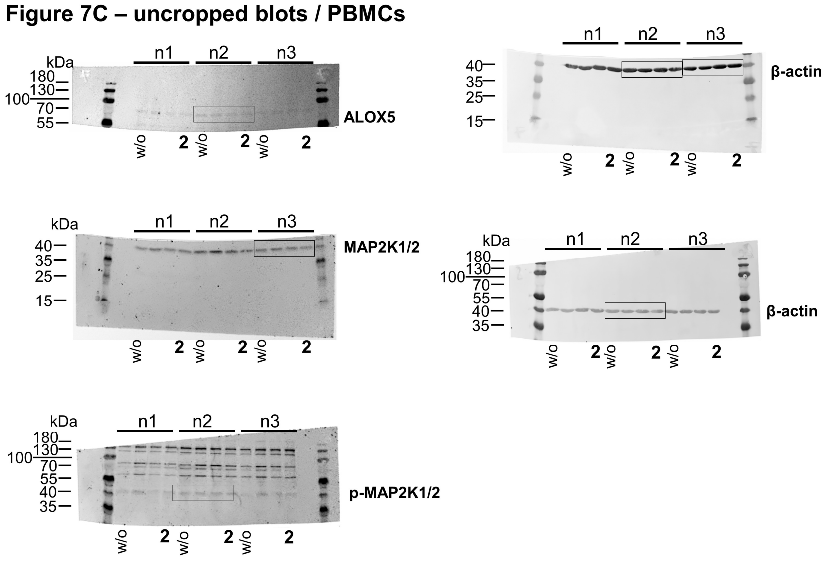

Uncropped Western blots corresponding to Figure 7C. The vehicle controls are identical to those published previously [1].

References

1. Waltl L, Speck K, Wildermuth R, Haut F, Permann P, D'Avino D, et al. Reorganization of innate immune cell lipid profiles by bioinspired meroterpenoids to limit inflammation. bioRxiv. 2024: doi: https://doi.org/10.1101/2024.05.24.595516.
